# Supplementary material for: Effects of computerised clinical decision support systems (CDSS) on nursing and allied health professional performance and patient outcomes: a systematic review of experimental and observational studies
Source: BMJ Open. 2021 Dec 15;11(12):e053886. doi: 10.1136/bmjopen-2021-053886 (PMC8679061; doi:10.1136/bmjopen-2021-053886)
Supplement: Supplementary data [file bmjopen-2021-053886supp001.pdf]

SUPPLEMENTARY MATERIAL LIST

Supplementary Table 1: Search strategies, to February 12, 2021

Supplementary Table 2: Risk of Bias assessment justifications using Effective Practice Organisation of Care (EPOC)'s tool

Supplementary Table 3: Summary of patient care process results

Supplementary Table 4 Summary of patient care outcomes results

Supplementary Table 5: Summary of Health professionals' knowledge, beliefs and behaviour results

Supplementary Table 6: Summary of adverse events results

Supplementary Table 7: Summary of economic costs and consequences results

Supplementary Table 1: Search strategies

1. **Ovid MEDLINE(R) ALL, 1946 to February 12, 2021 Search Strategy**

```
1     exp Decision Making/ (207895)
2     decision support techniques/ (20911)
3     (decision* adj2 making).ti,ab,kf. (159754)
4     (decision* adj2 support*).ti,ab,kf. (24230)
5     (decision* adj2 aid*).ti,ab,kf. (6501)
6     or/1-5 (354546)
7     exp Computers/ (79322)
8     exp information systems/ (238259)
9     exp Informatics/ (537355)
10     Internet/ (74916)
11     Software/ (112580)
12     Cell Phone/ (8821)
13     Mobile Applications/ (6962)
14     exp Telemedicine/ (32559)
15     Medical Records Systems, Computerized/ (19076)
16     exp Electronic Health Records/ (21793)
17     computer*.ti,ab,kf. (313610)
18     electronic*.ti,ab,kf. (291368)
19     (internet or web or online or on-line).ti,ab,kf. (310071)
20     (software or computer program*).ti,ab,kf. (193359)
21     (automate* or automation).ti,ab,kf. (136436)
22     (pda or pdas).ti,ab,kf. (13229)
23     personal digital assistant*.ti,ab,kf. (1012)
24     (app or apps).ti,ab,kf. (31717)
25     (application* adj2 mobile*).ti,ab,kf. (4834)
26     (iPad* or iPhone* or smartphone* or smart phone* or smart device*
    or mobile phone or android phone* or cellphone* or cell
    phone*).ti,ab,kf. (26450)
27     (tablet adj2 (pc or device* or comput*)).ti,ab,kf. (1603)
```

28 ((hand held or handheld) adj2 (pc or device* or comput*)).ti,ab,kf. (2669)

29 (telehealth or telecare or telemedicine or ehealth or mhealth).ti,ab,kf. (29130)

30 or/7-29 (1674343)

31 6 and 30 (66042)

32 exp Decision Making, Computer-Assisted/ (149528)

33 Decision Support Systems, Clinical/ (8302)

34 (computer assisted adj2 (decision* or diagnos* or therap* or support or treatment? or management)).ti,ab,kf. (1545)

35 (computer aided adj2 (decision* or diagnos* or therap* or support or treatment? or management)).ti,ab,kf. (3921)

36 (decision adj2 support adj2 (system* or tool*)).ti,ab,kf. (9917)

37 (decision making adj2 (system* or tool*)).ti,ab,kf. (2560)

38 Expert Systems/ (3420)

39 (expert adj2 system*).ti,ab,kf. (3613)

40 Reminder Systems/ (3568)

41 ((computer* or electronic* or CDSS) adj2 (reminder* or alert*)).ti,ab,kf. (1210)

42 ((medication or medicine or treatment or therapy) adj2 (reminder* or alert*)).ti,ab,kf. (857)

43 reminder system*.ti,ab,kf. (875)

44 Medical Order Entry Systems/ (2303)

45 ((computer* or electronic*) adj2 order entry).ti,ab,kf. (1874)

46 (computer adj2 decision support*).ti,ab. (412)

47 CPOE.ti,ab,kf. (1139)

48 or/32-47 (177952)

49 31 or 48 [all computerised clinical decision support systems terms] (228840)

50 Allied Health Personnel/ (11925)

51 Allied Health Occupations/ (587)

52 Physical Therapist Assistants/ (16)

53 Physical Therapy Specialty/ (2889)

54 Speech-Language Pathology/ (3172)

55 Occupational Therapy/ (13482)

56 Nutritionists/ (1290)

57 dietetics/ (7837)

58 Anesthesiologists/ (1163)

59 podiatry/ (2273)

60 exp Osteopaths/ (321)

61 osteopathic physicians/ (321)

62 anesthesiologist*.ti,ab,kf. (22810)

63 podiatrist*.ti,ab,kf. (910)

64 prosthetist*.ti,ab,kf. (397)

65 chiropodist*.ti,ab,kf. (132)

66 orthoptist*.ti,ab,kf. (319)

67 orthotist*.ti,ab,kf. (220)

68 osteopath*.ti,ab,kf. (5983)

69 radiographer*.ti,ab,kf. (1803)

70 art therapist*.ti,ab,kf. (89)

71 drama therapist*.ti,ab,kf. (3)

72 music therapist*.ti,ab,kf. (368)

73 (allied adj2 health adj2 (profession* or worker* or personnel or occupation* or staff)).ti,ab,kf. (3421)

74 ((physical or occupational or language or speech or physio*) adj2 therap*).ti,ab,kf. (50227)

75 physiotherapist*.ti,ab,kf. (8544)

76 dietetic*.ti,ab,kf. (9828)

77 dietitian*.ti,ab,kf. (6580)

78 nutritionist*.ti,ab,kf. (3020)

79 Patient care team/ (66483)

80 ((multidisciplinary or multi-disciplinary or multiprofessional or multi-professional or interdisciplinary or interprofessional) adj2 team*).ti,ab,kf. (32126)

81 Emergency Medical Technicians/ (5756)

82 Emergency Medical Services/ (43736)

83 Ambulances/ (6210)

84 Air Ambulances/ (2874)

85 paramedic*.ti,ab,kf. (8537)

86 HEMS.ti,ab,kf. (767)

87 ems.ti,ab,kf. (13017)

88 emt.ti,ab,kf. (25232)

89 prehospital.ti,ab,kf. (13136)

90 pre-hospital.ti,ab,kf. (4836)

91 first responder*.ti,ab,kf. (2449)

92 emergency medical technician*.ti,ab,kf. (1168)

93 emergency services.ti,ab,kf. (4115)

94 ambulance*.ti,ab,kf. (11269)

95 field triage.ti,ab,kf. (275)

96 out-of-hospital.ti,ab,kf. (11317)

97 (nurse or nurses or nursing).ti,ab,kf. (462330)

98 exp nurses/ (89638)

99 exp nursing staff/ (67063)

100 Midwifery/ (19460)

101 (midwif* or midwiv*).ti,ab,kf. (25895)

102 or/50-101 [allied health professionals or nurses or midwives] (836031)

103 49 and 102 [all CDSS and allied health professionals or nurses or midwives] (9549)

2. Embase Classic+Embase 1947 to February 12, 2021 Search Strategy

----

1 exp Decision Making/ (399525)

2 decision support techniques/ (20092)

3 (decision* adj2 making).ti,ab,kw. (218454)

4 (decision* adj2 support*).ti,ab,kw. (32940)

5 (decision* adj2 aid*).ti,ab,kw. (9487)

6 or/1-5 (504731)

7 exp Computer/ (159861)

8 exp information system/ (166084)

9 exp information science/ (113984)

10 Internet/ (112888)

11 Software/ (79162)

12 mobile phone/ (17899)

13 smartphone/ (15041)

14 Mobile Application/ (13261)

15 exp Telemedicine/ (47236)

16 electronic medical record system/ (1535)

17 exp Electronic Health Record/ (21723)

18 computer*.ti,ab,kw. (407323)

19 electronic*.ti,ab,kw. (350647)

20 (internet or web or online or on-line).ti,ab,kw. (418206)

21 (software or computer program*).ti,ab,kw. (321717)

22 (automate* or automation).ti,ab,kw. (197239)

23 (pda or pdas).ti,ab,kw. (18450)

24 personal digital assistant*.ti,ab,kw. (1217)

25 (app or apps).ti,ab,kw. (43764)

26 (application* adj2 mobile*).ti,ab,kw. (6399)

27 (iPad* or iPhone* or smartphone* or smart phone* or smart device* or android phone* or cellphone* or cell phone* or mobile phone*).ti,ab,kw. (38430)

28 (tablet adj2 (pc or device* or comput*)).ti,ab,kw. (2528)

29 ((hand held or handheld) adj2 (pc or device* or comput*)).ti,ab,kw. (3833)

30 (telehealth or telecare or telemedicine or ehealth or mhealth).ti,ab,kw. (35247)

31 or/7-30 (1897765)

32 6 and 31 (80108)

33 exp decision support system/ (27016)

34 clinical decision support system/ (3594)

35 (computer assisted adj2 (decision* or diagnos* or therap* or support or treatment? or management)).ti,ab,kw. (2316)

36 (computer aided adj2 (decision* or diagnos* or therap* or support or treatment? or management)).ti,ab,kw. (5577)

37 (decision adj2 support adj2 (system* or tool*)).ti,ab,kw. (13211)

38 (decision making adj2 (system* or tool*)).ti,ab,kw. (3662)

39 Expert System/ (5507)

40 (expert adj2 system*).ti,ab,kw. (5205)

41 Reminder System/ (2730)

42 ((computer* or electronic* or CDSS) adj2 (reminder* or alert*)).ti,ab,kw. (1848)

43 ((medication or medicine or treatment or therapy) adj2 (reminder* or alert*)).ti,ab. (1362)

44 reminder system*.ti,ab,kw. (1189)

45 physician order entry system/ (284)

46 ((computer* or electronic*) adj2 order entry).ti,ab,kw. (2801)

47 CPOE.ti,ab,kw. (1715)

48 (computer* adj2 decision support*).ti,ab,kw. (1907)

49 or/33-48 (56905)

50 32 or 49 [All computerised clinical decision support systems terms] (106747)

51 Occupation/ (52894)

52 physiotherapist assistant/ (83)

53 physiotherapist/ (23150)

54 speech disorder/ (27422)

55 Occupational Therapy/ (25731)

56 dietitian/ (13219)

57 Anesthesiologist/ (7231)

58 osteopathic physician/ (356)

59 radiographer/ (634)

60 podiatrist/ (831)

61 anesthesiologist*.ti,ab,kw. (34979)

62 podiatrist*.ti,ab,kw. (1315)

63 prosthetist*.ti,ab,kw. (635)

64 chiropodist*.ti,ab,kw. (179)

65 orthoptist*.ti,ab,kw. (620)

66 orthotist*.ti,ab,kw. (419)

67 osteopath*.ti,ab,kw. (8365)

68 radiographer*.ti,ab,kw. (4001)

69 art therapist*.ti,ab,kw. (266)

70 drama therapist*.ti,ab,kw. (20)

71 music therapist*.ti,ab,kw. (607)

72 (allied adj2 health adj2 (profession* or worker* or personnel or occupation* or staff)).ti,ab,kw. (5338)

73 ((physical or physio* or occupational or language or speech) adj2 therap*).ti,ab,kw. (77705)

74 physiotherapist*.ti,ab,kw. (18271)

75 dietetic*.ti,ab,kw. (14409)

76 dietitian*.ti,ab,kw. (10785)

77 nutritionist*.ti,ab,kw. (5156)

78 Patient care/ (310700)

79 multi-disciplinary team/ (10246)

80 collaborative care team/ (903)

81 ((multidisciplinary or multi-disciplinary or multiprofessional or multi-professional or interdisciplinary or interprofessional) adj2 team*).ti,ab,kw. (57679)

82 rescue personnel/ (8059)

83 emergency health service/ (105109)

84 ambulance/ (14751)

85 air medical transport/ (2965)

86 paramedical personnel/ (14896)

87 paramedic*.ti,ab,kw. (13029)

88 HEMS.ti,ab,kw. (1067)

89 ems.ti,ab,kw. (19120)

90 emt.ti,ab,kw. (36500)

91 prehospital.ti,ab,kw. (18282)

92 pre-hospital.ti,ab,kw. (8656)

93 first responder*.ti,ab,kw. (3260)

94 emergency medical technician*.ti,ab,kw. (1553)

95 emergency services.ti,ab,kw. (6114)

96 ambulance*.ti,ab,kw. (17409)

97 field triage.ti,ab,kw. (382)

98 out-of-hospital.ti,ab,kw. (19034)

99 (nurse or nurses or nursing).ti,ab,kw. (554357)

100 exp nurse/ (194823)

101 nursing staff/ (73869)

102 midwife/ (28233)

103 (midwif* or midwiv*).ti,ab. (29459)

104 or/51-103 [allied health professionals or nurses or midwives] (1389786)

105 50 and 104 [all CDSS and allied health professionals or nurses or midwives] (16820)

3. PsycINFO 1806 to February 12,2021 Search Strategy:

1 exp Decision Making/ (124412)

2 Decision Support Systems/ (3377)

3 (decision* adj2 making).ti,ab. (93578)

4 (decision* adj2 support*).ti,ab. (5773)

5 (decision* adj2 aid*).ti,ab. (1934)

6 or/1-5 (168090)

7 exp Computers/ (43893)

8 exp information systems/ (48548)

9 exp information/ (44565)

10 Internet/ (29404)

11 computer software/ (10412)

12 mobile Phones/ (4735)

13 smartphones/ (1843)

14 mobile applications/ (1082)

15 Mobile devices/ (2634)

16 exp Telemedicine/ (9383)

17 Health Information Technology/ (304)

18 Electronic Health Records/ (880)

19 computer*.ti,ab. (91287)

20 electronic*.ti,ab. (33377)

21 (internet or web or online or on-line).ti,ab. (145714)

22 (software or computer program*).ti,ab. (31224)

23 (automate* or automation).ti,ab. (14470)

24 (pda or pdas).ti,ab. (937)

25 personal digital assistant*.ti,ab. (440)

26 (app or apps).ti,ab. (7624)

27 (application* adj2 mobile*).ti,ab. (1392)

28 (iPad* or iPhone* or mobile phone or smartphone* or smart phone* or smart device* or android phone* or cellphone* or cell phone*).ti,ab. (10036)

29 (tablet adj2 (pc or device* or comput*)).ti,ab. (680)

30 ((hand held or handheld) adj2 (pc or device* or comput*)).ti,ab. (813)

31 (telehealth or telecare or telemedicine or ehealth or mhealth).ti,ab. (4475)

32 or/7-31 (362180)

33 6 and 32 (21605)

34 Decision Support Systems/ (3377)

35 Computer Assisted Diagnosis/ (1589)

36 (computer assisted adj2 (decision* or diagnos* or therap* or support or treatment? or management)).ti,ab. (273)

37 (computer aided adj2 (decision* or diagnos* or therap* or support or treatment? or management)).ti,ab. (179)

38 (decision adj2 support adj2 (system* or tool*)).ti,ab. (2189)

39 (decision making adj2 (system* or tool*)).ti,ab. (1022)

40 Expert Systems/ (5732)

41 (expert adj2 system*).ti,ab. (1376)

42 ((medication or medicine or treatment or therapy) adj2 (reminder* or alert*)).ti,ab. (202)

43 reminder system*.ti,ab. (125)

44 ((computer* or electronic*) adj2 order entry).ti,ab. (94)

45 (computer* adj2 decision support*).ti,ab. (183)

46 CPOE.ti,ab. (46)

47 or/33-46 [CDSS terms] (30902)

48 Allied Health Personnel/ (1109)

49 Physical Therapists/ (536)

50 Physical Therapy/ (2987)

51 Speech therapists/ (1229)

52 Speech Language Pathology/ (1088)

53 Occupational Therapists/ (2346)

54 anesthesiologist*.ti,ab. (457)

55 podiatrist*.ti,ab. (47)

56 prosthetist*.ti,ab. (23)

57 orthoptist*.ti,ab. (17)

58 [chiropodist*.ti,kw.] (0)

59 [orthotist*.ti,kw.] (0)

60 [osteopath*.ti,kw.] (0)

61 radiographer*.ti,ab. (81)

62 art therapist*.ti,ab. (1375)

63 drama therapist*.ti,ab. (75)

64 music therapist*.ti,ab. (1337)

65 (allied adj2 health adj2 (profession* or worker* or personnel or occupation* or staff)).ti,ab. (1123)

66 ((physical or physio* or occupational or language or speech) adj2 therap*).ti,ab. (18118)

67 physiotherapist*.ti,ab. (1346)

68 dietetic*.ti,ab. (610)

69 dietitian*.ti,ab. (756)

70 nutritionist*.ti,ab. (417)

71 Interdisciplinary Treatment Approach/ (7399)

72 ((multidisciplinary or multi-disciplinary or multiprofessional or multi-professional or interdisciplinary or interprofessional) adj2 team*).ti,ab. (8106)

73 emergency services/ (8779)

74 emergency personnel/ (117)

75 paramedics/ (337)

76 HEMS.ti,ab. (27)

77 ems.ti,ab. (1010)

78 emt.ti,ab. (230)

79 prehospital.ti,ab. (387)

80 pre-hospital.ti,ab. (262)

81 first responders/ (307)

82 emergency medical technician*.ti,ab. (154)

83 emergency services.ti,ab. (1211)

84 ambulance*.ti,ab. (860)

85 field triage.ti,ab. (6)

86 out-of-hospital.ti,ab. (355)

87 exp nurses/ (32673)

88 nursing/ (23241)

89 (nurse or nurses or nursing).ti,ab. (97190)

90 midwifery/ (1436)

91 (midwif* or midwiv*).ti,ab. (3137)

92 or/48-91 [allied health professionals or nurses or midwives] (148809)

93 47 and 92 [all CDSS and allied health professionals or nurses or midwives] (1171)

4. **Database: HMIC Health Management Information Consortium 1983 – February 12, 2021**
Search Strategy:

1 exp Decision Making/ (5606)

2 (decision* adj2 making).ti,ab. (6795)

3 (decision* adj2 support*).ti,ab. (871)

4 (decision* adj2 aid*).ti,ab. (276)

5 or/1-4 (10211)

6 exp Computers/ (2133)

7 exp information systems/ (4916)

8 exp medical Informatics/ (67)

9 Internet/ (1342)

10 Software/ (0)

11 telephone/ (110)

12 Telemedicine/ (1328)

13 computerised medical records systems.ti,ab. (0)

14 Medical Records/ (1946)

15 computer*.ti,ab. (6305)

16 electronic*.ti,ab. (4484)

17 (internet or web or online or on-line).ti,ab. (5066)

18 (software or computer program*).ti,ab. (1593)

19 (automate* or automation).ti,ab. (605)

20 (pda or pdas).ti,ab. (56)

21 personal digital assistant*.ti,ab. (32)

22 (app or apps).ti,ab. (130)

23 (application* adj2 mobile*).ti,ab. (32)

24 (iPad* or iPhone* or smartphone* or smart phone* or smart device* or android phone* or cellphone* or cell phone*).ti,ab. (146)

25 (tablet adj2 (pc or device* or comput*)).ti,ab. (16)

26 ((hand held or handheld) adj2 (pc or device* or comput*)).ti,ab. (61)

27 (telehealth or telecare or telemedicine or mhealth or ehealth).ti,ab. (1453)

28 or/6-27 (22729)

29 5 and 28 (1239)

30 (computer assisted adj2 (decision* or diagnos* or therap* or support or treatment? or management)).ti,ab. (25)

31 (computer aided adj2 (decision* or diagnos* or therap* or support or treatment? or management)).ti,ab. (17)

32 (decision adj2 support adj2 (system* or tool*)).ti,ab. (347)

33 (decision making adj2 (system* or tool*)).ti,ab. (107)

34 Expert Systems/ (107)

35 (expert adj2 system*).ti,ab. (131)

36 ((computer* or electronic* or CDSS) adj2 (reminder* or alert*)).ti,ab. (48)

37 reminder system*.ti,ab. (44)

38 ((computer* or electronic* or CDSS) adj2 (reminder* or alert*)).ti,ab. (48)

39 ((computer* or electronic*) adj2 order entry).ti,ab. (58)

40 (computer* adj2 decision support*).ti,ab. (114)

41 CPOE.ti,ab. (26)

42 or/29-41 [all CDSS terms] (1714)

43 Allied Health Personnel/ (0)

44 Physical Therapy Speciality/ (0)

45 Physiotherapists/ (350)

46 Speech-Language Pathology/ (0)

47 Occupational Therapists/ (542)

48 podiatrists/ (59)

49 anesthesiologist*.ti,ab. (11)

50 podiatrist*.ti,ab. (37)

51 prosthetist*.ti,ab. (19)

52 chiropodist*.ti,ab. (76)

53 orthoptist*.ti,ab. (23)

54 orthotist*.ti,ab. (15)

55 osteopath*.ti,ab. (93)

56 radiographer*.ti,ab. (178)

57 art therapist*.ti,ab. (5)

58 drama therapist*.ti,ab. (2)

59 music therapist*.ti,tw. (15)

60 (allied adj2 health adj2 (profession* or worker* or personnel or occupation* or staff)).ti,ab. (368)

61 ((physical or physio* or occupational or language or speech) adj2 therap*).ti,ab. (2010)

62 physiotherapist*.ti,ab. (671)

63 dietetic*.ti,ab. (187)

64 dietitian*.ti,ab. (130)

65 nutritionist*.ti,ab. (28)

66 Patient care team/ (139)

67 ((multidisciplinary or multi-disciplinary or multiprofessional or multi-professional or interdisciplinary or interprofessional) adj2 team*).ti,ab. (1676)

68 exp emergency medical services/ (0)

69 paramedic*.ti,ab. (395)

70 HEMS.ti,ab. (11)

71 ems.ti,ab. (51)

72 emt.ti,ab. (3)

73 prehospital.ti,ab. (58)

74 pre-hospital.ti,ab. (137)

75 first responder*.ti,ab. (28)

76 emergency medical technician*.ti,ab. (8)

77 emergency services.ti,ab. (514)

78 ambulance*.ti,ab. (1710)

79 field triage.ti,ab. (1)

80 out-of-hospital.tw. (292)

81 nurses/ (12920)

82 nursing staff/ (12920)

83 (nurse or nurses or nursing).ti,ab. (39541)

84 midwifery/ (665)

85 (midwif* or midwiv*).ti,ab. (4553)

86 or/43-85 [allied health professionals or nurses or midwives] (50288)

87 42 and 86 [all CDSS terms and allied health professionals or nurses or midwives] (291)

5. AMED (Allied and Complementary Medicine) 1985 to October 2019Search Strategy:

1 exp Decision Making/ (4522)

2 (decision* adj2 making).ti,ab. (2826)

3 (decision* adj2 support*).ti,ab. (217)

4 (decision* adj2 aid*).ti,ab. (92)

5 or/1-4 (6218)

6 exp Computers/ (1765)

7 exp information systems/ (150)

8 exp medical Informatics/ (775)

9 Internet/ (1242)

10 Software/ (450)

11 telephone/ (377)

12 Telemedicine/ (985)

13 computerised medical records systems.ti,ab. (0)

14 Medical Records/ (383)

15 computer*.ti,ab. (4200)

16 electronic*.ti,ab. (2339)

17 (internet or web or online or on-line).ti,ab. (6503)

18 (software or computer program*).ti,ab. (1436)

19 (automate* or automation).ti,ab. (399)

20 (pda or pdas).ti,ab. (77)

21 personal digital assistant*.ti,ab. (26)

22 (app or apps).ti,ab. (175)

23 (application* adj2 mobile*).ti,ab. (39)

24 (iPad* or iPhone* or smartphone* or smart phone* or smart device* or android phone* or cellphone* or cell phone*).ti,ab. (225)

25 (tablet adj2 (pc or device* or comput*)).ti,ab. (29)

26 ((hand held or handheld) adj2 (pc or device* or comput*)).ti,ab. (40)

27 (telehealth or telecare or telemedicine or mhealth or ehealth).ti,ab. (555)

28 or/6-27 (16500)

29 5 and 28 (443)

30 (computer assisted adj2 (decision* or diagnos* or therap* or support or treatment? or management)).ti,ab. (18)

31 (computer aided adj2 (decision* or diagnos* or therap* or support or treatment? or management)).ti,ab. (13)

32 (decision adj2 support adj2 (system* or tool*)).ti,ab. (41)

33 (decision making adj2 (system* or tool*)).ti,ab. (62)

34 Expert Systems/ (12)

35 (expert adj2 system*).ti,ab. (46)

36 ((computer* or electronic* or CDSS) adj2 (reminder* or alert*)).ti,ab. (7)

37 reminder system*.ti,ab. (3)

38 ((computer* or electronic* or CDSS) adj2 (reminder* or alert*)).ti,ab. (7)

39 ((computer* or electronic*) adj2 order entry).ti,ab. (0)

40 (computer* adj2 decision support*).ti,ab. (8)

41 CPOE.ti,ab. (0)

42 or/29-41 [all CDSS terms] (593)

43 Allied Health Personnel/ (659)

44 Physical Therapy Speciality/ (2201)

45 Physiotherapists/ (1476)

46 Speech-Language Pathology/ (237)

47 Occupational Therapists/ (1076)

48 podiatrists/ (36)

49 anesthesiologist*.ti,ab. (64)

50 podiatrist*.ti,ab. (172)

51 prosthetist*.ti,ab. (84)

52 chiropodist*.ti,ab. (32)

53 orthoptist*.ti,ab. (1)

54 orthotist*.ti,ab. (63)

55 osteopath*.ti,ab. (1733)

56 radiographer*.ti,ab. (18)

57 art therapist*.ti,ab. (179)

58 drama therapist*.ti,ab. (10)

59 music therapist*.ti,tw. (115)

60 (allied adj2 health adj2 (profession* or worker* or personnel or occupation* or staff)).ti,ab. (285)

61 ((physical or physio* or occupational or language or speech) adj2 therap*).ti,ab. (14459)

62 physiotherapist*.ti,ab. (2897)

63 dietetic*.ti,ab. (133)

64 dietitian*.ti,ab. (74)

65 nutritionist*.ti,ab. (39)

66 Patient care team/ (1786)

67 ((multidisciplinary or multi-disciplinary or multiprofessional or multi-professional or interdisciplinary or interprofessional) adj2 team*).ti,ab. (1129)

68 exp emergency medical services/ (420)

69 paramedic*.ti,ab. (78)

70 HEMS.ti,ab. (1)

71 ems.ti,ab. (96)

72 emt.ti,ab. (65)

73 prehospital.ti,ab. (32)

74 pre-hospital.ti,ab. (13)

75 first responder*.ti,ab. (9)

76 emergency medical technician*.ti,ab. (8)

77 emergency services.ti,ab. (24)

78 ambulance*.ti,ab. (45)

79 field triage.ti,ab. (0)

80 out-of-hospital.tw. (10429)

81 nurses/ (1071)

82 nursing staff/ (213)

83 (nurse or nurses or nursing).ti,ab. (9441)

84 midwifery/ (120)

85 (midwif* or midwiv*).ti,ab. (239)

86 or/43-85 [allied health professionals or nurses or midwives] (41793)

87 42 and 86 [all CDSS terms and allied health professionals or nurses or midwives] (186)

6. CINAHL EBSCO Search Strategy

#	Query*	Results
S101	S46 AND S100	11,824
S100	S47 OR S48 OR S49 OR S50 OR S51 OR S52 OR S53 OR S54 OR S55 OR S56 OR S57 OR S58 OR S59 OR S60 OR S61 OR S62 OR S63 OR S64 OR S65 OR S66 OR S67 OR S68 OR S69 OR S70 OR S71 OR S72 OR S73 OR S74 OR S75 OR S76 OR S77 OR S78 OR S79 OR S80 OR S81 OR S82 OR S83 OR S84 OR S85 OR S86 OR S87 OR S88 OR S89 OR S90 OR S91 OR S92 OR S93 OR S94 OR S95 OR S96 OR S97 OR S98 OR S99	867,856
S99	TI ((midwif* or midwiv*)) OR AB ((midwif* or midwiv*))	35,031
S98	(MH "Midwives+")	15,748
S97	(MH "Midwifery+")	20,976
S96	TI (((nurse or nurses or nursing)) OR ((nurse or nurses or nursing))) OR AB (((nurse or nurses or nursing)) OR ((nurse or nurses or nursing)))	535,366
S95	(MH "Nursing Staff, Hospital") "	20,953
S94	(MH "Nurses+")	228,583
S93	TI "music therapist*" OR AB "music therapist*"	592
S92	TI "drama therapist*" OR AB "drama therapist*"	6
S91	TI "art therapist*" OR AB "art therapist*"	420
S90	TI radiographer* OR AB radiographer*	2,300
S89	TI osteopath* OR AB osteopath*	3,074
S88	TI orthotist* OR AB orthotist*	188
S87	TI orthoptist* OR AB orthoptist*	34
S86	TI chiropodist* OR AB chiropodist*	458
S85	TI prosthetist* OR AB prosthetist*	335
S84	TI podiatrist* OR AB podiatrist*	2,440
S83	TI anesthesiologist* OR AB anesthesiologist*	6,441
S82	(MH "Radiologic Technologists")	5,733
S81	(MH "Osteopaths")	682
S80	(MH "Podiatrists")	2,444
S79	MH "Anesthesiologists")	1,495

S78	TI "out-of-hospital" OR AB "out-of-hospital"	6,634
S77	TI "field triage" OR AB "field triage"	173
S76	TI ambulance* OR AB ambulance*	6,499
S75	TI "emergency services" OR AB "emergency services"	1,921
S74	TI "emergency medical technician*" OR AB "emergency medical technician*"	725
S73	"first responder*" OR AB "first responder*"	1,402
S72	TI pre-hospital OR AB pre-hospital	2,500
S71	TI prehospital OR AB prehospital	7,480
S70	TI emt OR AB emt	2,753
S69	TI EMS OR AB EMS	9,336
S68	TI HEMS OR AB HEMS	1,348
S67	TI paramedic* OR AB paramedic*	5,903
S66	(MH "Ambulances")	4,565
S65	(MH "Emergency Medical Services")	26,747
S64	(MH "Emergency Medical Technicians")	12,426
S63	TI (((multidisciplinary or multi-disciplinary or multiprofessional or "multi-professional" or interdisciplinary or interprofessional)) OR AB ((multidisciplinary or "multi-disciplinary" or multiprofessional or "multi-professional" or interdisciplinary or interprofessional) N2 team*))	33,294
S62	(MH "Multidisciplinary Care Team")	45,878
S61	TI nutritionist* OR AB nutritionist*	1,676
S60	TI dietitian* OR AB dietitian*	5,004
S59	TI physiotherapist* OR AB physiotherapist*	8,379
S58	TI (((physical or occupational or language or speech) N1 therapist*)) AND AB (((physical or occupational or language or speech) N1 therapist*))	2,999
S57	TI ((allied N2 health N2 (profession* or worker* or personnel or occupation* or staff))) OR AB ((allied N2 health N2 (profession* or worker* or personnel or occupation* or staff)))	2,748
S56	(MH "Dietetics")	2,356

S55	(MH "Nutrition Services")	1,054
S54	(MH "Occupational Therapy")	23,116
S53	(MH "Speech-Language Pathology")	6,105
S52	(MH "Physical Therapists")	12,660
S51	(MH "Physical Therapy")	35,365
S50	(MH "Physical Therapist Assistants")	814
S49	TI "music therapist*" OR AB "music therapist*"	592
S48	TI "Physical Therapist Assistant*" or AB "Physical Therapist Assistant*"	276
S47	(MH "Allied Health Personnel")	4,326
S46	S31 or S32 or S33 or S34 or S35 or S36 or S37 or S38 or S39 or S40 or S41 or S42 or S43 or S44 or S45 or S46	94,625
S45	TI (((computer* or electronic*) N2 order entry)) OR AB (((computer* or electronic*) N2 order entry)) or TI ((CPOE or computer* N2 decision*)) or AB ((CPOE or computer* N2 decision*)	2,368
S44	(MH "Electronic Order Entry")	3,355
S43	TI "reminder system*" OR AB "reminder system*"	390
S42	TI (((computer* or electronic* or CDSS) N2 (reminder* or alert*))) OR AB (((computer* or electronic* or CDSS) N2 (reminder* or alert*))) or TI ((medication or medicine or treatment or therapy) N2 (reminder* or alert*)) or AB ((medication or medicine or treatment or therapy) N2 (reminder* or alert*))	1,691
S41	(MH "Reminder Systems")	2,949
S40	TI (expert N2 system*) OR AB (expert N2 system*)	1,008
S39	(MH "Expert Systems")	524
S38	TI ((decision making N2 (system* or tool*))) OR AB ((decision making N2 (system* or tool*)))	1,643
S37	TI ((decision N2 support N2 (system* or tool*))) OR AB ((decision N2 support N2 (system* or tool*)))	3,935
S36	TI ((("computer aided" N2 (decision* or diagnos* or therap*))) OR AB ((("computer aided" N2 (decision* or diagnos* or therap*)))	712
S35	TI ((("computer aided" adj2 (decision* or diagnos* or therap* or support or treatment* or management))) OR AB	9

	((("computer aided" adj2 (decision* or diagnos* or therap* or support or treatment* or management))	
S34	TI ((("computer assisted" N2 (decision* or diagnos* or therap* or support or treatment* or management))) OR AB ((("computer assisted" N2 (decision* or diagnos* or therap* or or support or treatment* or management)))	309
S33	(MH "Decision Support Systems, Clinical")	5,533
S32	(MH "Decision Making, Computer Assisted+")	45,289
S31	S6 AND S30	41,561
S30	S7 OR S8 OR S9 OR S10 OR S11 OR S12 OR S13 OR S14 OR S15 OR S16 OR S17 OR S18 OR S19 OR S20 OR S21 OR S22 OR S23 OR S24 OR S25 OR S26 OR S27 OR S28 OR S29	1,131, 998
S29	TI ((telehealth or telecare or telemedicine OR mhealth or ehealth)) OR AB ((telehealth or telecare or telemedicine or mhealth or ehealth))	14,130
S28	TI ((tablet N2 (pc or device* or comput*))) OR AB ((tablet N2 (pc or device* or comput*))) or TI ((handheld or "hand held" N2 (pc or device* or comput*)) or AB ((handheld or "hand held" N2 (pc or device* or comput*))	3,837
S27	TI ((iPad* or iPhone* or smartphone* or "smart phone*" or "smart device*" or "mobile phone*" or "android phone*" or cellphone* or "cell phone*")) OR AB ((iPad* or iPhone* or smartphone* or "smart phone*" or "smart device*" or "mobile phone* or "android phone*" or cellphone* or "cell phone*"))	11,037
S26	TI (application* N2 mobile*) OR AB (application* N2 mobile*)	2,919
S25	TI ((app or apps)) OR AB ((app or apps))	10,043
S24	TI "personal digital assistant*" OR AB "personal digital assistant*"	638
S23	TI ((pda or pdas)) OR AB ((pda or pdas))	2,146
S22	TI (automate* or automation) OR AB (automate* or automation)	22,986
S21	TI ((software or "computer program*")) OR AB ((software or "computer program*"))	50,295
S20	TI ((internet or web or online or on-line)) OR AB ((internet or web or online or on-line))	244,18 9
S19	TI electronic* OR AB electronic*	78,890

S18	TI computer* AND AB computer*	9,388
S17	(MH "Electronic Health Records+")	26,300
S16	(MH "Patient Record Systems+")	34,339
S15	(MH "Telemedicine+")	15,487
S14	(MH "Mobile Applications")	8,506
S13	(MH "Smartphone")	2,987
S12	(MH "Cellular Phone")	1,971
S11	(MH "Software")	29,588
S10	(MH "Internet")	50,622
S9	(MH "Informatics+")	899,135
S8	(MH "Information Systems+")	197,429
S7	(MH "Computers and Computerization+")	746,390
S6	S1 OR S2 OR S3 OR S4 OR S5	173,388
S5	TI (decision* N2 aid*) OR AB (decision* N2 aid*)	3,509
S4	TI (decision* N2 support*) OR AB (decision* N2 support*)	11,135
S3	TI (decision* N2 making) OR AB (decision* N2 making)	68,249
S2	(MH "Decision Support Techniques")	6,986
S1	(MH "Decision Making+")	111,200
*, Interface - EBSCOhost Research Databases, Search Screen - Advanced Search, Database - CINAHL , Limiters/Expanders: Search modes - Boolean/Phrase		

7. Cochrane Library search strategy

#1 MeSH descriptor: [Decision Making] explode all trees 3960

#2 MeSH descriptor: [Decision Support Techniques] explode all trees 2466

#3 (decision* near/2 making):ti,ab,kw (Word variations have been searched) 14369

#4 ((decision* near/2 support*)):ti,ab,kw (Word variations have been searched) 3552

#5 (decision* near/2 aid*):ti,ab,kw (Word variations have been searched) 1657

#6 {or #1-#5} 20279

#7 MeSH descriptor: [Computers] explode all trees 1732

#8 MeSH descriptor: [Information Systems] explode all trees 2293

#9 MeSH descriptor: [Informatics] explode all trees 8936

#10 MeSH descriptor: [Patient Portals] this term only 19

#11 MeSH descriptor: [Software] this term only 940

#12 MeSH descriptor: [Mobile Applications] this term only 686

#13 MeSH descriptor: [Cell Phone] explode all trees 1710

#14 MeSH descriptor: [Telemedicine] explode all trees 2649

#15 MeSH descriptor: [Medical Records Systems, Computerized] this term only 196

#16 MeSH descriptor: [Electronic Health Records] 1 tree(s) exploded 359

#17 (computer*):ti,ab,kw (Word variations have been searched) 47867

#18 (electronic*):ti,ab,kw (Word variations have been searched) 17343

#19 (internet or web or online or on-line):ti,ab,kw (Word variations have been searched) 32321

#20 (software or "computer program*"):ti,ab,kw (Word variations have been searched) 24140

#21 (automate* or automation):ti,ab,kw (Word variations have been searched) 8858

#22 (pda or pdas):ti,ab,kw (Word variations have been searched) 1067

#23 ("personal digital assistant*"):ti,ab,kw (Word variations have been searched) 168

#24 ((app or apps)):ti,ab,kw (Word variations have been searched) 4858

#25 (application* near/2 mobile*):ti,ab,kw (Word variations have been searched) 2489

#26 ((iPad* or iPhone* or smartphone* or "smart phone*" or "smart device*" or "android phone" or "cellphone*" or "cell phone*")):ti,ab,kw (Word variations have been searched) 6453

#27 ((tablet near/2 (pc or device* or comput*))):ti,ab,kw (Word variations have been searched) 936

#28 (("hand held" or handheld) near/2 (pc or device* or comput*)):ti,ab,kw 720

#29 ((telehealth or telecare or telemedicine or eHealth or mHealth)):ti,ab,kw (Word variations have been searched) 6874

#30 {or #7-#29} 124876

#31 #6 and #30 7180

#32 MeSH descriptor: [Decision Making, Computer-Assisted] explode all trees 4237

#33 MeSH descriptor: [Decision Support Systems, Clinical] this term only 380

#34 ((computer assisted near/2 (decision* or diagnos* or therap* or support or treatment* or management))):ti,ab,kw (Word variations have been searched) 2996

#35 ((computer aided near/2 (decision* or diagnos* or therap* or support or treatment* or management))):ti,ab,kw (Word variations have been searched) 191

#36 ((decision near/2 support near/2 (system* or tool*))):ti,ab,kw (Word variations have been searched) 1893

#37 ((decision making near/2 (system* or tool*))):ti,ab,kw (Word variations have been searched) 241

#38 MeSH descriptor: [Expert Systems] this term only 58

#39 ((expert near/2 system*)):ti,ab,kw (Word variations have been searched) 243

#40 MeSH descriptor: [Reminder Systems] this term only 953

#41 (((computer* or electronic*) near/2 (reminder* or alert*))):ti,ab,kw (Word variations have been searched) 445

#42 (reminder system*):ti,ab,kw (Word variations have been searched) 2798

#43 ((medication or medicine or treatment or therapy) near/2 (reminder* or alert)):ti,ab,kw 339

#44 MeSH descriptor: [Medical Order Entry Systems] this term only 67

#45 (((computer* or electronic*) near/2 order entry)):ti,ab,kw (Word variations have been searched) 119

#46 (computer* near/2 "decision support*") 476

#47 {or #32-#46} 10556

#48 #31 or #47 15798

#49 MeSH descriptor: [Allied Health Personnel] this term only 273

#50 MeSH descriptor: [Allied Health Occupations] this term only 7

#51 MeSH descriptor: [Physical Therapist Assistants] this term only 2

#52 MeSH descriptor: [Physical Therapy Specialty] this term only 120

#53 MeSH descriptor: [Speech-Language Pathology] this term only 67

#54 MeSH descriptor: [Occupational Therapy] this term only 775

#55 MeSH descriptor: [Nutritionists] this term only 44

#56 MeSH descriptor: [Dietetics] this term only 96

#57 MeSH descriptor: [Anesthesiologists] this term only 36

#58 MeSH descriptor: [Podiatry] this term only 39

#59 MeSH descriptor: [Osteopathic Physicians] this term only 3

#60 (anesthesiologist*):ti,ab,kw 7826

#61 (podiatrist*):ti,ab,kw 116

#62 (prosthetist*):ti,ab,kw 35

#63 (chiropodist*):ti,ab,kw 10

#64 (orthoptist*):ti,ab,kw 43

#65 (orthotist*):ti,ab,kw 32

#66 (osteopath*):ti,ab,kw 753

#67 (radiographer*):ti,ab,kw 132

#68 ("art therapist*"):ti,ab,kw 12

#69 ("music therapist*"):ti,ab,kw 137

#70 (" drama therapist*"):ti,ab,kw 2

#71 ((allied near/2 health near/2 (profession* or worker* or personnel or occupation* or staff))):ti,ab,kw (Word variations have been searched) 472

#72 (((physical or occupational or language or speech) near/ therapist*)):ti,ab,kw (Word variations have been searched) 31090

#73 (physiotherapist*):ti,ab,kw (Word variations have been searched) 5252

#74 (dietitian*):ti,ab,kw (Word variations have been searched) 2027

#75 (nutritionist*):ti,ab,kw (Word variations have been searched) 715

#76 MeSH descriptor: [Patient Care Team] this term only 1700

#77 (((multidisciplinary or "multi-disciplinary" or interdisciplinary or multiprofessional or "multi-professional" or interprofessional) near/2 team*)):ti,ab,kw (Word variations have been searched) 2422

#78 MeSH descriptor: [Emergency Medical Technicians] this term only 171

#79 MeSH descriptor: [Emergency Medical Services] this term only 1009

#80 MeSH descriptor: [Air Ambulances] this term only 41

#81 (paramedic*):ti,ab,kw (Word variations have been searched) 1181

#82 (HEMS):ti,ab,kw (Word variations have been searched) 242

#83 (ems):ti,ab,kw (Word variations have been searched) 2707

#84 (emt):ti,ab,kw (Word variations have been searched) 294

#85 (prehospital):ti,ab,kw (Word variations have been searched) 1778

#86 (pre-hospital):ti,ab,kw (Word variations have been searched) 672

#87 ("first responder*"):ti,ab,kw (Word variations have been searched) 147

#88 ("emergency medical technician*"):ti,ab,kw (Word variations have been searched) 277

#89 ("emergency services"):ti,ab,kw (Word variations have been searched) 2743

#90 (ambulance*):ti,ab,kw (Word variations have been searched) 989

#91 ("field triage"):ti,ab,kw (Word variations have been searched) 6

#92 ("out-of-hospital"):ti,ab,kw (Word variations have been searched) 1776

#93 MeSH descriptor: [Nursing] explode all trees 3292

#94 MeSH descriptor: [Nursing Care] explode all trees 1788

#95 MeSH descriptor: [Nursing Staff] explode all trees 648

#96 (nurse or nurses or nursing):ti,ab,kw (Word variations have been searched) 41946

#97 MeSH descriptor: [Midwifery] this term only 329

#98 (midwif* or midwiv*):ti,ab,kw (Word variations have been searched) 2309

#99 {or #49-#98} 99097

#100 #48 AND #99 2266

Cochrane Database of Systematic Reviews = 58 Cochrane Trials =2205

8. Social Science Citation Index Search Strategy

#	Search terms	Results

#7	#6 AND #5	2,297
#6	TS=(((("allied health" NEAR/2 (profession* OR worker* OR personnel OR occupation* OR staff)) OR (("physical therapist" OR "physical therapists") OR ("occupational therapist" OR "occupational therapists") OR ("language therapist" OR "language therapists") OR ("speech therapist" OR "speech therapists")) OR (physiotherapist* OR dietitian* OR dietetics OR nutritionist* or "music therapist*" or anesthesiologist* or orthoptist* or chiropodist* or podiatrist* or osteopath* or prosthetist* or orthotist* or radiographer* or "art therapist*" or "drama therapist*") OR ((multidisciplinary OR "multi-disciplinary" or interdisciplinary OR multiprofessional OR "multi-professional" or interprofessional) NEAR/2 team*) OR (nurse OR nurses OR nursing or paramedic* or HEMS or EMS or EMT or prehospital or "pre-hospital" or "first responder*" or "emergency medical technician*" or "emergency services" or ambulance* or "field triage" or "out-of-hospital" or midwif* or midwiv*))))	228,344
#5	#4 AND #3	34,209
#4	TS=(("computer assisted decision*" OR "computer assisted diagnos*" OR "computer assisted therap*") OR ("computer aided decision*" OR "computer aided diagnos*" OR "computer aided therap*" or "computer aided support" or "computer aided treatment*" or "computer aided management" or "computer assisted support" or "computer assisted treatment*" OR "computer assisted management") OR ("decision support system*" OR "decision support or tool*") OR ("decision making system*" OR "decision making tool*") OR (expert NEAR/2 system*) OR (computer* NEAR/2 reminder* OR computer NEAR/2 alert* OR electronic* NEAR/2 reminder* OR electronic* NEAR/2 alert*) OR "reminder system*" OR "medical Order Entry System*" OR (computer* NEAR/2 "order entry") OR (electronic* NEAR/2 "order entry") OR (computer* near/2 "decision making") OR (medication or medicine or treatment or therapy) Near/2 (reminder* or alert*))	13,896
#3	#2 AND #1	21,872
#2	TS=((((computer* OR electronic* OR internet OR web OR online OR on-line OR software OR computer program* OR automate* OR automation OR pda OR pdas OR "personal digital assistant*") OR (app OR apps OR application* NEAR/2 mobile* OR iPad* OR iPhone* OR smartphone* OR ("smart phone" OR "smart phones") OR ("smart device" OR "smart devices")) OR ("android phone*" or cellphone* or "cell phone*") OR (tablet NEAR/2 (pc OR device* OR comput*)) OR (telehealth OR telecare OR telemedicine or mhealth or ehealth))))	438,284

#1	((decision* near/2 making) OR TOPIC: (decision* near/2 support*) OR TOPIC: (decision* near/2 aid*))	190,122

9. Search Strategy Proquest ASSIA and Dissertations & Theses Abstracts & Index

ab(((((decision* NEAR/2 making) OR (decision* NEAR/2 support*) OR (decision* NEAR/2 aid*)) AND ((computer* OR electronic* OR internet OR web OR online OR on-line OR software OR computer program* OR automate* OR automation OR pda OR pdas OR "personal digital assistant*") OR (app OR apps OR application* NEAR/2 mobile* OR iPad* OR iPhone* OR smartphone* OR ("smart phone" OR "smart phones") OR ("smart device" OR "smart devices")) OR (tablet NEAR/2 (pc OR device* OR comput*)) OR (telehealth OR telecare OR telemedicine))) OR (("computer assisted decision*" OR "computer assisted diagnos*" OR "computer assisted therap*") OR ("computer aided decision*" OR "computer aided diagnos*" OR "computer aided therap*") OR ("decision support system*" OR "decision support or tool*") OR ("decision making system*" OR "decision making tool*") OR (expert NEAR/2 system*) OR (computer* NEAR/2 reminder* OR computer NEAR/2 alert* OR electronic* NEAR/2 reminder* OR electronic* NEAR/2 alert*) OR "reminder system*" OR "medical Order Entry System*" OR (computer* NEAR/2 "order entry" OR electronic* NEAR/2 "order entry"))) AND (("allied health" NEAR/2 (profession* OR worker* OR personnel OR occupation* OR staff)) OR (("physical therapist" OR "physical therapists") OR ("occupational therapist" OR "occupational therapists") OR ("language therapist" OR "language therapists") OR ("speech therapist" OR "speech therapists")) OR (physiotherapist* OR dietitian* OR dietetics OR nutritionist*) OR ((multidisciplinary OR interdisciplinary OR multiprofessional OR interprofessional) NEAR/2 team*) OR (nurse OR nurses OR nursing or paramedic* or HEMS or EMS or EMT or prehospital or "pre-hospital" or "first responder*" or "emergency medical technician*" or "emergency services" or ambulance* or "field triage" or "out-of-hospital" or midwif* or midwiv*))) OR ti(((((decision* NEAR/2 making) OR (decision* NEAR/2 support*) OR (decision* NEAR/2 aid*)) AND ((computer* OR electronic* OR internet OR web OR online OR on-line OR software OR computer program* OR automate* OR automation OR pda OR pdas OR "personal digital assistant*") OR (app OR apps OR application* NEAR/2 mobile* OR iPad* OR iPhone* OR smartphone* OR ("smart phone" OR "smart phones") OR ("smart device" OR "smart devices")) OR (tablet NEAR/2 (pc OR device* OR comput*)) OR (telehealth OR telecare OR telemedicine))) OR (("computer assisted decision*" OR "computer assisted diagnos*" OR "computer assisted therap*") OR ("computer aided decision*" OR "computer aided diagnos*" OR "computer aided therap*") OR ("decision support system*" OR "decision support or tool*") OR ("decision making system*" OR "decision making tool*") OR (expert NEAR/2 system*) OR (computer* NEAR/2 reminder* OR computer NEAR/2 alert* OR electronic* NEAR/2 reminder* OR electronic* NEAR/2 alert*) OR "reminder system*" OR "medical Order Entry System*" OR (computer* NEAR/2 "order entry" OR electronic* NEAR/2 "order entry"))) AND (("allied health" NEAR/2 (profession* OR worker* OR personnel OR occupation* OR staff)) OR (("physical therapist" OR "physical therapists") OR ("occupational therapist" OR "occupational therapists") OR ("language therapist" OR "language therapists") OR ("speech therapist" OR "speech therapists")) OR (physiotherapist* OR dietitian* OR dietetics OR nutritionist*) OR ((multidisciplinary OR interdisciplinary OR multiprofessional OR interprofessional) NEAR/2 team*) OR (nurse OR nurses OR nursing or paramedic* or HEMS or EMS or EMT or prehospital or "pre-hospital" or "first responder*" or "emergency medical technician*" or "emergency services" or ambulance* or "field triage" or "out-of-hospital" or midwif* or midwiv*)))

10. Search strategies -Clinicaltrials.gov, ICTRP, OpenGrey, OpenClinical, HealthIT.gov, Agency for Healthcare Research and Quality Health Information Technology website

Search 1: Decision* AND computer*

Search 2: Decision* AND web*

Search 3: Decision* AND online

Search 4: Decision* AND software

Search 5: Decision* AND device*

Search 6: Decision* AND mobile*

11. Search strategy Health Services Research Projects in Progress

(decision*) AND (computer* OR web* OR online OR software OR device* OR mobile* AND allied OR therapist* OR occupational OR therap* OR physiotherapist OR physiotherapy))

Supplementary Table 2: Risk of Bias assessment justifications using Effective Practice Organisation of Care (EPOC)'s tool

1. Randomised controlled trials, non-randomised trials and controlled before-after studies

Random sequence generation	Allocation concealment	Baseline outcome measurements similar	Baseline characteristics similar	Incomplete outcome data	Knowledge of the allocated interventions adequately prevented during the study	Protection against contamination	Selective outcome reporting	Other bias	Overall bias score
**Beeckman et al, 2013**									
"Simple randomisation was used to allocate nurses and patients"	Nurses and residents knew their allocated group	Reported baseline outcomes are broadly similar	Baseline characteristics balanced/similar	No information if there was a problem of missing data or ways of handling it, if any	Assessors were not blinded	Intervention was allocated nursing homes, not individual patients	All relevant outcomes in the methods section are reported in the results section	There is no evidence of other risk of biases	**High**
**Blaha et al, 2009**									
Not specified in paper.	Not specified in paper.	No significant differences in glucose at baseline	Although reported for patients, baseline characteristics of nurses is not reported in text or tables.	Only 11 of 120 patients missing (9%)	The outcomes are objective.	Professionals were allocated within a clinic or practice and it is possible that communication between the two groups could have occurred	All relevant outcomes in the methods section are reported in the results section.	There is no evidence of other risk of biases.	**Unclear**
**Byrne,2005**									
Controlled before-after study.	Controlled before-after study.	Models adjusted for covariates.	No report of baseline characteristics of patients or Nurses involved.	Not specified in the paper.	Not specified in the paper.	Unit of allocation was the nursing home	All relevant outcomes in the methods section are reported in the results section.	Multiple comparison	**High**
**Canbolat et al,2019 (NRCT)**									

Random sequence generation	Allocation concealment	Baseline outcome measurements similar	Baseline characteristics similar	Incomplete outcome data	Knowledge of the allocated interventions adequately prevented during the study	Protection against contamination	Selective outcome reporting	Other bias	Overall bias score
Is Non-randomised trial.	It is an open label study.	No baseline measure of outcomes reported.	No baseline information reported about the providers (Nurses); difference baseline characteristics patients present	Not specified in the paper.	Not specified in the paper.	There was no randomisation; control and intervention groups were from the same clinic. Therefore, it is highly likely that control group could have received intervention	All relevant outcomes are reported in the results section.	No baseline (pre-intervention) outcomes data available so difficult to judge.	**High**
**Cavalcanti et al, 209**									
'Random numbers were generated by computer.'	'Allocation was by centres at the start of the study.'	No baseline measure of outcomes reported in the paper.	Clinically significant differences in patients at baseline; no baseline information about HPs.	Outcomes reported were based on all participants (complete data).	Not specified in the paper.	Not specified in the paper.	All relevant outcomes in the methods section are reported in the results section.	No evidence of other sources of bias.	**High**
**Cleveringa et al,2008**									
Block randomisation by practices and Nurses.	Unit of allocation was by practice.	Baseline outcomes were largely similar among the intervention and control groups.	Clinically significant differences in patients at baseline; no baseline information about HPs.	'Values carried forward method' was used but not ideal method.	Not specified in the paper.	Allocation unit was practice so unlikely that the control group received an intervention.	All relevant outcomes discussed in the objective are reported.	No evidence of other risk of biases.	**High**

Random sequence generation	Allocation concealment	Baseline outcome measurements similar	Baseline characteristics similar	Incomplete outcome data	Knowledge of the allocated interventions adequately prevented during the study	Protection against contamination	Selective outcome reporting	Other bias	Overall bias score
**Cleveringa et al,2010**									
Not specified in the paper.	Unit of allocation was primary care practice.	Baseline outcome measurements are largely similar.	There is no report of baseline characteristics of Nurses in text or tables.	Use of electronic health records	Not specified in the paper.	Allocation was by primary care practices so unlikely that control group received intervention.	All relevant outcomes set out in the objective were reported.	No evidence of other risk of biases.	**High**
**Cortez, 2014**									
Not specified in the paper.	Allocation was based on clinic and nurses.	Outcome measurements were different among the two groups	Baseline characteristics were largely similar in both groups.	Use of electronic health records	'The study participants (nurses) did not know about the other group's usage of CDSS at the start and during the study.'	Nurses in the intervention group did not know about or receive CDSS during study.	All relevant outcomes in the methods section are reported in the results section.	No evidence of other risk of biases.	**High**
**Dalaba et al, 2015**									
A controlled before-after study.	A controlled before-after study.	Baseline outcome measurements were significantly different.	No report of baseline characteristics of HPs in text or tables	Not specified in the paper.	Not specified in the paper.	Comparison groups were in different districts.	All outcomes mentioned in the methods section have been reported.	No indication of other biases.	**High**
**Duclos et al,2015**									
Randomisation computer generated centrally.	Allocation was by department at the start of the study.	Baseline outcome measures appear to be	Only aggregated baseline characteristics of children for	Medical records were used.	Not specified in the paper.	Not specified in the paper.	All relevant outcomes in the methods section are reported in the results section.	No indication of other biases.	**High**

Random sequence generation	Allocation concealment	Baseline outcome measurements similar	Baseline characteristics similar	Incomplete outcome data	Knowledge of the allocated interventions adequately prevented during the study	Protection against contamination	Selective outcome reporting	Other bias	Overall bias score
		different and were not adjusted for during analysis.	the intervention and control groups; and, no report about the HP participants' baseline characteristics in tables or text.						
**Dumont et al,2012**									
Simple randomisation used	Randomisation was achieved by a Nurse choosing unmarked sealed envelope	No baseline measure of outcome reported.	Patient characteristics reported and largely similar, but report on HP were presented as aggregated.	Not specified in the paper.	Not specified in the paper.	Nurses were allocated within a clinic and it is possible that communication between intervention and control nurse could have occurred.	All outcomes in methods section were reported.	Performance bias risk from knowledge of cases, protocols and contamination highly likely.	**High**
**Dykes et al, 2009**									
Not specified in the paper	Allocation was by unit at the start of the study	Baseline outcome measurements are largely similar.	Patient characteristics were similar, but no information on HPs.	Medical records were used.	Study noted as open-label design in the protocol; and, intervention and control units in one hospital.	Contamination of information highly likely; patients rather than professionals were randomised	All outcomes in methods section were reported.	No indication of other biases.	**High**
**Fitzmaurice et al, 2000**									

Random sequence generation	Allocation concealment	Baseline outcome measurements similar	Baseline characteristics similar	Incomplete outcome data	Knowledge of the allocated interventions adequately prevented during the study	Protection against contamination	Selective outcome reporting	Other bias	Overall bias score
'Randomisation was computer generated.'	Not specified in the paper	Baseline outcome measurements are largely similar.	There is no report of baseline characteristics of HPs in text or tables	Use of medical records.	Outcomes are objective.	Groups in same practice—possibility of communication between health professionals	All relevant outcomes in the introduction/methods section are reported in the results section.	No evidence of other risk of biases.	**High**
**Forberg et al,2016**									
'A simple draw from the list by a third person.'	Not specified in the paper	Baseline measure of outcomes appear to be largely similar.	Baseline characteristics of the intervention and control groups are similar.	Missing outcomes is very minimal (<2%).	Not specified in the paper.	Not clear that nurses did not swap between units within the same hospital.	All relevant outcomes in the methods section are reported in the results section.	No evidence of other risk of biases.	**High**
**Fossum et al,2011**									
Controlled before-after study	Controlled before-after study	Baseline outcome measurements are largely similar.	Although reported for patients, baseline characteristics of providers was not reported in text or tables.	Use medical records.	Not specified in the paper.	Allocation was by nursing homes and is unlikely that control group received intervention.	All relevant outcomes in the methods section are reported in the results section.	No evidence of other risk of biases.	**High**
**Geurts et al, 2016**									
'Computer generated randomisation was used.'	'Centralised randomisation scheme used.'	No baseline measure of outcome in the paper.	Baseline characteristics are largely similar among the two groups.	Medical records used.	'Nurses were blinded for the contribution of predictors on the risk score.'	Patient based randomisation; a high possibility. Intra clinician and inter linician	All relevant outcomes in the methods section are reported in the results section.	Question about representativeness of final study sample as 75% of eligible kids not randomised as	**High**

Random sequence generation	Allocation concealment	Baseline outcome measurements similar	Baseline characteristics similar	Incomplete outcome data	Knowledge of the allocated interventions adequately prevented during the study	Protection against contamination	Selective outcome reporting	Other bias	Overall bias score
						contamination highly possible.		professional or parents non-compliant.	
**Hovorka et al, 2007**									
'randomisation based on computer algorithm'	Centralised randomisation scheme was used.	No baseline measure of outcome reported in the paper.	Although some report about patients, no report of baseline characteristics about HP participants in text or tables.	Not specified in the paper.	The outcomes were objective.	patients based randomisation; same clinicians involved in standard and intervention arms	All relevant outcomes in the methods section are reported in the results section.	No evidence of other risk of biases.	**High**
**Kroth et al, 2006**									
'Randomisation using coin flip.'	Not specified in the paper.	No baseline measure of outcome.	There is no detailed report of characteristics in text or tables.	Consecutive [medical] records used.	objective outcome	Randomisation was for patients and nurses. Nurses in the control group did not receive reminders.	All relevant outcomes in the methods section are reported in the results section.	No evidence of other risk of biases.	**High**
**Lattimer et al, 1998**									
'A random number generator pocket calculator (Hewlett Packard 21s) used'	Unit of allocation was by team and allocation was performed on all units at the start of the study.	No baseline measure of outcome reported.	Some about patients, but no report of baseline characteristics HPs in text or tables.	Not specified in the paper.	Use of medical records.	Health professionals in the intervention (Nurses) and control (Doctors) were different.	All relevant outcomes in the methods section are reported in the results section.	No evidence of other risk of biases.	**Unclear**

Random sequence generation	Allocation concealment	Baseline outcome measurements similar	Baseline characteristics similar	Incomplete outcome data	Knowledge of the allocated interventions adequately prevented during the study	Protection against contamination	Selective outcome reporting	Other bias	Overall bias score
**Lattimer et al,2000**									
Not specified in the paper.	Not specified in the paper.	Not specified in the paper.	There is no detailed report of characteristics in text or tables	Not specified in the paper.	Use of medical records.	Health professionals in the intervention (Nurses) and control (Doctors) were different.	All relevant outcomes in the methods section are reported in the results section.	No evidence of other risk of biases.	**Unclear**
**Lee et al, 2009**									
Not specified in the paper.	Not specified in the paper.	Although weight and BMI data were recorded, no data on the outcome measurements.	Reported for patients, but no report on providers in text or tables.	Not specified in the paper.	Not specified in the paper.	Patients based randomisation so it is likely that the control group received the intervention.	All relevant outcomes in the methods section are reported in the results section.	No evidence of other risk of biases.	**High**
**Lv et al, 2019**									
Not specified in the paper.	Not specified in the paper.	Not specified in the paper.	Reported for patients, but no report on providers in text or tables.	Not specified in the paper.	Not specified in the paper.	Patients based randomisation; Patient based randomisation; same clinicians involved in both arms.	All relevant outcomes in the methods section are reported in the results section.	No evidence of other risk of biases.	**High**
**Mann et al,2011**									
Computer generated sequence was used.	Not specified in the paper.	Baseline measure of outcome not reported.	No baseline characteristics of HPs in text or tables were found.	Not clear from the paper.	A cross-over study; not specified in the paper.	Acrossover trial with only patients rather than professionals randomised.	All relevant outcomes in the methods section are reported in the results section.	No evidence of other risk of biases.	**High**

Random sequence generation	Allocation concealment	Baseline outcome measurements similar	Baseline characteristics similar	Incomplete outcome data	Knowledge of the allocated interventions adequately prevented during the study	Protection against contamination	Selective outcome reporting	Other bias	Overall bias score
**McDonald et al, 2017**									
Automated block randomisation was used.	Automated block randomisation schema was used	Not specified in the paper.	Baseline characteristics were largely similar.	Possible medical records use.	Assessor was not blinded.	Both intervention and control nurses were in one organisation and it is possible that communication between them could have occurred	All relevant outcomes in the methods section are reported in the results section.	Only 42% of patients who should have had a CDSS applied suggesting that the nurses selectively chose which patients to use it with or selective non adoption	**High**
**Paulson et al, 2020**									
Automated block randomisation was used.	Automated block randomisation schema was used	Reported for patients, but no report on providers in text or tables	Baseline characteristics were largely similar	Only complete case analysis conducted	Outcomes are objective	Both intervention and control nurses were in one organisation and it is possible that communication between them could have occurred	All relevant outcomes in the methods section are reported in the results section.	No evidence of other risk of biases.	**High**
**Plank et al, 2006**									
Not specified in the paper	Not specified in the paper	Blood glucose measured but not intervention group based	Differences in types of surgery and history of diabetes between sites	Use of medical records.	Outcomes are objective.	same units delivering all arms of the trial with same clinicians	All relevant outcomes in the methods section are reported in the results section.	No evidence of other risk of biases.	**High**
**Rood et al, 2005**									

Random sequence generation	Allocation concealment	Baseline outcome measurements similar	Baseline characteristics similar	Incomplete outcome data	Knowledge of the allocated interventions adequately prevented during the study	Protection against contamination	Selective outcome reporting	Other bias	Overall bias score
'Automatic random number generating'	Not specified in the paper	Baseline measure of outcome not reported.	No report of characteristics of HPs in text or tables.	Not specified in the paper.	Not specified in the paper.	Patient based randomisation; same clinicians involved in both arms.	There is no evidence that outcomes were selectively reported.	No evidence of other risk of biases.	**High**
**Roukema et al,2008**									
Randomisation was based on computer algorithm.	'centralised randomisation scheme'	Baseline measure of outcome not reported	No report of characteristics of HPs in text or tables.	Not specified in the paper.	Not specified in the paper.	professionals were allocated within a clinic so hard to see how decision rule training effect not present in the clinicians who were delivering both arms of the trial	All relevant outcomes in the methods section are reported in the results section.	No evidence of other risk of biases.	**High**
**Sassen et al,2014**									
Not specified in the paper.	The unit of allocation was by health professional and allocation was performed on all units at the start of the study	No important differences were present across study groups.	Baseline characteristics of the study and control providers are reported and similar.	Significant proportion participants dropped out and the report is based on the complete case analysis.	Outcomes cannot be assessed blindly.	Participants in the control group did not have a log-in code to access the website (CDSS tool) until post-intervention data were collected.	All relevant outcomes in the methods section are reported in the results section.	No evidence of other risk of biases.	**High**
**Snooks et al, 2014**									

**Random sequence generation**	**Allocation concealment**	**Baseline outcome measurements similar**	**Baseline characteristics similar**	**Incomplete outcome data**	**Knowledge of the allocated interventions adequately prevented during the study**	**Protection against contamination**	**Selective outcome reporting**	**Other bias**	**Overall bias score**
Randomisation based on computer algorithm.	Random allocation was performed on all units at the start of the study.	No baseline measure of outcome reported.	No report of characteristics in text or tables about the paramedics involved.	Not specified in the paper.	Analyst was blinded.	Intervention and control groups were in separates sites	All relevant outcomes in the methods section are reported in the results section.	No evidence of other risk of biases.	**Unclear**
**Vadher et al, 1997**									
Random tables were used.	Not specified in the paper.	No baseline measure of outcome reported.	Patient baseline characteristics reported; one nurse versus a clinician.	Not specified in the paper.	Outcomes are objectively measured.	Hard to see how same clinicians seeing both arm trial patients didn't pick up something from the CDSS.	All relevant outcomes in the methods section are reported in the results section.	There was only one Nurse participant in the intervention group.	**High**
**Wells,2013**									
Random table was used for randomisation.	Not specified in the paper.	No baseline measure of outcomes reported.	Baseline characteristics are largely similar.	Not specified in the paper.	Outcomes were assessed blindly.	Intervention and control groups in the same site so it is likely that the control group received the intervention.	All relevant outcomes in the methods section are reported in the results section	No evidence of other risk of biases.	**High**

Colour codes: Red, high risk; orange, unclear risk; green, low risk

2. Interrupted time series studies

Author & Year	Risk of bias domains and scores							Overall bias
	**Intervention independent of other changes**	**Shape of the intervention effect pre-specified**	**Intervention unlikely to affect data collection**	**Knowledge of the allocated interventions adequately prevented during the study**	**Incomplete outcome data adequately**	**Selective outcome reporting**	**Other bias**	
Bennet, 2016	Very long adoption period with no measurement; possible confounding factors not presented/models not adjusted	Data were classified as pre and post-intervention from the point/date of intervention.	Data were collected from the hospital records databases for pre- and post-intervention periods	Not presented in the paper.	Medical records used	All relevant outcomes in the methods section are reported in the results section.	No evidence of other risk of biases.	**High**
Dykes et al,2020	Highly likely the changes in outcome to be influenced by confounders.	Point of analysis is the point of intervention.	Sources and methods of data collection were the same before and after the intervention.	Not presented in the paper.	Medical records used	All relevant outcomes are reported in the results section	No evidence of other risk of biases.	
Dowding et al,2012	Highly likely the changes in outcome to be influenced by confounders.	Point of analysis is the point of intervention.	Sources and methods of data collection were the same before and after the intervention.	Not presented in paper.	Medical records used	All relevant outcomes are reported in the results section.	No evidence of other risk of biases.	**High**

Colour codes: Red, high risk; orange, unclear risk; green, low risk

Supplementary Table 3: Summary of patient care process results

Author & Year	Interventions	Health professionals	patient participants	Outcome measured	Outcome values reported	Change of value within a group‡	Risk difference (95% CI)‡
**1. Adherence to guidelines**							
Dumont et al,2012	• CDSS use	Nurses (OA=44)	141 adults	Deviations from the protocol, out of 10 (mean (SD))	4 months=0.39(1.0)	-	Mean difference: -2.61 (-4.5 to -0.71)
	• Paper protocol	Nurses	159 adults		4 months=3.0(4.3)		
Forberg et al,2016	• CDSS-use	108 Nurses	Not applicable	Nurses adherence to guidelines on disinfection of hands	Baseline=97/108<br>3 months =93/105	-1.2%	6.7% (4.9 to 8.5)
	• CDSS non-use	103 Nurses	Not applicable		Baseline=96/103<br>3 months=87/102	-7.9%	
	• CDSS-use			Nurses adherence to guidelines on usage of disposable gloves (n/N)	Baseline=80/108<br>3 months =76/105	-1.7%	-1.4% (-2.2 to -0.5)
	• CDSS non-use				Baseline=71/103<br>3 months =70/102	-0.3%	
	• CDSS-use			Nurses adherence to guidelines on daily inspection of Peripheral Venous Catheters (PVC) site (n/N)	Baseline=58/108<br>3 months =58/103	2.6%	-5.2% (-7.1 to -3.3)
	• CDSS non-use				Baseline=47/102<br>3 months =55/102	7.8%	
Rood et al, 2005	• CDSS-based GL	ICU Nurses	66 adults	Adherence to Insulin dose Advice (n/N)	10 weeks =1818/2352	-	22% (19 to 25)
	• Paper-based GL	ICU Nurses	54 adults		10 weeks =1667/2597	-	
	• CDSS-based GL	ICU Nurses	66 adults	Adherence to the guideline for taking blood samples on time (n/N)	10 weeks =945/2352	-	4.7% (2.0 to 7.4)
	• Paper-based GL	ICU Nurses	54 adults		10 weeks =922/2597	-	
Vadher et al, 1997	• CDSS	1 Nurse	87 adults	Dose advice 'acceptance' in patients with therapeutic range 2-3	Post-test =188/214	-	28% (20.4 to 35.5)
	• Control	3 trainee Doctors	90 adults		Post-test=145/242	-	
	• CDSS	1 Nurse		Dose advice 'acceptance' in patients with therapeutic range 3-4.5 (n/N)	Post-test =160/239	-	-6.2% (-14.7 to 2.2)
	• Control	3 trainee Doctors			Post-test=150/205		
	• CDSS	1 Nurse		Interval advice 'acceptance' (%) in patients with therapeutic range 2-3	Post-test =170/230	-	23.9% (15.6 to 32.2)
	• Control	3 trainee Doctors			Post-test=133/266		
	• CDSS	1 Nurse		Interval advice 'acceptance' (%) in patients with therapeutic range 3-4.5	Post-test =129/239	-	3.9% (-5.4 to 13.3)
	• Control	3 trainee Doctors			Post-test=101/202		
**2. Patient assessment, diagnosis, and treatment practices**							
	• CDSS use period			Pain assessment	Post-test=97.7%	-	62.7% (59.6 to 65.8)

Bennett et al, 2016	• CDSS non use				Pre-test=35%		
	• CDSS use			IV antibiotics in 1hr for sepsis	Post-test=5.6%	-	-5.9% (-8.3 to -3.5)
	• CDSS non use				Pre-test=11.5%		
Duclos et al,2015	• CDSS	Dieticians	667 children	Investigation of malnutrition aetiology	Post-test=284/667	-	21.2% (15.9 to 26.5)
	• Usual care	Dieticians	477 children		Post-test=102/477		
	• CDSS	Dieticians	667 children	Managed by a dietitian	Post-test=305/667	-	12% (6.3 to 17.7)
	• Usual care	Dieticians	477 children		Post-test=161/477		
	• CDSS	Dieticians	667 children	prescribed refeeding protocol	Post-test=230/667	-	-4.5% (-10.2 to 1.2)
	• Usual care	Dieticians	477 children		Post-test=186/477		
Geurts et al, 2017	• CDSS	Nurses	113 children	Patient consultation time(min)-median (IQR)	Post-test =136(108)	-	3 min
	• Usual care	Nurses	109 children		Post-test =133(92)		
	• CDSS	Nurses	113 children	Electrolytes level test	Post-test =15/113	-	-7.8% (-17.7 to 2.1)
	• Usual care	Nurses	109 children		Post-test =23/109		
	• CDSS	Nurses	113 children	Acid-base balance test	Post-test =13/113	-	-3.2% (-12.1 to 5.7)
	• Usual care		109 children		Post-test =16/109		
	• CDSS	Nurses	113 children	Oral Rehydration Solution (nasogastric tube)	Post-test =17/113	-	6.7% (-1.6 to 15.2)
	• Usual care	Nurses	109 children		Post-test =9/109		
	• CDSS	Nurses	113 children	IV rehydration given	Post-test =0/113	-	-1.8% (-4.4 to 0.7)
	• Usual care	Nurses	109 children		Post-test =2/109		
	• CDSS	Nurses	113 children	Other liquid given	Post-test =18/113	-	-11.6% (-22.4 to -0.8)
	• Usual care	Nurses	109 children		Post-test =30/109		
Roukema et al,2008	• CDSS use	Nurses	74 children	Time spent in ED (minutes), median (IQR)	27 months =138 (77)	-	15 minutes
	• Control	Nurses	90 children		27 months =123 (96)		
	• CDSS use	Nurses	74 children	Time spent in ED for lab test (minutes), median (IQR)	27 months =140 (68)	-	-20 minutes
	• Control	Nurses	90 children		27 months =160 (98)		
Snooks et al, 2014	• CDSS	17 Paramedics	436 adults	Mean length of episode of care (minutes)	CDSS Vs control	-	-5.7 min (-38.5 to 27.2)[†]
	• Control	19 Paramedics	343 adults				
Wells,2013	• CDSS	22 paramedics	436 adults	Respiratory rate recorded, %	1 year =405/436	-	-1.2% (-4.7 to 2.2)
	• Control	20 paramedics	341 adults		1 year =321/341		
	• CDSS	22 paramedics	436 adults	Pulse rate recorded	1 year =414/436	-	0.9% (-3.9 to 2.0)
	• Control	20 paramedics	341 adults		1 year =327/341		
	• CDSS	22 paramedics	436 adults	Consciousness recorded	1 year =405/436	-	-5.1% (-7.9 to -2.2)
	• Control	20 paramedics	341 adults		1 year =334/341		
Kroth et al, 2006	• CDSS use	164 Nurses	Not applicable	Proportion of erroneously recorded temperatures	9 months =248/45823	-	-0.8% (-0.9 to -0.6)
	• Control	173 Nurses	Not applicable		9 months =575/44339		

3. Documenting of events

Dowding et al,2012	• CDSS use<br>• CDSS non-use	Nurses<br>Nurses		Fall documentation ratio	Post-CDSS use Vs pre-CDSS use period	-	1.4 (0.03 to 73.7)†
	• CDSS use<br>• CDSS non-use			Hospital acquired pressure ulcer (HAPU) risk documentation ratio	Post-CDSS use Vs pre-CDSS use period	-	9.1 (1.95 to 42.5)†
Paulson et al, 2020	• CDSS use<br>• Usual care	Nurses<br>Nurses	44 adults<br>50 adults	Documentation of nutritional intake compared to requirements	10 months=37/44<br>10 months=2/50	-	80% (67 to 92)
	• CDSS use<br>• Usual care	Nurses<br>Nurses	44 adults<br>50 adults	Documentation of a nutritional care plan	10 months=31/44<br>10 months=8/50	-	54.4% (37.6 to 71.3)
	• CDSS use<br>• Usual care	Nurses<br>Nurses	44 adults<br>50 adults	Documentation of nutritional treatment	10 months=36/44<br>10 months=29/50	-	23.8% (6 to 41.6)
**4. Patient referrals**							
Snooks et al, 2014	• CDSS<br>• Control	17 Paramedics<br>19 Paramedics	436 adults<br>343 adults	Patients referred to falls service	1 year=42/436<br>1 year=17/343	-	4.7% (1.1 to 8.3)

Note: **‡,** calculated from reported information unless stated otherwise; **†**, as reported by study authors.

Supplementary Table 4: Summary of patient care outcomes results

Author & Year	Interventions	Health professionals	patient participants	Outcome measured	Outcome values reported	Change of value within a group‡	Risk difference (95% CI)‡
**1. Glycaemic control**							
Blaha et al, 2009	• CDSS (eMPC)<br>• Mathias protocol<br>• Bath-protocol	ICU Nurses	40 adults<br>40 adults<br>40 adults	Entire study time in target range (blood glucose)- mmol/l	After 48hrs=46%<br>After 48hrs=38.2%<br>After 48hrs=39.7%	-<br>-	Versus Mathias:<br>7.8% (-13.7 to 29.4)<br>Versus Bath<br>6.3% (-3.9 to 16.5)
	• CDSS (eMPC)<br>• Mathias protocol<br>• Bath-protocol	ICU Nurses	40 adults<br>40 adults<br>40 adults	Entire study mean blood glucose (SE)- mmol/l	Baseline=8.1(0.6)<br>48hrs=5.9(0.2)<br>Baseline=7.9(0.4)<br>48hrs=6.7(0.1)<br>Baseline=8.0(0.2)<br>48hrs=6.5(0.2)	-2.2 mmol/l<br>-1.2 mmol/l<br>-1.5 mmol/l	Versus Mathias:<br>-1 mmol/l<br>Versus Bath:<br>-0.7 mmol/l
Canbolat et al,2019	• CDSS (automated BG control)<br>• Standard protocol	Nurses<br>Physicians	33 adults<br>33 adults	Occasions for BG out of target (120 to 180 mg/dL) range	22 months =2101/5789<br>22 months =2977/5122	-	-21.8% (-23.7 to -20.0)
	• CDSS (automated BG control)<br>• Standard protocol			Occasions for BG out of target range due to insulin treatment	22 months =745/5789<br>22 months =2099/5122	-	-28.1% (-29.7 to -26.5)
Cavalcanti et al, 2009	• CDSS (computer-assisted insulin protocol)<br>• Control (Leuven protocol)<br>• Control (conventional treatment)	ICU Nurses<br>ICU Nurses<br>ICU Nurses	56 adults<br>58 adults<br>53 adults	Mean blood glucose (mmol/dL)	19 months =125<br>19 months =127.1<br>19 months =158.5	-<br>-	Versus Leuven<br>-2.1 mmol/dL<br>Versus conventional<br>-33.5 mmol/dL
	• CDSS (computer-assisted insulin protocol)<br>• Control (Leuven protocol)<br>• Control (conventional treatment)	ICU Nurses<br>ICU Nurses<br>ICU Nurses	56 adults<br>58 adults<br>53 adults	Patients with hypoglycaemia	19 months =12/56<br>19 months =24/58<br>19 months =2/53	-<br>-	Versus Leuven<br>-20% (-36.6 to -3.4)<br>Versus conventional<br>17.6% (5.7 to 29.5)
Cleveringa et al,2008	• CDSS use in diabetic patients<br>• Usual care	Nurses<br>Nurses	1699 adults<br>1692 adults	A1C<7%	Baseline=60.8%<br>1 year=68%<br>Baseline=61.6%<br>1 Year=64.2%	7.2%<br>2.6%	4.6% (2.7 to 6.5)
	• CDSS use in diabetic patients		1699 adults	Systolic BP<140	Baseline=41%<br>1 year=53.9%	12.9%	10.2% (7.9 to 12.5)

	• Usual care		1692 adults		Baseline=39.5%<br>1 year=42.2%	2.7%	
	• CDSS use in diabetic patients		1699 adults	Total cholesterol <4.5mmol/l	Baseline=36.2%<br>1 year=49.0%	10.5%	3.7% (1.2 to 6.2)
	• Usual care		1692 adults		Baseline=38.5%<br>1 year=45.3%	6.8%	
Hovorka et al, 2007	• CDSS (eMPC)	ICU Nurses	30 adults	Proportion in target range (4-6.1 mmol/L)	48 hrs =60.4%	-	32.9% (20.0 to 46.0)
	• Usual care	ICU Nurses	30 adults		48 hrs =27.5%		
	• CDSS (eMPC)<br>• Usual care			Entire study mean blood glucose (mmol/L) (SD)	48 hrs =6.2 (1.1)<br>48 hrs =7.2 (1.1	-	-1mmol/L
	• CDSS (eMPC)<br>• Usual care			Time in target range (hours)	48 hrs =14.5<br>48 hrs =6.6		7.9 hrs
Mann et al,2011	• CDSS use	ICU Nurses	18 adults	Occasions glucose range on target (80 to 110 mg/dl)	72 hrs =47%	-	6% (-7.7 to 19.7)
	• Paper protocol	ICU Nurses	18 adults		72 hrs =41%		
	• CDSS use<br>• Paper protocol	ICU Nurses<br>ICU Nurses		Occasions over target range (over 110 mg/dl)	72 hrs =49%<br>72 hrs =54%	-	-5% (-18.8 to 8.8)
	• CDSS use<br>• Paper protocol			Occasions under target (under 80 mg/dl) range	72 hrs =4.5%<br>72 hrs =4.8%	-	-0.3% (-2.1 to 1.5)
Plank et al, 2006	• CDSS (MPC) use	ICU Nurses	Not reported	Occasions within the target glycaemic range (80-110 mg/dl)	48 hrs =52%	-	33% (20.5 to 45.4)
	• Usual care	ICU Nurses	Not reported		48 hrs =19%		
	• CDSS (MPC) use<br>• Usual care	ICU Nurses<br>ICU Nurses	Not reported<br>Not reported	Improvement glycaemic control for 48 hours	48 hrs =65%<br>48 hrs =25%	-	40% (27.4 to 52.6)
	• CDSS (MPC) use<br>• Usual care		Not reported<br>Not reported	Occasions over the target glycaemic range (>110 mg/dl)	48 hrs =46%<br>48 hrs =77%	-	-31% (-43.7 to -18.2)
	• CDSS (MPC) use<br>• Usual care		Not reported<br>Not reported	Average glucose (mg/dl)	48 hrs =117mg/dL<br>48 hrs =131 mg/dL	-	-14mg/dL
**2. Blood coagulation management**							
Fitzmaurice et al,2000	• CDSS use	Nurses	122 adults	proportion of tests in range	Baseline=223/366<br>1 year =732/1181	1.1%	-1.9% (-3.1 to -0.7)
	• CDSS non-use	Physicians	245 adults		Baseline=264/480<br>1 year =986/1700	3%	
	• CDSS use	Nurses		International Normalised Ratio (INR) Results Within Range Point Prevalence	Baseline=74/118<br>1 year =86/121	8.4%	-2.6% (-5.3 to -0.1)
	• CDSS non-use	Physicians			Baseline=129/244	11%	

					1 year =157/245		
	• CDSS use	Nurses		Time Spent Within INR Target Range	Baseline=64/113<br>1 year =76/110	12%	7% (-0.7 to 14.7)
	• CDSS non-use	Physicians			Baseline=99/174<br>1 year= 143/230	5%	
**3. Antenatal and peripartum care**							
Dalaba et al, 2015	• CDSS use	Nurses	Not reported	Antenatal complications per 1000 attendance	Before=9<br>After =12	0.3%	0.3% (-0.03 to 0.6)
	• CDSS non-use	Nurses	Not reported		Before =16<br>After =16	0%	
	• CDSS use			Delivery complications per 1000 attendances	Before=107<br>After=96	-0.9%	2.4% (1.1 to 3.7)
	• CDSS non-use				Before=133<br>After=100	-3.3%	
**4. Managing patients with chronic co-morbid diseases**							
McDonald et al, 2017	• CDSS use	165 Nurses	2550 adults	Medication regimen complexity index <24.5	Post-test=158/2550	-	0% (-1.1 to 1.1)
	• Usual care	335 Nurses	5369 adults		Post-test =333/5369		
	• CDSS use	165 Nurses	2550 adults	Emergency room use	Post-test =421/2550	-	-0.2 (-1.9 to 1.6)
	• Usual care	335 Nurses	5369 adults		Post-test =897/5369		
	• CDSS use	165 Nurses	2550 adults	Hospitalisation	Post-test =502/2550	-	-1.4% (-3.3 to 0.5)
	• Usual care	335 Nurses	5369 adults		Post-test =1133/5369		
Lv et al, 2019	• CDSS use	Nurses	70 children	Number of asthma exacerbations per patient (median)	1 year=3	-	-1
	• Usual care	Nurses	73 children		1 year=4	-	
**5. Outpatient obesity screening**							
Lee et al,2009	• CDSS use	13 Nurses	807 adults	Encounters with obesity related diagnosis	8 months =91/807	-	10.3% (8.0 to 12.5)
	• Usual care	16 Nurses	997 adults		8 months =10/997		
	• CDSS use	13 Nurses	807 adults	Encounters with missed obesity-related diagnosis	8 months =51/208	-	-41.9% (-48.8 to -35.1)
	• Usual care	16 Nurses	997 adults		8 months =440/662		
**6. Fall and pressure ulcer management**							
Beeckman et al, 2013	• CDSS(Pre-vPlan)	65 Nurses and physios	225 adults	Pressure ulcer prevention	Day1=15/58<br>Day120=41/65	37.2%	2.3% (-11.0 to 15.6)
	• Standard protocol	53 Nurses and physios	239 adults		Day1=16/63<br>Day120=41/68	34.9%	
	• CDSS(Pre-vPlan)	65 Nurses and physios	225 adults		Day 1=34/225<br>Day120=16/225	-8%	-6.3% (-10.2 to -2.4)

	• Standard protocol	53 Nurses and physios	239 adults	Prevalence of pressure ulcer	Day1=39/239<br>Day120=35/239	-1.7%	
Byrne,2005	• CDSS use	89 Nurses	Not reported	Fall rate	Before=0.312<br>After=0.318	0.6%	3.1%
	• CDSS non-use		Not reported		before=0.315<br>After=0.29	-2.5%	
	• CDSS use		Not reported	Pressure ulcer rate	Before=0.085<br>After=0.088	-0.3%	-0.6%
	• CDSS non-use		Not reported		Before=0.091<br>After=0.094	0.3%	
Dowding et al,2012	• CDSS use<br>• CDSS non-use			Fall rate	Post-CDSS use Vs pre-CDSS use period	-	0.91 (0.75 to 1.12)†
	• CDSS use<br>• CDSS non-use			HAPU ratio	Post-CDSS use Vs pre-CDSS use period	-	0.47 (0.25 to 0.85)†
Dykes et al, 2009	• CDSS use<br>• Usual care	Nurses<br>Nurses	5160 adults<br>5104 adults	Fall rate difference (per 1000 patient days)	CDSS use Vs usual care	-	-1.16 (-2.16 to -0.17) †
Dykes et al, 2020	• UDSS use<br>• CDSS non-use	Nurses<br>Nurses	19,283 adults<br>17,948 adults	Fall rate difference (per 1000 patient days)	Post-CDSS use Vs pre-CDSS use period	-	-0.15 (-0.04 to -0.25) †
Fossum et al,2011	• CDSS use	Nurses	367 adults	Prevalence of pressure Ulcers	Before=16/167<br>After=23/200	1.9%	4.2% (0.2 to 8.2)
	• CDSS non-use	Nurses	274 adults		Before=17/150<br>After=11/122	-2.3%	
	• CDSS use			Prevalence of malnutrition	Before=45/161<br>After=39/199	-8.3%	-12.4% (-19.1 to -5.7)
	• CDSS non-use				Before=31/148<br>After=30/120	4.1%	
**7. Triaging**							
Bennett et al, 2016	• CDSS use period<br>• CDSS non-use	Nurses<br>Nurses	400 adults<br>400 adults	Correct triage prioritisation	Post-test=85.2%<br>Pre-test=60.5%	-	24.7% (18.8 to 30.6)
Lattimer et al, 1998	• CDSS<br>• Usual care	Nurses<br>Physicians	Not applicable<br>Not applicable	Calls managed with telephone advice from GP	Post-test =1109/7184<br>Post-test =3629/7308	-	-34.2% (-35.6 to -32.8)
	• CDSS<br>• Usual care	Nurses<br>Physicians		Patient attended primary care centre	Post-test =1177/7184<br>Post-test =1934/7308	-	-10% (-11.4 to -8.8)
	• CDSS<br>• Usual care	Nurses<br>Physicians		Patient visited at home by duty GP	Post-test =1317/7184<br>Post-test =1745/7308	-	-5.5% (-6.9 to -4.2)
Lattimer et al, 2000	• CDSS<br>• Usual care	Nurses<br>Physicians		Total admissions within 3 days	1 year =428/7184<br>1 year =507/7308	-	-0.98% (-1.8 to -0.2)
	• CDSS	Paramedics	436 adults		1 year =183/436	-	5.2% (-1.7 to 12.1)

Snooks et al, 2014	• Control	Paramedics	343 adults	Patients left at scene without conveyance to emergency department	1 year =126/343		
	• CDSS<br>• Control		436 adults<br>343 adults	Patients with further emergency admission to hospital or death	1 year=69/436<br>1 year =49/343	-	1.5% (-3.5 to 6.6)
	• CDSS<br>• Control			Patients with ED attendance or emergency admission to hospital or death	1 year =92/436<br>1 year =61/343	-	3.3% (-2.3 to 8.9)
	• CDSS<br>• Control			Patients who reported >1 further fall	1 year =135/236<br>1 year =112/175	-	-6.8% (-16.3 to 2.7)
**8. Quality of life and patients' satisfaction**							
Cleveringa et al,2010	• CDSS use<br>• Usual care			Life-years gained	CDSS Vs usual care	-	0.14 (-0.12 to 0.40)†
	• CDSS use<br>• Usual care			Healthy years (QALYs, discounted)	CDSS Vs usual care	-	0.037 (-0.066 to 0.14)†
Snooks et al, 2014	• CDSS<br>• Control	Paramedics<br>Paramedics	239 adults<br>177 adults	Quality of Life (SF12 MCS), mean (SD)	1 year =41.9(10.3)<br>1 year =42.9(10.9)	-	-1 (-3.1 to 1.1)
	• CDSS<br>• Control	Paramedics<br>Paramedics	239 adults<br>177 adults	Quality of Life (SF12 PCS), mean (SD)	1 year=29(8)<br>1 year=30(8.5)	-	-1 (-2.6 to 0.6)
	• CDSS<br>• Control	Paramedics<br>Paramedics	228 adults<br>165 adults	Patient satisfaction (QC Technical), mean (SD)	1 year =97.8(10.7)<br>1 year=98.2(9.4)	-	-0.4 (-2.4 to 1.6)

Note: **‡,** calculated from reported information unless stated otherwise; **†**, as reported by study authors.

Supplementary Table 5: Summary of Health professionals' knowledge, beliefs and behaviour results

Author & Year	Interventions	Health professionals	patient participants	Outcome measured	Outcome values reported	Change of value within a group‡	Mean or risk difference (95% CI)‡
Beeckman et al, 2013	• CDSS(Pre-vPlan)	65 Nurses and physios	225 adults	Positive knowledge change	Baseline=28/65<br>5 months=26/50	8.9%	6.5% (0.8 to 13.2)
	• Standard protocol	53 Nurses and physios	239 adults		Baseline=21/53<br>5 months=16/38	2.4%	
	• CDSS(Pre-vPlan)	65 Nurses and physios	225 adults	Positive attitude change	Baseline=48/65<br>5 months=42/50	10.2%	12.7% (5.9 to 19.5)
	• Standard protocol	53 Nurses and physios	239 adults		Baseline=39/53<br>5 months=27/38	-2.5%	
Cortez, 2014	• CDSS (drop-down boxes)	26 Nurses	NA	Research utilisation	Baseline=35%<br>11 weeks=38%	3%	9% (3.3 to 14.7)
	• Control	24 Nurses	NA		Baseline=19%<br>11 weeks=13%	-6%	
Dumont et al,2012	• CDSS use	Nurses (OA=44)	141 adults	Nurses satisfaction, out of 10 (mean (SD))	4 months=8.4(1.4)	-	3.6 (2.4 to 4.8)
	• Paper protocol	Nurses	159 adults		4 months=4.8(2.4)		
	• CDSS use<br>• Paper protocol			perception of how often needed to deviate from the protocol, out of 10 (mean (SD))	4 months=2.7(2.2)<br>4 months=7.4(2.4)	-	-4.7 (-6.1 to -3.3)
Sassen et al,2014	• CDSS use	42 nurses and physios	Not reported	Behaviour, mean (SD)	Baseline=4.5 (1.02)<br>17 months=4.6 (0.85)	0.1 (0.93)	0.1 (-0.32 to 0.53)
	• Control	27 nurses and physios	Not reported		baseline=4.8 (0.69)<br>17 months=4.8 (0.82)	0 (0.75)	
	• CDSS use	42 nurses and physios		Intention, mean (SD)	Baseline=6.3 (1.0)<br>17 months=6.1 (1.1)	0.2 (1.05)	0.3 (-0.22 to 0.82)
	• Control	27 nurses and physios			Baseline=5.9 (1.15)<br>17 months=6.0 (0.91)	-0.1(1.05)	
	• CDSS use	42 nurses and physios		Attitude, mean (SD)	Baseline=6.3 (0.44)<br>17 months=6.3 (0.56)	0.0(0.05)	-0.1 (-0.13 to -0.07)
	• Control	27 nurses and physios			Baseline=6.2 (0.69)<br>17 months=6.3 (0.68)	0.1 (0.09)	
	• CDSS use	42 nurses and physios		Perceived behavioural control, mean (SD)	Baseline=4.7 (0.79)<br>17 months=5.0 (0.73)	0.3 (0.77)	-0.1 (-0.49 to 0.29)

• Control	27 nurses and physios		Baseline=4.9 (0.87)<br>17 months=5.3 (0.8)	0.4 (0.85)	
• CDSS use	42 nurses and physios	Subjective norms, mean (SD)	Baseline=5.5 (0.55)<br>17 months=5.6 (0.63)	0.1 (0.59)	0 (0.34 to 0.34)
• Control	27 nurses and physios		Baseline=5.6 (0.93)<br>17 months=5.7 (0.76)	0.1 (0.84)	
• CDSS use	42 nurses and physios	Moral norms, mean (SD)	Baseline=6.0 (0.63)<br>17 months=6.2 (0.7)	0.2 (0.67)	0.1 (-0.21 to 0.41)
• Control	27 nurses and physios		Baseline=6.2 (0.59)<br>17 months=6.3 (0.55)	0.1 (0.57)	
• CDSS use	42 nurses and physios	Barriers, mean (SD)	Baseline=3.1 (1.17)<br>17 months=3.2 (1.12)	0.1 (1.14)	0.3 (-0.23 to 0.83)
• Control	27 nurses and physios		Baseline=2.8 (1.01)<br>17 months=2.6 (0.96)	-0.2 (0.98)	

Note: **‡,** calculated from reported information unless stated otherwise; **†**, as reported by study authors.

Supplementary Table 6: Summary of adverse events results

Author & Year	Interventions	Health professionals	patient participants	Outcome measured	Outcome values reported	Risk difference (95% CI)‡
Cleveringa et al,2010	• CDSS use in diabetic patients	Nurses	1699 adults	cardiovascular events occurring	CDSS Vs usual care	-11% (-18 to -4)†
	• Usual care	Nurses	1692 adults			
Fitzmaurice et al,2000	• CDSS Nurse	Nurses	224 adults	Serious adverse reaction events	1 year =3 (1.3%)	-5.7% (-10.1 to -1.2)
	• CDSS non-use	Physicians	143 adults		1 year =10 (7%)	
	• CDSS Nurse	Nurses	224 adults	Deaths	1 year =3 (1.3%)	-5% (-9.2 to -0.7)
	• CDSS non-use	Physicians	143 adults		1 year =9 (6.3%)	
Snooks et al, 2014	CDSS	17 Paramedics	436 adults	Patients dying	1 year =19/436 (4.4%)	1.2% (-1.5 to 3.8)
	Control	19 Paramedics	343 adults		1 year=11/343 (3.2%)	

Note: **‡,** calculated from reported information unless stated otherwise; **†**, as reported by study authors.

Supplementary Table 7: Summary of economic costs and consequences results

Author & Year	Interventions	Health professionals	patient participants	Outcome measured	Outcome values reported	Difference (95% CI)‡
Cleveringa et al,2010	• CDSS use<br>• Usual care	Nurses<br>Nurses		Diabetes-related costs (excluding CHD)-€ discounted	CDSS Vs usual care	1,698.00 (187 to 3,209)†
	• CDSS use<br>• Usual care			Cardiovascular disease cost-€ discounted	CDSS Vs usual care	-587.00 (-880 to -294)†
	• CDSS use<br>• Usual care			Diabetic care protocol cost-€ discounted	CDSS Vs usual care	316.00 (315 to 318)†
	• CDSS use<br>• Usual care			Total cost-€ discounted	CDSS Vs usual care	1,415.00 (-130 to 2,961)†
	• CDSS use<br>• Usual care			Total costs per QALY gained (Euro)	CDSS Vs usual care	38,243.00†
Guerts et al, 2017	• CDSS use<br>• Usual care	Nurses<br>Nurses	113 children<br>109 children	Average emergency department visit costs (Euro)	156.4<br>156.4	0.00
	• CDSS use<br>• Usual care			Average diagnostics cost (Euro)	1.09<br>1.55	-0.46
	• CDSS use<br>• Usual care			Average treatment cost (Euro)	4.48<br>2.58	1.90
	• CDSS use<br>• Usual care			Average follow-up/hospitalization (Euro)	134.<br>107.4	26.60
	• CDSS use<br>• Usual care			Average costs of missed diagnoses/adverse events (Euro)	49.70<br>81.8	-32.10
	• CDSS use<br>• Usual care			Average cost of CDSS implementation (Euro)	61.95<br>0.0	61.95
	• CDSS use<br>• Usual care			Overall average cost	408<br>350	58.00
Lattimer et al,2000	• CDSS<br>• Usual care	Nurses<br>Physicians	Not applicable<br>Not applicable	Net savings [of CDSS use] in a year (£)	CDSS Vs usual care	13,185 (-77,509 to 123,824)†
	• CDSS<br>• Usual care			Cost saved from inpatient stay	CDSS Vs usual care	51,059†
Snooks et al, 2014	• CDSS<br>• Control	Paramedics<br>Paramedics		Implementing cost of CCDS in one month (in 100s £)	74	74
	• CDSS<br>• Control			Total cost of implementation in one month (in 100s £)	2,773<br>2,526	247 (-247 to 741)†
	• CDSS			Net resources saved		39†

• Control	by CDSS per patient year (£)		
• CDSS<br>• Control	Net cost resources saved by CCDS per patient year (£)		208-308[†]
• CDSS<br>• Control	Mean length of Job cycle time (minutes)	CDSS Vs control	8.9 min (2.3 to 15.3)[†]
• CDSS<br>• Control	Mean length of episode of care (minutes)	CDSS Vs control	-5.7 min (-38.5 to 27.2)[†]

Note: **‡,** calculated from reported information unless stated otherwise; **†**, as reported by study authors; PCS, physical component summary; MCS, mental component summary; SF, Short-Form
