# Supplementary material for: Effects of computerised clinical decision support systems (CDSS) on nursing and allied health professional performance and patient outcomes: a systematic review of experimental and observational studies
Source: BMJ Open. 2021 Dec 15;11(12):e053886. doi: 10.1136/bmjopen-2021-053886 (PMC8679061; doi:10.1136/bmjopen-2021-053886)
Supplement: Author's manuscript [file bmjopen-2021-053886.draft_revisions.pdf]

BMJ Open

BMJ Open is committed to open peer review. As part of this commitment we make the peer review history of every article we publish publicly available.

When an article is published we post the peer reviewers' comments and the authors' responses online. We also post the versions of the paper that were used during peer review. These are the versions that the peer review comments apply to.

The versions of the paper that follow are the versions that were submitted during the peer review process. They are not the versions of record or the final published versions. They should not be cited or distributed as the published version of this manuscript.

BMJ Open is an open access journal and the full, final, typeset and author-corrected version of record of the manuscript is available on our site with no access controls, subscription charges or pay-per-view fees (http://bmjopen.bmj.com).

If you have any questions on BMJ Open's open peer review process please email info.bmjopen@bmj.com

BMJ Open

Effects of computerised clinical decision support systems (CDSS) on nursing and allied health professional performance and patient outcomes: A systematic review

Journal:	*BMJ Open*
Manuscript ID	bmjopen-2021-053886
Article Type:	Original research
Date Submitted by the Author:	26-May-2021
Complete List of Authors:	Mebrahtu, Teumzghi; University of Leeds<br>Skyrme, Sarah; University of Leeds<br>Yang, Huiqin; University of Leeds<br>Andre, Deirdre; University of Leeds<br>Ledward, Alison; University of Leeds<br>King, Henry; University of Leeds<br>Randell, Rebecca; University of Bradford, Faculty of Health Studies<br>Keenan, Anne-Maree; University of Leeds, Leeds Institute of Rheumatology and Musculoskeletal Medicine<br>Bloor, Karen; University of York, Department of Health Sciences<br>Thompson, Carl; University of Leeds Faculty of Medicine and Health, Applied Health Research
Keywords:	Health informatics < BIOTECHNOLOGY & BIOINFORMATICS, Information technology < BIOTECHNOLOGY & BIOINFORMATICS, Information management < BIOTECHNOLOGY & BIOINFORMATICS

SCHOLARONE™
Manuscripts

I, the Submitting Author has the right to grant and does grant on behalf of all authors of the Work (as defined in the below author licence), an exclusive licence and/or a non-exclusive licence for contributions from authors who are: i) UK Crown employees; ii) where BMJ has agreed a CC-BY licence shall apply, and/or iii) in accordance with the terms applicable for US Federal Government officers or employees acting as part of their official duties; on a worldwide, perpetual, irrevocable, royalty-free basis to BMJ Publishing Group Ltd ("BMJ") its licensees and where the relevant Journal is co-owned by BMJ to the co-owners of the Journal, to publish the Work in this journal and any other BMJ products and to exploit all rights, as set out in our licence.

The Submitting Author accepts and understands that any supply made under these terms is made by BMJ to the Submitting Author unless you are acting as an employee on behalf of your employer or a postgraduate student of an affiliated institution which is paying any applicable article publishing charge ("APC") for Open Access articles. Where the Submitting Author wishes to make the Work available on an Open Access basis (and intends to pay the relevant APC), the terms of reuse of such Open Access shall be governed by a Creative Commons licence – details of these licences and which Creative Commons licence will apply to this Work are set out in our licence referred to above.

Other than as permitted in any relevant BMJ Author's Self Archiving Policies, I confirm this Work has not been accepted for publication elsewhere, is not being considered for publication elsewhere and does not duplicate material already published. I confirm all authors consent to publication of this Work and authorise the granting of this licence.

For peer review only

Effects of computerised clinical decision support systems (CDSS) on nursing and allied health professional performance and patient outcomes: A systematic review

Mebrahtu TF[1*], Skyrme S[2], Randell R[3], Keenan AM[2], Bloor K[4], Yang H[2], Andre D[5], Ledward A[2], King H[2], Thompson CA[2]

[1]School of Computing, Faculty of Engineering and Physical Sciences, University of Leeds, Leeds, UK

[2]School of Healthcare, University of Leeds, Leeds, UK

[3] Faculty of Health Studies, University of Bradford, Bradford, UK; Wolfson Centre for Applied Health Research, Bradford, UK

[4]Department of Health Sciences, University of York, York, UK

[5] Library Services, University of Leeds, Leeds, UK.

***corresponding author:** t.f.mebrahtu@leeds.ac.uk; School of Computing, Faculty of Engineering and Physical Sciences, University of Leeds, Leeds, LS2 9JT

Keywords: decision support; nursing; allied health professionals; CDSS; decision making

Word count:

Abstract=296

Main text=3953

For peer review only - http://bmjopen.bmj.com/site/about/guidelines.xhtml

ABSTRACT

Objective: Digital technology designed to support decision making is an increasingly important part of nurse and allied health professional(AHP) roles in delivering healthcare. The impact of these technologies on professionals and patient outcomes has not been systematically reviewed. We aimed to conduct a systematic review to investigate this.

Materials and Methods: Various databases(including MEDLINE, EMBASE and CINAHL) were searched for published and unpublished research from inception to February 2021 without language restrictions. Any comparative research studies comparing CDSS with usual care were eligible for inclusion.

Results: A total of 36,106 non-duplicate records were identified. Of 35 studies included: 28 were randomised trials, three controlled-before-and-after studies, three interrupted-time-series and one non-randomised trial. There were ~1,318 health professionals and ~67,595 patient participants in the studies. Most studies focused on nurse decision makers(71%) or paramedics(5.7%). CDSS as a standalone, PC/LAPTOP-technology was a feature of 88.7% of the studies; only 8.6% of the studies involved "smart" mobile/handheld-technology.

Discussion: CDSS generated a positive effect in 38% of the outcome measures used. Care processes were positively influenced in 47% of the measures adopted. For example, nurses' adherence to hand disinfection guidance, insulin dosing, on-time blood sampling, and documenting care were better if they used CDSS. Patient care outcomes in 40.7% of indicators were better. For example, lower numbers of falls and pressure ulcers, better glycaemic control, screening of malnutrition and obesity, and triaging of were features of professionals using CDSS compared to those who did not.

Conclusion: CDSS can positively impact on selected aspects of nurses' and AHPs' performance and care outcomes. However, comparative research is generally low quality, with a wide range of heterogeneous outcomes. After more than 13 years of synthesised research into CDSS in healthcare professions other than medicine, the need for better quality evaluative research remains as pressing.

For peer review only - http://bmjopen.bmj.com/site/about/guidelines.xhtml

For peer review only

Strengths and limitations of the review:

- The review is based on a comprehensive literature search
- This is the first systematic review of CDSS influence on nursing and AHP performance and outcomes
- Allied Health Professionals are under-represented, with a primary focus on paramedics and physiotherapists
- The number of studies, service users/patients, and health professionals involved was sizable, but outcomes were too heterogeneous to aggregate
- The overall quality of comparative research represented by the included studies was poor.

For peer review only

For peer review only - http://bmjopen.bmj.com/site/about/guidelines.xhtml

INTRODUCTION

Nurses and allied health professionals' (AHPs') judgements and decisions commit financial, human, and technical resources to care in health systems.[1] To support decision making and underpin new roles and ways of delivering services, such as nurse-led primary care,[1] computerised clinical decision support systems (CDSS) have been developed to tailor evidence-based advice provided to clinicians at the point of decision making.

CDSS can improve professional performance by making the basis for decisions explicit; widen available information, encourage more consistent decisions and thus reduce unwarranted variation in processes and patient outcomes.[2 3] CDSS, may also encourage a focus on unimportant problems, hinder care delivery and contribute to a widening of (digital) inequalities.[4-6]

Reviews focusing mainly on doctors, suggest CDSS effects on performance and outcomes are inconsistent [7] but improved care processes[8 9] and reduced morbidity [8] and mortality [10] are possible. These reviews, however, often neglect the multi-disciplinary nature of healthcare delivery and the decisions involved.

Previously synthesised studies of nurses' use of CDSS suggest only limited impact on performance and health outcomes.[11] Digital technology and research evidence have both developed significantly since this review was undertaken. In this review we aim to examine CDSS impact on nurses' and AHP's performance and patient outcomes in the light of developed research and technology.

REVIEW METHODS

Following best practice principles [12 13] we undertook a systematic review of research into CDSS targeting nurse and AHP decision makers. The protocol was registered with PROSPERO [14] [number: CRD42019147773].

Literature searching

Initial searches were conducted in November 2019 and updated on 15 February 2021. Searches were not restricted by language. See Supplementary Table 1 for search terms.

For peer review only - http://bmjopen.bmj.com/site/about/guidelines.xhtml

We searched: MEDLINE(Ovid) , Embase Classic+Embase (Ovid), PsycINFO (Ovid), HMIC (Ovid) Health Management Information Consortium, AMED (Allied and Complementary Medicine) (Ovid) , CINAHL , Cochrane Central Register of Controlled Trials (Wiley, Cochrane Database of Systematic Reviews (Wiley), Social Sciences Citation Index Expanded (Clarivate), ProQuest Dissertations & Theses Abstracts & Index, ProQuest ASSIA (Applied Social Science Index and Abstract), Clinical Trials.gov, World Health Organisation International Clinical Trials Registry (ICTRP), Health Services Research Projects in Progress (HSRProj), OpenClinical(www.OpenClinical.org), OpenGrey (www.opengrey.eu), Health.IT.gov, Agency for Healthcare Research and Quality (www.ahrq.gov).

Study inclusion and exclusion

After removing duplicate titles and abstracts, six reviewers (AK, CT, HY, HK RR, SS and TM) independently screened all titles and abstracts. Two reviewers (CT and TM) assessed study relevance using Cochrane Collaboration's Effective Practice and Organisation of Care (EPOC) criteria.[15]

Comparative studies (randomised controlled trials (RCTs), non-randomised trials, controlled before-after (CBA) studies, interrupted time series (ITS) studies and repeated measures studies) comparing CDSS against usual care (i.e., clinical decision making unsupported by CDSS) were eligible for inclusion.

Outcomes

Our primary outcome was adherence of nurses and AHPs to evidence-based recommendations. Secondary outcomes were diagnostic accuracy, time to reach judgment, adverse events, health professional satisfaction, and system and/or implementation costs.

Data extraction

Data on study characteristics and outcomes were independently extracted by two reviewers (CT and TM) using the EPOC standard data collection form.[16]

Quality assessment

For peer review only - http://bmjopen.bmj.com/site/about/guidelines.xhtml

Study quality and risk of bias was assessed independently by CT and TM using Cochrane Handbook for Systematic Reviews of Interventions[17] and EPOC guidelines.[18]

Each potential source of bias was judged as high, low, or unclear, and an overall 'risk of bias' classification (high, moderate, or low) assigned to each included study.[17] Studies with low risk of bias in all domains, or where bias was unlikely to fundamentally alter results, were treated as low risk. Studies with bias risk in at least one domain, or where bias might alter conclusions, were treated as unclear. Studies with a high risk of bias in at least one domain, or with a serious bias likely to reduce the certainty of conclusions, were considered high risk.

Data synthesis

Findings were narratively synthesised, regardless of statistical analysis in the primary study. Studies were grouped by i) similarity in focus or CDSS-type (knowledge based or machine learning), ii) health professionals targeted, iii) patient group, iv) outcomes reported, and, v) study design.

If not reported, we calculated absolute risks from the primary research. Risk differences and 95% confidence intervals were then calculated from these. Because the CDSS, participants, and underlying research questions were so heterogeneous no meta-analysis was undertaken.[19]

RESULTS

Evidence Quantity

From 36,106 non-duplicate records identified, 35,858 records were excluded after title and abstract screening. Seven records were identified through forward citation searching. Full text screening was undertaken on 255 records which led to 220 more records being excluded. Thirty-five studies were included in the review.[20-51] **Figure 1** illustrates study selection.

Study Descriptions

The 35 included studies comprised 28 RCTs (80%), three CBA studies (8.6%), three ITS (8.6%) and one non-randomised trial (2.8%). Thirty-two studies (91.4%) were peer-reviewed journal articles with three (8.6%) PhD theses. The public sector funded 74.3% of studies; industry, 5.7%; 17.1% failed to

For peer review only - http://bmjopen.bmj.com/site/about/guidelines.xhtml

declare funding and 2.9% were unfunded. Most studies were published after 2010 (n=29, 82.9%) with just two studies during 1997-1999 and 14 (40.0%) in 2000-2010. Sixteen studies (54.3%) were published after the last significant systematic review on CDSS for nurses' performance and health outcomes.[11] Circa 1,318 health professionals and 67,595 patients were study participants, mainly in hospital-based studies (57.1%). Primary care accounted for 17.1% and nursing homes 11.4% of studies. Western health systems provided the dominant context: US (28.6%); UK (20.0%), Netherlands (17.2%), Czech Republic and Norway (5.7%) each. With single study representation (2.8%) from Belgium, Brazil, China, Ghana, Norway, Sweden, Turkey and one multicentre (Austria, Czech Republic, and UK) report. See **Table 1**.

Only one study (of 35) reported had an explicit theory to guide implementation of the CDSS. Almost a third (28%) published their study protocol – none of which discussed theory-influenced implementation.

Nurses made up the target for the CDSS *and* control groups in 25 (71.4%) studies; paramedics in two (5.7%) studies. Five studies (14.3%) compared nurses in the intervention (CDSS) group with physicians in the control. Two studies (5.7%) recruited a combination of nurses and physiotherapists for CDSS and control groups. Thirty-one studies (88.7%) used a standalone computer-based CDSS; three (8.6%) used handheld/mobile-based technologies, and just one study (0.2%) using a web-based CDSS. CDSS were mostly designed with a single function in mind (e.g., disease diagnosis), but some addressed multiple parts of clinical pathways (e.g., disease diagnosis *and* disease management).

Quality of identified evidence

Except for three RCTs scored as 'Unclear', all studies were at 'high' overall risk of bias. On average, RCTs scored 'Low' risk of bias in five of nine domains; CBA studies were lower, with four domains; non-randomised studies scored 'low' for a single domain. The three ITS studies were 'Low' risk of bias in six (of seven) domains. Evidence quality did not change over time (see Supplementary Table 2).

For peer review only - http://bmjopen.bmj.com/site/about/guidelines.xhtml

Figure 1 PRISMA Flow chart of study selection process

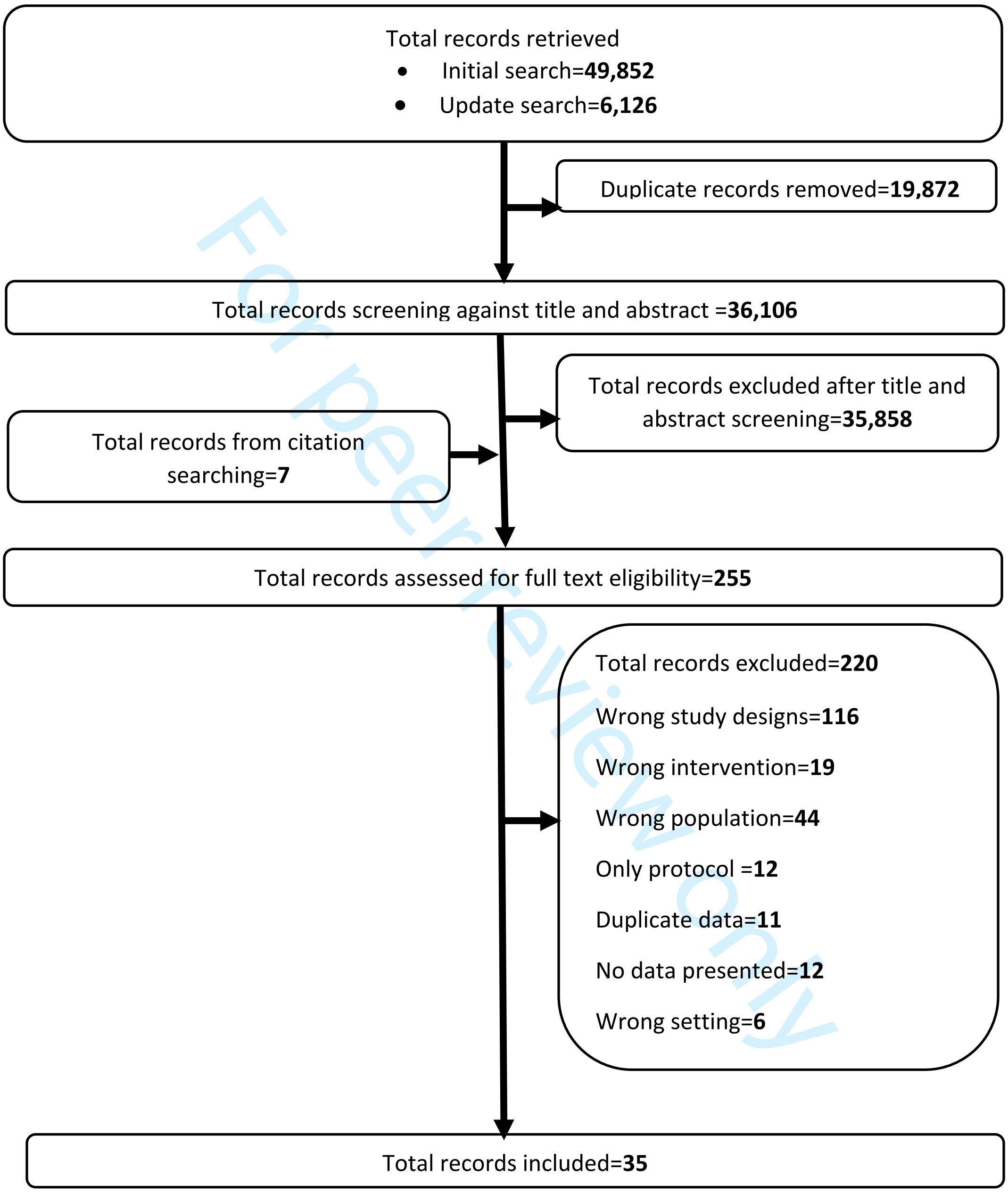

For peer review only

Table 1 Baseline characteristics of included studies

Author and year	Country	Design	Setting	Study duration	Healthcare professionals (HP)	Outcomes
Beeckman et al, 2013	Belgium	RCT	Nursing homes	5 months	Nurses and physios	Risk of pressure ulcers; HP knowledge and attitude
Bennet et al, 2016	UK	ITS	Emergency department, district general hospital	1 year	Nurses	Triage prioritization; pain assessment and management; management of neutropenic sepsis
Blaha et al, 2009	Czech Republic	RCT	ICU post elective cardiac surgery university hospital	48 hours	Nurses	Intensive care glycaemic control/diabetes
Byrne, 2005	USA	CBA	Nursing homes	33 months	Nurses	Falls and pressure ulcer reduction (assessment and prevention)
Canbolat et al,2019	Turkey	Non-RT	ICU university general hospital	22 months	Nurses [and physicians]	ICU glycaemic control
Cavalcanti et al, 2009	Brazil	RCT	ICU general hospital	19 months	Nurses	ICU glycaemic control
Cleveringa et al, 2008	Netherlands	RCT	Primary care practices	1 year	Nurses [and physicians)	Management and prevention of diabetes (and CV risk factors)
Cleveringa et al, 2010	Netherlands	RCT	Primary care practices	1 year	Nurses	Management and prevention of diabetes (and CV risk factors)
Cortez, 2014	USA	RCT	Academic medical centre oncology clinics	11 weeks	Nurses	Management of cancer symptoms
Dalaba 2015	Ghana	CBA	Primary care health centres	2 years	Nurses	Maternal care
Dowding et al,2012	USA	ITS	General hospitals	6 years	Nurses	Risk assessment, falls and pressure ulcer prevention
Duclos et al, 2015	France	RCT	Paediatric wards in a university hospital	2 years	Dieticians	Nutritional care in malnourished children
Dumont et al,2012	USA	RCT	ICU wards in a regional referral hospital	4 months	Nurses	Glycaemic control
Dykes et al, 2009	USA	RCT	Urban hospitals	6 months	Nurses	Fall prevention
Dykes et al, 2020	USA	ITS	Academic medical centres	42 months	Nurses	Fall prevention
Fitzmaurice et al,2000	UK	RCT	primary care/general practice	1 year	Nurses	oral anticoagulation care
Forberg et al, 2016	Sweden	RCT	paediatric university hospital	3 months	Nurses	management of peripheral venous catheters in paediatrics
Fossum et al, 2011	Norway	CBA	Nursing homes	2 years	Nurses	Preventative behaviours and management of nutrition

For peer review only - http://bmjopen.bmj.com/site/about/guidelines.xhtml

Geurts et al, 2017	Netherlands	RCT	University paediatric hospital	2 years	Nurses	Management of (re)hydration in children
Hovorka et al, 2007	Czech Republic	RCT	Cardiac Surgery, University Hospital	48 hours	Nurses	Glycaemic control
Kroth et al, 2006	USA	RCT	University Hospital	9 months	Nurses	Body temperature assessment
Lattimer et al, 1998	UK	RCT	Primary care practices	1 year	Nurses & physicians	Emergency call assessment
Lattimer et al, 2000	UK	RCT	Primary care practices	1 year	Nurses & physicians	Cost analysis of emergency call assessments
Lee et al, 2009	USA	RCT	School of Nursing (University)	8 months	Nurses	Obesity management
Lv et al, 2019	China	RCT	Community healthcare centres	1 year	Nurses	Chronic asthma management
Mann et al, 2011	USA	RCT	Surgical Military hospital ICU	6 days	Nurses	Glycaemic control in burn intensive care patients
McDonald et al, 2017	USA	RCT	Nursing care homes	2 months	Nurses	Management of chronic medical condition
Paulson et al, 2020	Norway	RCT	University hospital	10 months	Nurses	Management of malnutrition
Plank et al, 2006	Mixed (Austria, Czech Republic, UK)	RCT	University hospitals	48 hours	Nurses	Glycaemic control
Rood et al, 2005	Netherlands	RCT	Surgical ICU in a teaching hospital	10 weeks	Nurses	Glycaemic control
Roukema et al, 2008	Netherlands	RCT	Children's Hospital	27 months	Nurses	Management of children with fever without apparent source
Sassen et al, 2014	Netherlands	RCT	University research centre	17 months	Nurses and physios	professionals' behaviour
Snooks et al, 2014	UK	RCT	Emergency ambulance services	1 year	Paramedics	Assessment and management of falls
Vadher et al, 1997	UK	RCT	Cardiovascular medicine, general hospital		A nurse and Trainee doctors	oral anticoagulant control
Wells, 2013	UK	RCT	Emergency ambulance services	1 year	Paramedics	Emergency fall assessment and management

Note: CBA, controlled before and after; CDSS, computerised decision support; HPs, health professionals; ITS, interrupted time-series; RCT, randomised controlled trials

For peer review only - http://bmjopen.bmj.com/site/about/guidelines.xhtml

Effects of intervention

Most studies reported more than two outcomes from a total of 124 individual outcomes reported (115 distinct types of measured outcomes). There were five distinct outcome groups: 1) care process, 2) care outcomes, 3) health professionals' knowledge, beliefs, and behaviours, 4) adverse events, and 5) economic costs and consequences.

Care process

CDSS was better than usual care for 16 of 34 (47%) care process outcomes. Care delivery was worse (n=5, 14.7%) or no different for 13 (38.2%) processes. See Supplementary Table 3.

Adherence to guidelines

The four RCTs reporting nurses' adherence to guidelines examined 10 outcomes.[32 34 45 49] Only one trial reported baseline and follow-up data for both arms,[34] CDSS users had better adherence to hand disinfection guidelines (risk difference=6.7%; 95% CI: 4.9 to 8.5%); but were less likely to follow guidelines on disposable glove use (risk difference= -1.4%; 95% CI: -2.2 to -0.5%) and daily inspections of Peripheral Venous Catheters (risk difference=-5.2%; 95% CI: -7.2 to -3.3%).

Two trials [32 45] showed nurses using CDSS had better insulin dosing (risk difference=22%; 95% CI: 19 to 25%) and on-time blood sampling (risk difference=4.7%; 95% CI: 2.0 to 7.4%) guideline compliance. They deviated less from protocols (mean score difference out of 10 =-2.6; 95% CI: -4.5 to -0.71) and concurred more with recommended insulin doses (than trainee doctors).[49]

Patient assessment, diagnosis, and treatment practices

Five RCTs [31 36 38 46 50] and one ITS [21] reported 18 indicators of patient assessment and treatment quality. Pain assessment quality (pain score use and appropriateness of choices) of emergency department patients improved by 62.7% (95% CI: 59.6 to 65.8%) and investigation of in-patient paediatric malnutrition aetiology was 21.2% higher (95% CI: 15.9 to 26.5%) with CDSS. However, optimal IV antibiotics administration for sepsis was lower reduced by 5.9% (95% CI: -8.3 to -3.5). Laboratory tests

For peer review only - http://bmjopen.bmj.com/site/about/guidelines.xhtml

(electrolytes level acid-base balance test) and nutrition supplements (oral Rehydration Solution and IV rehydration) were no more likely to be ordered for paediatric inpatients by CDSS-enabled nurses.

There were marginally fewer wrongly recorded temperatures in hospital inpatients amongst CDSS-enabled nurses (risk difference= -0.8%, 95% CI: -0.9 to -0.6). Vital signs recording in patients attended by paramedics were also not significantly different.

Documenting care

One ITS and a randomised trial reported five documentation-focused indicators.[30 52] Falls (risk ratio=1.4, 95% CI: 0.03 to 73.7) and hospital acquired pressure ulcer risk assessments (risk ratio=9.1, 95% CI: 1.95 to 42.5) were higher with CDSS. As was nutritional care planning, food and fluid intake recording and treatment by nurses.[52]

Referrals

Paramedics using CDSS were more likely to refer patients to a community falls than send them to the ER (risk difference=4.7%, 95% CI: 1.1. to 8.3).[48]

Patient care outcomes

CDSS improved patient care outcomes in 22 of 54 (40.7%) indicators and worsened them for 1 outcome indicator (2%). See Supplementary Table 4.

Blood glucose control

Six RCTs [22 25 26 37 42 44] and one non-randomised trial [24] reported 19 indicators of glycaemic control, but only two reported baseline *and* follow-up values [22 26]. Blood glucose levels were better managed by ICU nurses using CDSS (mean=-2.2, SD=1.12) compared to paper-based *Mathias* (mean=-1.2, SD=0.66) and *Bath* (mean=-1.5, SD=0.78) protocols.[22] Glycated haemoglobin (A1C) <7%, systolic blood pressure <140 and total cholesterol<4.5mmol/l were higher by 4.6% (95% CI: 2.7 to 6.5), 10.2% (95% CI: 7.9 to 12.5) and 3.7% (95% CI: 1.2 to 6.2) respectively in patients receiving care from CDSS-enabled nurses compared.

For peer review only - http://bmjopen.bmj.com/site/about/guidelines.xhtml

Trials reporting only follow-up data suggest better blood glucose control by CDSS-using nurses across a range of indicators: proportion in target range (risk difference=32.9%; 95% CI: 20 to 46%), occasions within the target glycaemic range (80-110 mg/dl) (risk difference= 33%, 95% CI: 20.5 to 45.4), occasions over the target glycaemic range (>110 mg/dl) (risk difference= -31%, 95% CI: -43.7 to -18.2), and improvement of glycaemic control for 48 hours (risk difference=40%, 95% CI: 27.4 to 52.6)

Blood coagulation management

One RCT reported three indicators of blood coagulation management in primary care.[33] Nurses using CDSS had significantly more tests in range (risk difference=4%, 95% CI: 0.4 to 7.6) than doctors *without* CDSS. However, the improvement from baseline was lower amongst nurses (risk difference=-1.9% (95% CI: -3.1 to -0.7), 'International Normalised Ratio (INR) Results within Range Point Prevalence' were not significantly different between the two groups and again, nurses using CDSS improved less than physicians without CDSS (risk difference=-2.6%, 95% CI: -5.3 to -0.1). There was no significant difference between groups in 'Time Spent within INR Target Range' (risk difference=7%, 95% CI: -0.7 to 14.7).

Antenatal and peripartum care

The CBA study examining antennal and peripartum care in community settings[29] suggested CDSS-using midwives reduced delivery complications (per 1000 attendances) compared to usual care (risk difference=2.4%, 95% CI: 1.1 to 3.7).

Managing patients with chronic co-morbid diseases

Two RCTs examined three indicators of successfully managing patients with complex chronic multi-morbid health conditions in care homes,[43] and with asthma[53] showed no significant differences between CDSS users and non-users for emergency room usage, hospitalisation and complexity of medication regimens.

Obesity screening

For peer review only - http://bmjopen.bmj.com/site/about/guidelines.xhtml

The RCT examining outpatient obesity screening by trainee nurses found CDSS-users had more 'encounters with obesity-related diagnosis' (risk difference=10.3%, 95% CI: 8.0 to 12.5) and fewer 'encounters with missed obesity-related missed diagnosis' (risk difference=41%, 95% CI: 48.8 to 35.0) than trainee nurses without CDSS.[41]

Fall and pressure ulcer prevention and management

Two RCTs, [20 51] two CBA studies [23 35] and two ITS [30 54] focused on fall or pressure ulcer prevention and management. In a single trial,[20] pressure ulceration prevalence decreased more during the CDSS-enabled follow-up period (risk difference=-6.3%, 95% CI: -10.2 to -2.4). A result reversed in one of the CBA studies (risk difference=4.2%, 95% CI: 0.2 to 8.2).[35] The other CBA studies revealed no significant differences between CDSS using and non-using nurses trying to prevent falls and pressure ulcers.[23] In the ITS study, fall rate (risk ratio=0.91, 95% CI: 0.75 to 1.12) and hospital acquired pressure ulcer occurrence (risk ratio=0.47, 95% CI: 0.25 to 0.85) were significantly lower with CDSS.[30]

Triage

Three RCTs [39 40 48] and one ITS study [21] evaluated CDSS impact on triage judgements. Health professionals using CDSS made fewer calls to General Practitioners (GP) for telephone advice (risk difference= -34.2%, 95% CI: -36 to -33), had fewer patients visited at home by duty GPs (risk difference=-5.5%, 95% CI: -6.9 to -4.2), and fewer hospital admissions within 3 days (risk difference=-0.98%, 95% CI: -1.8 to -0.2) of the judgement. There were no differences in, 'patients left at scene without conveyance to emergency department' (risk difference= 5.2%, 95% CI: -1.7 to 12.1). The ITS study reported the proportion of *correct (sic)* triage prioritisation judgements was higher amongst CDSS-users (risk difference=24.7%; 95% CI: 18.8 to 30.6).

Quality of life and patients' satisfaction

Two RCTs examined CDSS impact on quality of life and patient satisfaction.[27 48] Patients in CDSS-using groups gained more life years (average difference in years=0.14, 95% CI: -0.12 to 0.40), more healthy years (average difference in years = 0.037, 95% CI: -0.066 to 0.14) but lower quality of life and satisfaction. None of these differences were statistically significant.

For peer review only - http://bmjopen.bmj.com/site/about/guidelines.xhtml

Health professionals’ knowledge, beliefs, and behaviour

CDSS effects on knowledge, beliefs, and behaviours of health professionals [20 28 32 47] was the focus of four RCTs using twelve indicators. CDSS increased ‘Positive knowledge change’ (risk difference=6.5%; 95% CI:0.8 to 13.2), ‘positive attitude change’ (risk difference=12.7%, 95% CI: 5.9 to 19.5), ‘research utilisation’ (risk difference=9%; 95% CI: 3.3 to 14.7), nurses’ satisfaction (difference in satisfaction out of 10=3.6, 95% CI: 2.4 to 4.8), and perceived deviations from protocols (mean difference out of 10=-4.7, 95% CI: -6.1 to -3.3). Conversely, there was no significant impact on behaviours, intentions, perceived behavioural control, subjective and moral norms, barriers, and research utilisation of CDSS-using nurses and physiotherapists (Supplementary Table 5).

Adverse events

CDSS are not risk free, and three RCTs [27 33 48] used four indicators to examine adverse events. Cardiovascular events in patients with diabetes (risk difference=-11%, 95% CI: -18 to -4) and deaths in primary care patients (risk difference=-5.7%, 95% CI: -10.1 to -1.7) were lower in CDSS-using groups of professionals. Serious adverse reactions in primary care patients and deaths in patients recently fallen and attended by paramedics were no less likely (Supplementary Table 6).

Economic costs and consequences

Four RCTs [27 36 40 48] used 20 indicators to report economic costs and consequences of CDSS. Costs of managing cardiovascular disease were lower in CDSS users (cost difference=-€587, 95% CI: -880 to -294). Diabetes care cost more (cost difference=€326, 95% CI: 315 to 318); took longer per care task (‘mean length of job cycle time’ difference in minutes=8.9; 95% CI: 2.3 to 15.3) to generate an additional quality adjusted life-year (QALY) costing €38,243.00 (Supplementary Table 7).

DISCUSSION

Summary of main results

Our systematic review suggests CDSS can improve nurses’ and AHPs’ performance and care outcomes. Thirty eight percent (38%) of indicators were better. Of 35 included studies, 26 (74.3%) reported CDSS-influenced care as better than care without CDSS on at least one outcome. In contrast, 8 studies

For peer review only - http://bmjopen.bmj.com/site/about/guidelines.xhtml

For peer review only

(22.8%) showed no impact of CDSS on care, with 7 studies suggesting CDSS were less effective than usual care for at least one outcome.

Care process

Processes of care were positively influenced by CDSS in almost half the studies: 16 of 34 (47%) a headline that masks a very wide range of absolute improvement: from 0.7% to 62.7%. Hand disinfection protocol adherence, insulin dosing, blood sampling at the right time, and documented care were all better in CDSS users. This should be contrasted with the five (16.1%) outcomes where CDSS provided no advantages over usual care. Both sets of findings are mitigated further by the considerable uncertainty in trying to estimate a holistic picture: the effects in 13 care process indicators (41.9%) were not estimable; either because studies lacked power (lower than minimum acceptable of 80%) to detect a difference in the comparison groups, or appropriate confidence intervals were not reported or could not be calculated from information published.

Patient care outcomes

CDSS significantly improved patient care outcomes across a broad range of 22 of 54 (40.7%) indicators (absolute improvement between 4.6% and 42.9%). Just one indicator (1.8%) suggested no improvement. Nurses using CDSS better controlled blood glucose in emergency care patients (in five out of seven studies involved) and nurses and physiotherapists using CDSS better managed fall risk and pressure ulcer management. Triage was improved in nurses using CDSS in emergency call centres and paramedics faced with "emergency falls" in older patients.

Health professionals' knowledge, beliefs, and behaviour

Improved knowledge, beliefs, and behaviour occurred in three of 12 indicators (25%). Nurse and physiotherapist CDSS-users had more knowledge and better attitudes compared to non-users. CDSS-enabled nurses utilised more research, were more satisfied at work, and perceived a greater need to follow protocols than non-users.

Adverse events

For peer review only - http://bmjopen.bmj.com/site/about/guidelines.xhtml

For peer review only

CDSS generated fewer adverse events across two of four indicators (50%). CDSS-using nurses had fewer cardiovascular events and reported deaths in their primary care patients compare to similar patients seen by doctors not using CDSS.

Economic costs and consequences

CDSS did not significantly increase costs or produce savings. Costs per quality adjusted life-year (QALY) was €38,243.00 in one study –higher than the widely accepted willingness-to-pay threshold of €20,000 per QALY[27] and the United Kingdom *de facto* threshold of £30,000 per QALY to be considered cost-effective by the National Institute for Health and Care Excellence.[55]

Comparison with other studies or reviews

Only one previous review has examined the effects of CDSS on nursing performance and patient outcomes.[11] Twenty new primary studies have been published since this review; but inconsistent outcomes and weaknesses in study designs and methods remain. Of note is the absence of a theoretical foundation for the implementation of CDSS in many studies and the absence of guidelines for designing, conducting/evaluating, and reporting CDSS-use by nurses/AHPs. Of 32 included studies, just one used an explicit implementation model/theory at design stage.[20] None of the studies discussed their findings with reference to implementation science/theory.

In their review of 100 trials – principally with doctors - Garg et al.[7] reported improved performance in 64% and better patient outcomes in 13% of studies. Our results suggest greater improvement may be possible for nursing work in particular (47% of process indicators and 41% of outcomes). Garg et al.[7] transformed improvement into a binary (yes/no) indicator and did not quantify the outcome improvements – making the clinical significance of improvements hard to ascertain.

Bright et al. [8] reviewed RCTs of CDSS with a range of health professional decision makers (doctors, nurses and AHPs). They reported improvements in processes of care (OR=1.55, 95% CI: 1.38 to 1.74) and morbidity (RR=0.88, 95% CI: 0.80 to 0.96), but no impact on mortality (OR=0.79, 95% CI: 0.54 to 1.15) or safety/adverse events (RR= 1.01, 95% CI: 0.90 to 1.14). However, outcomes measured were

For peer review only - http://bmjopen.bmj.com/site/about/guidelines.xhtml

too heterogeneous for meta-analysis. The criteria for comparison groups was relaxed; the "intervention" sometimes included paper-based decision support and alternative CDSS systems were used as a comparator in some studies. Our review required there to be an indication for the use of CDSS and a comparator that ruled out CDSS-use as part of "usual care". Whilst we found improvements are *possible* from CDSS, comparison with Bright et al's findings would be unreliable.

Moja and colleagues' review of 18 RCTs [10] (including nurses and AHPs alongside doctors) found no significant difference in CDSS-attributable mortality (RR=0.96, 95% CI: 0.85 to 1.08) but lower morbidity (RR=0.82, 95% CI: 0.68 to 0.99). Whilst mortality and morbidity findings are similar to ours, their use of CDSS in the primary study comparator groups, again makes comparisons unreliable.

A recent review of 115 trials of CDSS, with a mix of health professionals, reported process improvements of the order of 5.8% (95% CI: 4.0% to 7.6%) with CDSS.[9] As with Bright et al. the 'comparator' criteria were unclear and outcome measures too heterogeneous for meta-analysis. Studies with more than two comparators were treated as different trials, meaning double counting and multiple comparisons (p-hacking) could not be ruled out, confounding comparisons with our findings.

Strength and limitations

Our review, whilst based on a comprehensive literature search, is a function of that literature. Consequently, we have primarily highlighted CDSS impact on nurses rather than AHPs. With the exception of paramedics and physiotherapists, other AHPs are poorly represented.

Evidence quality was poor and has not improved significantly since the late noughties. Whilst the number of studies (35), service users/patients (~67,000) and health professionals (~1,318) involved was sizable, outcomes were too heterogeneous for aggregation. Inconsistencies in the effects of CDSS on target health professionals' performance and patient outcomes remains unresolved.

Conclusions

For peer review only - http://bmjopen.bmj.com/site/about/guidelines.xhtml

CDSS can benefit nurse and (some) AHP delivered performance and patient outcomes. CDSS can improve adherence to guidelines and enhance patient care. Triaging of emergency patients, glycaemic control, and screening of malnutrition and obesity all represent appropriate targets for CDSS. These conclusions require cautious interpretation: they are based on mainly low-quality studies, with heterogeneous outcomes and indicators.

To improve the quality of studies and consistency of outcomes, future research should satisfy two key requirements. First, system designers and evaluators should consider appropriate implementation theory/models given the planned technology and associated work. Second, study reporting is varied, poor quality and lacking essential detail for implementation; guidelines for conducting and reporting CDSS should be a feature of the publication of findings. This would make synthesis easier and more informative. Guidelines for CDSS reporting in general already exist, it is difficult to conceive why they cannot be applied to nursing and AHP-focused CDSS.[56 57]

FUNDING: The review is funded by the National Institute for Health Research Health Services Delivery and Research programme [award number: NIHR127926]. The views expressed in this publication are those of the authors and not necessarily those of the NHS, the NIHR or the Department of Health.

PATIENT AND PUBLIC INVOLVEMENT: The initial impetus for the bid came from discussions with Governors and service user representatives from Leeds Partnership NHS Foundation Trust. CT was a Non-Executive Director involved with the introduction of technology to help assessment by community mental health nurses. Because end-users of the review will be professionals and commissioners as well as patients and the public, we have set up an advisory group composed of people with specialist knowledge of digital health in a UK NHS context. One of our co-authors, Alison Ledward, is a public member of the UK NIHR HS&DR Researcher-Led Panel and has helped to shape our protocol. She has helped to ensure we maintain our focus on the effects of CDSS on patient

For peer review only - http://bmjopen.bmj.com/site/about/guidelines.xhtml

outcomes and experiences and determining whether CDSS help nurses and AHPs make better decisions for patients.

AUTHOR CONTRIBUTIONS: AK, AL, CT, DA, HY, KB and RR contributed to conception of the review. DA conducted online database searches. AK, CT, HK, HY, RR, SS and TM contributed to titles and abstracts screening. CT and TM contributed to full text screening, quality assessment and data extraction. TM analysed and summarised data as well as produced the first draft of the manuscript. All authors have been involved in revising the work for important intellectual content and have approved the final version for publication.

COMPETING INTERESTS: None declared

PATIENTS CONSENT FOR PUBLICATION: Not required

ETHICS APPROVAL: Not required

For peer review only

REFERENCES

1. Murdoch J, Barnes R, Pooler J, et al. The impact of using computer decision-support software in primary care nurse-led telephone triage: interactional dilemmas and conversational consequences. *Social Science & Medicine* 2015;126:36-47.
2. Anderson JA, Willson P. Clinical Decision Support Systems in Nursing: Synthesis of the Science for Evidence-Based Practice. 2008;26(3):151-58. doi: 10.1097/01.NCN.0000304783.72811.8e
3. Cresswell K, Majeed A, Bates DW, et al. Computerised decision support systems for healthcare professionals: an interpretative review. *Informatics in primary care* 2012;20(2):115-28. [published Online First: 2012/01/01]
4. Bonney W. Impacts and risks of adopting clinical decision support systems. *Efficient Decision Support Systems: Practice and Challenges in Biomedical Related Domain* 2011:21-30.
5. Langhorne P, Ramachandra S. Organised inpatient (stroke unit) care for stroke: network meta-analysis. *Cochrane Database of Systematic Reviews* 2020(4) doi: 10.1002/14651858.CD000197.pub4
6. Sintchenko V, Coiera E. Decision complexity affects the extent and type of decision support use. *AMIA Annual Symposium proceedings AMIA Symposium* 2006;2006:724-28.
7. Garg AX, Adhikari NKJ, McDonald H, et al. Effects of Computerized Clinical Decision Support Systems on Practitioner Performance and Patient OutcomesA Systematic Review. *JAMA* 2005;293(10):1223-38. doi: 10.1001/jama.293.10.1223 %J JAMA
8. Bright TJ, Wong A, Dhurjati R, et al. Effect of Clinical Decision-Support Systems: A Systematic Review. *Annals of Internal Medicine* 2012;157(1):29-43. doi: 10.7326/0003-4819-157-1-201207030-00450 %J Annals of Internal Medicine
9. Kwan JL, Lo L, Ferguson J, et al. Computerised clinical decision support systems and absolute improvements in care: meta-analysis of controlled clinical trials. *bmj* 2020;370
10. Moja L, Kwag KH, Lytras T, et al. Effectiveness of computerized decision support systems linked to electronic health records: a systematic review and meta-analysis. *Am J Public Health* 2014;104(12):e12-22. doi: 10.2105/AJPH.2014.302164 [published Online First: 2014/10/17]
11. Randell R, Mitchell N, Dowding D, et al. Effects of computerized decision support systems on nursing performance and patient outcomes: a systematic review. *Journal of Health Services Research & Policy* 2007;12(4):242-51. doi: 10.1258/135581907782101543
12. Centre for Reviews and Dissemination. Systematic Reviews: CRD's Guidance for Undertaking Reviews in Health Care. . York: Centre for Reviews and Dissemination: University of York 2009.
13. Liberati A, Altman DG, Tetzlaff J, et al. The PRISMA statement for reporting systematic reviews and meta-analyses of studies that evaluate health care interventions: explanation and elaboration. *Annals of internal medicine* 2009;151(4):W-65-W-94.
14. Thompson C, Randell R, Keenan AM, et al. Effects of computerised clinical decision support systems (CDSS) on nursing and allied health professional performance and patient outcomes: a systematic review and user contextualisation.: PROSPERO; 2019 [cited background. Available from: https://www.crd.york.ac.uk/prospero/display_record.php?ID=CRD42019147773.
15. Cochrane Effective Practice Organisation of Care (EPOC). EPOC Resources for review authors: What study designs can be considered for inclusion in an EPOC review and what should that be called? 2017 [cited background. Available from: http://epoc.cochrane.org/resources/epoc-resources-review-authors accessed April 15, 2020.
16. Cochrane Effective Practice and Organisation of Care (EPOC). Data collection form. EPOC resources for review authors 2017 [cited background. Available from: https://epoc.cochrane.org/resources/epoc-resources-review-authors accessed March 30,2020.

For peer review only - http://bmjopen.bmj.com/site/about/guidelines.xhtml

17. Higgins JPT TJ, Chandler J, Cumpston M, Li T, Page MJ, Welch VA (editors). Cochrane Handbook for Systematic Reviews of Interventions version 6.0 (updated July 2019): Cochrane 2019.
18. Cochrane Effective Practice and Organisation of Care (EPOC). Suggested risk of bias criteria for EPOC reviews. EPOC resources for review authors 2017 [cited background. Available from: https://epoc.cochrane.org/resources/epoc-resources-review-authors.
19. Borenstein M, Hedges L, Higgins J, et al. When does it make sense to perform a meta-analysis? Introduction to Meta-Analysis. Chester, UK: John Wiley & Sons Ltd 2009.
20. Beeckman D, Clays E, Van Hecke A, et al. A multi-faceted tailored strategy to implement an electronic clinical decision support system for pressure ulcer prevention in nursing homes: a two-armed randomized controlled trial. *Int J Nurs Stud* 2013;50(4):475-86. doi: https://dx.doi.org/10.1016/j.ijnurstu.2012.09.007
21. Bennett P, Hardiker N. A Quantitative Study Investigating the Effects of Computerised Clinical Decision Support in the Emergency Department. *Studies in health technology and informatics* 2016;225:53-57.
22. Blaha J, Kopecky P, Matias M, et al. Comparison of Three Protocols for Tight Glycemic Control in Cardiac Surgery Patients. *Diabetes Care* 2009;32(5):757. doi: 10.2337/dc08-1851
23. Byrne CM. Impact of prospective computerized clinical decision support information and targeted assistance on nursing home resident outcomes. State University of New York at Albany, 2005.
24. Canbolat O, Kapucu S, Kilickaya O. Comparison of Routine and Computer-Guided Glucose Management for Glycemic Control in Critically Ill Patients. *Critical Care Nurse* 2019;39(4):20-27. doi: 10.4037/ccn2019431
25. Cavalcanti AB, Silva E, Pereira AJ, et al. A randomized controlled trial comparing a computer-assisted insulin infusion protocol with a strict and a conventional protocol for glucose control in critically ill patients. *Journal of Critical Care* 2009;24(3):371-78. doi: https://doi.org/10.1016/j.jcrc.2009.05.005
26. Cleveringa FGW, Gorter KJ, Van Donk MD, et al. Combined task delegation, computerized decision support, and feedback improve cardiovascular risk for type 2 diabetic patients. *Diabetes Care* 2008;31(12):2273-75.
27. Cleveringa FGW, Welsing PMJ, Van Den Donk M, et al. Cost-effectiveness of the diabetes care protocol, a multifaceted computerized decision support diabetes management intervention that reduces cardiovascular risk. *Diabetes Care* 2010;33(2):258-63.
28. Cortez S. Measuring active clinical decision support influence on nursing research utilization. University of Phoenix, 2014.
29. Dalaba MA, Akweongo P, Aborigo RA, et al. Cost-effectiveness of clinical decision support system in improving maternal health care in Ghana. *PLoS ONE* 2015;10(5):e0125920. doi: https://dx.doi.org/10.1371/journal.pone.0125920
30. Dowding DW, Turley M, Garrido T. The impact of an electronic health record on nurse sensitive patient outcomes: an interrupted time series analysis. *J Am Med Inform Assoc* 2012;19(4):615-20. doi: https://dx.doi.org/10.1136/amiajnl-2011-000504
31. Duclos A, Touzet S, Restier L, et al. Implementation of a computerized system in pediatric wards to improve nutritional care: a cluster randomized trial. *European journal of clinical nutrition* 2015;69(7):769-75. doi: 10.1038/ejcn.2014.288
32. Dumont C, Bourguignon C. Effect of a computerized insulin dose calculator on the process of glycemic control. *Am J Crit Care* 2012;21(2):106-15. doi: https://dx.doi.org/10.4037/ajcc2012956
33. Fitzmaurice DA, Hobbs FD, Murray ET, et al. Oral anticoagulation management in primary care with the use of computerized decision support and near-patient testing: a randomized, controlled trial. *Archives of Internal Medicine* 2000;160(15):2343-8.

For peer review only - http://bmjopen.bmj.com/site/about/guidelines.xhtml

34. Forberg U, Unbeck M, Wallin L, et al. Effects of computer reminders on complications of peripheral venous catheters and nurses' adherence to a guideline in paediatric care-a cluster randomised study. *Implementation Science* 2016;11 doi: 10.1186/s13012-016-0375-9
35. Fossum M, Alexander GL, Ehnfors M, et al. Effects of a computerized decision support system on pressure ulcers and malnutrition in nursing homes for the elderly. *Int J Med Inf* 2011;80(9):607-17. doi: https://dx.doi.org/10.1016/j.ijmedinf.2011.06.009
36. Geurts D, de Vos-Kerkhof E, Polinder S, et al. Implementation of clinical decision support in young children with acute gastroenteritis: a randomized controlled trial at the emergency department. *European Journal of Pediatrics* 2017;176(2):173-81.
37. Hovorka R, Kremen J, Blaha J, et al. Blood Glucose Control by a Model Predictive Control Algorithm with Variable Sampling Rate Versus a Routine Glucose Management Protocol in Cardiac Surgery Patients: A Randomized Controlled Trial. *The Journal of Clinical Endocrinology & Metabolism* 2007;92(8):2960-64. doi: 10.1210/jc.2007-0434
38. Kroth PJ, Dexter PR, Overhage JM, et al. A computerized decision support system improves the accuracy of temperature capture from nursing personnel at the bedside. *Amia Annual symposium proceedings AMIA symposium* 2006:444-48.
39. Lattimer V, George S, Thompson F, et al. Safety and effectiveness of nurse telephone consultation in out of hours primary care: Randomised controlled trial. *British Medical Journal* 1998;317(7165):1054-59.
40. Lattimer V, Sassi F, George S, et al. Cost analysis of nurse telephone consultation in out of hours primary care: evidence from a randomised controlled trial. *BMJ* 2000;320(7241):1053-7.
41. Lee NJ, Chen ES, Currie LM, et al. The effect of a mobile clinical decision support system on the diagnosis of obesity and overweight in acute and primary care encounters. *ANS Adv Nurs Sci* 2009;32(3):211-21. doi: https://dx.doi.org/10.1097/ANS.0b013e3181b0d6bf
42. Mann EA, Jones JA, Wolf SE, et al. Computer decision support software safely improves glycemic control in the burn intensive care unit: A randomized controlled clinical study. *Journal of Burn Care and Research* 2011;32(2):246-55.
43. McDonald MV, Feldman PH, Barron-Vaya Y, et al. Outcomes of clinical decision support (CDS) and correlates of CDS use for home care patients with high medication regimen complexity: a randomized trial. *Journal of Evaluation in Clinical Practice* 2017;22(1):10-19.
44. Plank J, Blaha J, Cordingley J, et al. Multicentric, Randomized, Controlled Trial to Evaluate Blood Glucose Control by the Model Predictive Control Algorithm Versus Routine Glucose Management Protocols in Intensive Care Unit Patients. *Diabetes Care* 2006;29(2):271. doi: 10.2337/diacare.29.02.06.dc05-1689
45. Rood E, Bosman RJ, van der Spoel JI, et al. Use of a Computerized Guideline for Glucose Regulation in the Intensive Care Unit Improved Both Guideline Adherence and Glucose Regulation. *Journal of the American Medical Informatics Association* 2005;12(2):172-80. doi: 10.1197/jamia.M1598
46. Roukema J, Steyerberg EW, van der Lei J, et al. Randomized trial of a clinical decision support system: impact on the management of children with fever without apparent source. *Journal of the american medical informatics association : JAMIA* 2008;15(1):107-13. doi: 10.1197/jamia.M2164
47. Sassen B, Kok G, Schepers J, et al. Supporting health care professionals to improve the processes of shared decision making and self-management in a web-based intervention: randomized controlled trial. *Journal of medical Internet research* 2014;16(10):e211.
48. Snooks HA, Carter B, Dale J, et al. Support and Assessment for Fall Emergency Referrals (SAFER 1): cluster randomised trial of computerised clinical decision support for paramedics. *PLoS ONE* 2014;9(9):e106436. doi: https://dx.doi.org/10.1371/journal.pone.0106436
49. Vadher BD, Patterson DL, Leaning M. Comparison of oral anticoagulant control by a nurse-practitioner using a computer decision-support system with that by clinicians. *Clinical and laboratory haematology* 1997;19(3):203-07.

For peer review only - http://bmjopen.bmj.com/site/about/guidelines.xhtml

50. Wells B. Implementation of Computerised Clinical Decision Support (CCDS) in a Prehospital Setting: Processes of Adoption and Impact on Paramedic Role and Practice. Swansea University (United Kingdom), 2013.
51. Dykes PC, Carroll DL, Hurley A, et al. Fall prevention in acute care hospitals: a randomized trial. *Jama* 2010;304(17):1912-18.
52. Paulsen MM, Paur I, Gjestland J, et al. Effects of using the MyFood decision support system on hospitalized patients' nutritional status and treatment: A randomized controlled trial. *Clinical Nutrition* 2020;39(12):3607-17.
53. Lv S, Ye X, Wang Z, et al. A randomized controlled trial of a mobile application-assisted nurse-led model used to improve treatment outcomes in children with asthma. *Journal of advanced nursing* 2019;75(11):3058-67.
54. Dykes PC, Burns Z, Adelman J, et al. Evaluation of a patient-centered fall-prevention tool kit to reduce falls and injuries: a nonrandomized controlled trial. *JAMA network open* 2020;3(11):e2025889-e89.
55. National Institute for Health and Clinical Excellence (NICE). Guide to the methods of technology appraisal London2013 [Available from: https://www.nice.org.uk/process/pmg9/chapter/the-appraisal-of-the-evidence-and-structured-decision-making accessed November 18,2020.
56. Kawamoto K, McDonald CJ. Designing, conducting, and reporting clinical decision support studies: recommendations and call to action. *Annals of Internal Medicine* 2020;172(11_Supplement):S101-S09. doi: https://doi.org/10.7326/M19-0875
57. National Health Services (NHS). Digital Health: NHSX launches assessment criteria for digital health tools 2020 [cited discussion. Available from: https://www.digitalhealth.net/2020/10/nhsx-launches-assessment-criteria-for-digital-health-tools/ accessed October 29, 2020.

For peer review only

For peer review only - http://bmjopen.bmj.com/site/about/guidelines.xhtml

SUPPLEMENTARY MATERIAL LIST

Supplementary Table 1: Ovid MEDLINE(R) ALL, 1946 to February 12, 2021 Search Strategy

Supplementary Table 2: Risk of Bias assessment justifications using Effective Practice Organisation of Care (EPOC)'s tool

Supplementary Table 3: Summary of patient care process results

Supplementary Table 4 Summary of patient care outcomes results

Supplementary Table 5: Summary of Health professionals' knowledge, beliefs and behaviour results

Supplementary Table 6: Summary of adverse events results

Supplementary Table 7: Summary of economic costs and consequences results

For peer review only

Supplementary Table 1: Ovid MEDLINE(R) ALL, 1946 to February 12, 2021 Search Strategy

----

1 exp Decision Making/ (207895)

2 decision support techniques/ (20911)

3 (decision* adj2 making).ti,ab,kf. (159754)

4 (decision* adj2 support*).ti,ab,kf. (24230)

5 (decision* adj2 aid*).ti,ab,kf. (6501)

6 or/1-5 (354546)

7 exp Computers/ (79322)

8 exp information systems/ (238259)

9 exp Informatics/ (537355)

10 Internet/ (74916)

11 Software/ (112580)

12 Cell Phone/ (8821)

13 Mobile Applications/ (6962)

14 exp Telemedicine/ (32559)

15 Medical Records Systems, Computerized/ (19076)

16 exp Electronic Health Records/ (21793)

17 computer*.ti,ab,kf. (313610)

18 electronic*.ti,ab,kf. (291368)

19 (internet or web or online or on-line).ti,ab,kf. (310071)

20 (software or computer program*).ti,ab,kf. (193359)

21 (automate* or automation).ti,ab,kf. (136436)

22 (pda or pdas).ti,ab,kf. (13229)

23 personal digital assistant*.ti,ab,kf. (1012)

24 (app or apps).ti,ab,kf. (31717)

25 (application* adj2 mobile*).ti,ab,kf. (4834)

26 (iPad* or iPhone* or smartphone* or smart phone* or smart device* or mobile phone or android phone* or cellphone* or cell phone*).ti,ab,kf. (26450)

27 (tablet adj2 (pc or device* or comput*)).ti,ab,kf. (1603)

28 ((hand held or handheld) adj2 (pc or device* or comput*)).ti,ab,kf. (2669)

29 (telehealth or telecare or telemedicine or ehealth or mhealth).ti,ab,kf. (29130)

30 or/7-29 (1674343)

31 6 and 30 (66042)

32 exp Decision Making, Computer-Assisted/ (149528)

33 Decision Support Systems, Clinical/ (8302)

34 (computer assisted adj2 (decision* or diagnos* or therap* or support or treatment? or management)).ti,ab,kf. (1545)

35 (computer aided adj2 (decision* or diagnos* or therap* or support or treatment? or management)).ti,ab,kf. (3921)

36 (decision adj2 support adj2 (system* or tool*)).ti,ab,kf. (9917)

37 (decision making adj2 (system* or tool*)).ti,ab,kf. (2560)

38 Expert Systems/ (3420)

39 (expert adj2 system*).ti,ab,kf. (3613)

40 Reminder Systems/ (3568)

41 ((computer* or electronic* or CDSS) adj2 (reminder* or alert*)).ti,ab,kf. (1210)

42 ((medication or medicine or treatment or therapy) adj2 (reminder* or alert*)).ti,ab,kf. (857)

43 reminder system*.ti,ab,kf. (875)

44 Medical Order Entry Systems/ (2303)

45 ((computer* or electronic*) adj2 order entry).ti,ab,kf. (1874)

46 (computer adj2 decision support*).ti,ab. (412)

47 CPOE.ti,ab,kf. (1139)

48 or/32-47 (177952)

49 31 or 48 [all computerised clinical decision support systems terms] (228840)

50 Allied Health Personnel/ (11925)

51 Allied Health Occupations/ (587)

52 Physical Therapist Assistants/ (16)

53 Physical Therapy Specialty/ (2889)

54 Speech-Language Pathology/ (3172)

55 Occupational Therapy/ (13482)

56 Nutritionists/ (1290)

For peer review only

57 dietetics/ (7837)

58 Anesthesiologists/ (1163)

59 podiatry/ (2273)

60 exp Osteopaths/ (321)

61 osteopathic physicians/ (321)

62 anesthesiologist*.ti,ab,kf. (22810)

63 podiatrist*.ti,ab,kf. (910)

64 prosthetist*.ti,ab,kf. (397)

65 chiropodist*.ti,ab,kf. (132)

66 orthoptist*.ti,ab,kf. (319)

67 orthotist*.ti,ab,kf. (220)

68 osteopath*.ti,ab,kf. (5983)

69 radiographer*.ti,ab,kf. (1803)

70 art therapist*.ti,ab,kf. (89)

71 drama therapist*.ti,ab,kf. (3)

72 music therapist*.ti,ab,kf. (368)

73 (allied adj2 health adj2 (profession* or worker* or personnel or occupation* or staff)).ti,ab,kf. (3421)

74 ((physical or occupational or language or speech or physio*) adj2 therap*).ti,ab,kf. (50227)

75 physiotherapist*.ti,ab,kf. (8544)

76 dietetic*.ti,ab,kf. (9828)

77 dietitian*.ti,ab,kf. (6580)

78 nutritionist*.ti,ab,kf. (3020)

79 Patient care team/ (66483)

80 ((multidisciplinary or multi-disciplinary or multiprofessional or multi-professional or interdisciplinary or interprofessional) adj2 team*).ti,ab,kf. (32126)

81 Emergency Medical Technicians/ (5756)

82 Emergency Medical Services/ (43736)

83 Ambulances/ (6210)

84 Air Ambulances/ (2874)

85 paramedic*.ti,ab,kf. (8537)

For peer review only

86 HEMS.ti,ab,kf. (767)

87 ems.ti,ab,kf. (13017)

88 emt.ti,ab,kf. (25232)

89 prehospital.ti,ab,kf. (13136)

90 pre-hospital.ti,ab,kf. (4836)

91 first responder*.ti,ab,kf. (2449)

92 emergency medical technician*.ti,ab,kf. (1168)

93 emergency services.ti,ab,kf. (4115)

94 ambulance*.ti,ab,kf. (11269)

95 field triage.ti,ab,kf. (275)

96 out-of-hospital.ti,ab,kf. (11317)

97 (nurse or nurses or nursing).ti,ab,kf. (462330)

98 exp nurses/ (89638)

99 exp nursing staff/ (67063)

100 Midwifery/ (19460)

101 (midwif* or midwiv*).ti,ab,kf. (25895)

102 or/50-101 [allied health professionals or nurses or midwives] (836031)

103 49 and 102 [all CDSS and allied health professionals or nurses or midwives] (9549)

For peer review only

Supplementary Table 2: Risk of Bias assessment justifications using Effective Practice Organisation of Care (EPOC)'s tool

1. Randomised controlled trials, non-randomised trials and controlled before-after studies

Random sequence generation	Allocation concealment	Baseline outcome measurements similar	Baseline characteristics similar	Incomplete outcome data	Knowledge of the allocated interventions adequately prevented during the study	Protection against contamination	Selective outcome reporting	Other bias	Overall bias score
**Beeckman et al, 2013**									
"Simple randomisation was used to allocate nurses and patients"	Nurses and residents knew their allocated group	Reported baseline outcomes are broadly similar	Baseline characteristics balanced/similar	No information if there was a problem of missing data or ways of handling it, if any	Assessors were not blinded	Intervention was allocated nursing homes, not individual patients	All relevant outcomes in the methods section are reported in the results section	There is no evidence of other risk of biases	**High**
**Blaha et al, 2009**									
Not specified in paper.	Not specified in paper.	No significant differences in glucose at baseline	Although reported for patients, baseline characteristics of nurses is not reported in text or tables.	Only 11 of 120 patients missing (9%)	The outcomes are objective.	Professionals were allocated within a clinic or practice and it is possible that communication between the two groups could have occurred	All relevant outcomes in the methods section are reported in the results section.	There is no evidence of other risk of biases.	**Unclear**
**Byrne,2005**									
Controlled before-after study.	Controlled before-after study.	Models adjusted for covariates.	No report of baseline characteristics of patients or Nurses involved.	Not specified in the paper.	Not specified in the paper.	Unit of allocation was the nursing home	All relevant outcomes in the methods section are reported in the results section.	Multiple comparison	**High**
**Canbolat et al,2019 (NRCT)**									

Random sequence generation	Allocation concealment	Baseline outcome measurements similar	Baseline characteristics similar	Incomplete outcome data	Knowledge of the allocated interventions adequately prevented during the study	Protection against contamination	Selective outcome reporting	Other bias	Overall bias score
Is Non-randomised trial.	It is an open label study.	No baseline measure of outcomes reported.	No baseline information reported about the providers (Nurses); difference baseline characteristics patients present	Not specified in the paper.	Not specified in the paper.	There was no randomisation; control and intervention groups were from the same clinic. Therefore, it is highly likely that control group could have received intervention	All relevant outcomes are reported in the results section.	No baseline (pre-intervention) outcomes data available so difficult to judge.	**High**
**Cavalcanti et al, 209**									
'Random numbers were generated by computer.'	'Allocation was by centres at the start of the study.'	No baseline measure of outcomes reported in the paper.	Clinically significant differences in patients at baseline; no baseline information about HPs.	Outcomes reported were based on all participants (complete data).	Not specified in the paper.	Not specified in the paper.	All relevant outcomes in the methods section are reported in the results section.	No evidence of other sources of bias.	**High**
**Cleveringa et al,2008**									
Block randomisation by practices and Nurses.	Unit of allocation was by practice.	Baseline outcomes were largely similar among the intervention and control groups.	Clinically significant differences in patients at baseline; no baseline information about HPs.	'Values carried forward method' was used but not ideal method.	Not specified in the paper.	Allocation unit was practice so unlikely that the control group received an intervention.	All relevant outcomes discussed in the objective are reported.	No evidence of other risk of biases.	**High**

Random sequence generation	Allocation concealment	Baseline outcome measurements similar	Baseline characteristics similar	Incomplete outcome data	Knowledge of the allocated interventions adequately prevented during the study	Protection against contamination	Selective outcome reporting	Other bias	Overall bias score
**Cleveringa et al,2010**									
Not specified in the paper.	Unit of allocation was primary care practice.	Baseline outcome measurements are largely similar.	There is no report of baseline characteristics of Nurses in text or tables.	Use of electronic health records	Not specified in the paper.	Allocation was by primary care practices so unlikely that control group received intervention.	All relevant outcomes set out in the objective were reported.	No evidence of other risk of biases.	**High**
**Cortez, 2014**									
Not specified in the paper.	Allocation was based on clinic and nurses.	Outcome measurements were different among the two groups	Baseline characteristics were largely similar in both groups.	Use of electronic health records	'The study participants (nurses) did not know about the other group's usage of CDSS at the start and during the study.'	Nurses in the intervention group did not know about or receive CDSS during study.	All relevant outcomes in the methods section are reported in the results section.	No evidence of other risk of biases.	**High**
**Dalaba et al, 2015**									
A controlled before-after study.	A controlled before-after study.	Baseline outcome measurements were significantly different.	No report of baseline characteristics of HPs in text or tables	Not specified in the paper.	Not specified in the paper.	Comparison groups were in different districts.	All outcomes mentioned in the methods section have been reported.	No indication of other biases.	**High**
**Duclos et al,2015**									
Randomisation computer generated centrally.	Allocation was by department at the start of the study.	Baseline outcome measures appear to be	Only aggregated baseline characteristics of children for	Medical records were used.	Not specified in the paper.	Not specified in the paper.	All relevant outcomes in the methods section are reported in the results section.	No indication of other biases.	**High**

Random sequence generation	Allocation concealment	Baseline outcome measurements similar	Baseline characteristics similar	Incomplete outcome data	Knowledge of the allocated interventions adequately prevented during the study	Protection against contamination	Selective outcome reporting	Other bias	Overall bias score
		different and were not adjusted for during analysis.	the intervention and control groups; and, no report about the HP participants' baseline characteristics in tables or text.						
**Dumont et al,2012**									
Simple randomisation used	Randomisation was achieved by a Nurse choosing unmarked sealed envelope	No baseline measure of outcome reported.	Patient characteristics reported and largely similar, but report on HP were presented as aggregated.	Not specified in the paper.	Not specified in the paper.	Nurses were allocated within a clinic and it is possible that communication between intervention and control nurse could have occurred.	All outcomes in methods section were reported.	Performance bias risk from knowledge of cases, protocols and contamination highly likely.	**High**
**Dykes et al, 2009**									
Not specified in the paper	Allocation was by unit at the start of the study	Baseline outcome measurements are largely similar.	Patient characteristics were similar, but no information on HPs.	Medical records were used.	Study noted as open-label design in the protocol; and, intervention and control units in one hospital.	Contamination of information highly likely; patients rather than professionals were randomised	All outcomes in methods section were reported.	No indication of other biases.	**High**
**Fitzmaurice et al, 2000**									

Random sequence generation	Allocation concealment	Baseline outcome measurements similar	Baseline characteristics similar	Incomplete outcome data	Knowledge of the allocated interventions adequately prevented during the study	Protection against contamination	Selective outcome reporting	Other bias	Overall bias score
'Randomisation was computer generated.'	Not specified in the paper	Baseline outcome measurements are largely similar.	There is no report of baseline characteristics of HPs in text or tables	Use of medical records.	Outcomes are objective.	Groups in same practice—possibility of communication between health professionals	All relevant outcomes in the introduction/methods section are reported in the results section.	No evidence of other risk of biases.	**High**
**Forberg et al,2016**									
'A simple draw from the list by a third person.'	Not specified in the paper	Baseline measure of outcomes appear to be largely similar.	Baseline characteristics of the intervention and control groups are similar.	Missing outcomes is very minimal (<2%).	Not specified in the paper.	Not clear that nurses did not swap between units within the same hospital.	All relevant outcomes in the methods section are reported in the results section.	No evidence of other risk of biases.	**High**
**Fossum et al,2011**									
Controlled before-after study	Controlled before-after study	Baseline outcome measurements are largely similar.	Although reported for patients, baseline characteristics of providers was not reported in text or tables.	Use medical records.	Not specified in the paper.	Allocation was by nursing homes and is unlikely that control group received intervention.	All relevant outcomes in the methods section are reported in the results section.	No evidence of other risk of biases.	**High**
**Geurts et al, 2016**									
'Computer generated randomisation was used.'	'Centralised randomisation scheme used.'	No baseline measure of outcome in the paper.	Baseline characteristics are largely similar among the two groups.	Medical records used.	'Nurses were blinded for the contribution of predictors on the risk score.'	Patient based randomisation; a high possibility. Intra clinician and inter linician	All relevant outcomes in the methods section are reported in the results section.	Question about representativeness of final study sample as 75% of eligible kids not randomised as	**High**

Random sequence generation	Allocation concealment	Baseline outcome measurements similar	Baseline characteristics similar	Incomplete outcome data	Knowledge of the allocated interventions adequately prevented during the study	Protection against contamination	Selective outcome reporting	Other bias	Overall bias score
						contamination highly possible.		professional or parents non-compliant.	
**Hovorka et al, 2007**									
'randomisation based on computer algorithm'	Centralised randomisation scheme was used.	No baseline measure of outcome reported in the paper.	Although some report about patients, no report of baseline characteristics about HP participants in text or tables.	Not specified in the paper.	The outcomes were objective.	patients based randomisation; same clinicians involved in standard and intervention arms	All relevant outcomes in the methods section are reported in the results section.	No evidence of other risk of biases.	**High**
**Kroth et al, 2006**									
'Randomisation using coin flip.'	Not specified in the paper.	No baseline measure of outcome.	There is no detailed report of characteristics in text or tables.	Consecutive [medical] records used.	objective outcome	Randomisation was for patients and nurses. Nurses in the control group did not receive reminders.	All relevant outcomes in the methods section are reported in the results section.	No evidence of other risk of biases.	**High**
**Lattimer et al, 1998**									
'A random number generator pocket calculator (Hewlett Packard 21s) used'	Unit of allocation was by team and allocation was performed on all units at the start of the study.	No baseline measure of outcome reported.	Some about patients, but no report of baseline characteristics HPs in text or tables.	Not specified in the paper.	Use of medical records.	Health professionals in the intervention (Nurses) and control (Doctors) were different.	All relevant outcomes in the methods section are reported in the results section.	No evidence of other risk of biases.	**Unclear**

Random sequence generation	Allocation concealment	Baseline outcome measurements similar	Baseline characteristics similar	Incomplete outcome data	Knowledge of the allocated interventions adequately prevented during the study	Protection against contamination	Selective outcome reporting	Other bias	Overall bias score
**Lattimer et al,2000**									
Not specified in the paper.	Not specified in the paper.	Not specified in the paper.	There is no detailed report of characteristics in text or tables	Not specified in the paper.	Use of medical records.	Health professionals in the intervention (Nurses) and control (Doctors) were different.	All relevant outcomes in the methods section are reported in the results section.	No evidence of other risk of biases.	**Unclear**
**Lee et al, 2009**									
Not specified in the paper.	Not specified in the paper.	Although weight and BMI data were recorded, no data on the outcome measurements.	Reported for patients, but no report on providers in text or tables.	Not specified in the paper.	Not specified in the paper.	Patients based randomisation so it is likely that the control group received the intervention.	All relevant outcomes in the methods section are reported in the results section.	No evidence of other risk of biases.	**High**
**Lv et al, 2019**									
Not specified in the paper.	Not specified in the paper.	Not specified in the paper.	Reported for patients, but no report on providers in text or tables.	Not specified in the paper.	Not specified in the paper.	Patients based randomisation; Patient based randomisation; same clinicians involved in both arms.	All relevant outcomes in the methods section are reported in the results section.	No evidence of other risk of biases.	**High**
**Mann et al,2011**									
Computer generated sequence was used.	Not specified in the paper.	Baseline measure of outcome not reported.	No baseline characteristics of HPs in text or tables were found.	Not clear from the paper.	A cross-over study; not specified in the paper.	Acrossover trial with only patients rather than professionals randomised.	All relevant outcomes in the methods section are reported in the results section.	No evidence of other risk of biases.	**High**

Random sequence generation	Allocation concealment	Baseline outcome measurements similar	Baseline characteristics similar	Incomplete outcome data	Knowledge of the allocated interventions adequately prevented during the study	Protection against contamination	Selective outcome reporting	Other bias	Overall bias score
**McDonald et al, 2017**									
Automated block randomisation was used.	Automated block randomisation schema was used	Not specified in the paper.	Baseline characteristics were largely similar.	Possible medical records use.	Assessor was not blinded.	Both intervention and control nurses were in one organisation and it is possible that communication between them could have occurred	All relevant outcomes in the methods section are reported in the results section.	Only 42% of patients who should have had a CDSS applied suggesting that the nurses selectively chose which patients to use it with or selective non adoption	**High**
**Paulson et al, 2020**									
Automated block randomisation was used.	Automated block randomisation schema was used	Reported for patients, but no report on providers in text or tables	Baseline characteristics were largely similar	Only complete case analysis conducted	Outcomes are objective	Both intervention and control nurses were in one organisation and it is possible that communication between them could have occurred	All relevant outcomes in the methods section are reported in the results section.	No evidence of other risk of biases.	**High**
**Plank et al, 2006**									
Not specified in the paper	Not specified in the paper	Blood glucose measured but not intervention group based	Differences in types of surgery and history of diabetes between sites	Use of medical records.	Outcomes are objective.	same units delivering all arms of the trial with same clinicians	All relevant outcomes in the methods section are reported in the results section.	No evidence of other risk of biases.	**High**
**Rood et al, 2005**									

Random sequence generation	Allocation concealment	Baseline outcome measurements similar	Baseline characteristics similar	Incomplete outcome data	Knowledge of the allocated interventions adequately prevented during the study	Protection against contamination	Selective outcome reporting	Other bias	Overall bias score
'Automatic random number generating'	Not specified in the paper	Baseline measure of outcome not reported.	No report of characteristics of HPs in text or tables.	Not specified in the paper.	Not specified in the paper.	Patient based randomisation; same clinicians involved in both arms.	There is no evidence that outcomes were selectively reported.	No evidence of other risk of biases.	**High**
**Roukema et al,2008**									
Randomisation was based on computer algorithm.	'centralised randomisation scheme'	Baseline measure of outcome not reported	No report of characteristics of HPs in text or tables.	Not specified in the paper.	Not specified in the paper.	professionals were allocated within a clinic so hard to see how decision rule training effect not present in the clinicians who were delivering both arms of the trial	All relevant outcomes in the methods section are reported in the results section.	No evidence of other risk of biases.	**High**
**Sassen et al,2014**									
Not specified in the paper.	The unit of allocation was by health professional and allocation was performed on all units at the start of the study	No important differences were present across study groups.	Baseline characteristics of the study and control providers are reported and similar.	Significant proportion participants dropped out and the report is based on the complete case analysis.	Outcomes cannot be assessed blindly.	Participants in the control group did not have a log-in code to access the website (CDSS tool) until post-intervention data were collected.	All relevant outcomes in the methods section are reported in the results section.	No evidence of other risk of biases.	**High**
**Snooks et al, 2014**									

Random sequence generation	Allocation concealment	Baseline outcome measurements similar	Baseline characteristics similar	Incomplete outcome data	Knowledge of the allocated interventions adequately prevented during the study	Protection against contamination	Selective outcome reporting	Other bias	Overall bias score
Randomisation based on computer algorithm.	Random allocation was performed on all units at the start of the study.	No baseline measure of outcome reported.	No report of characteristics in text or tables about the paramedics involved.	Not specified in the paper.	Analyst was blinded.	Intervention and control groups were in separates sites	All relevant outcomes in the methods section are reported in the results section.	No evidence of other risk of biases.	**Unclear**
**Vadher et al, 1997**									
Random tables were used.	Not specified in the paper.	No baseline measure of outcome reported.	Patient baseline characteristics reported; one nurse versus a clinician.	Not specified in the paper.	Outcomes are objectively measured.	Hard to see how same clinicians seeing both arm trial patients didn't pick up something from the CDSS.	All relevant outcomes in the methods section are reported in the results section.	There was only one Nurse participant in the intervention group.	**High**
**Wells,2013**									
Random table was used for randomisation.	Not specified in the paper.	No baseline measure of outcomes reported.	Baseline characteristics are largely similar.	Not specified in the paper.	Outcomes were assessed blindly.	Intervention and control groups in the same site so it is likely that the control group received the intervention.	All relevant outcomes in the methods section are reported in the results section	No evidence of other risk of biases.	**High**

Colour codes: Red, high risk; orange, unclear risk; green, low risk

2. Interrupted time series studies

Author & Year	Risk of bias domains and scores							Overall bias
	Intervention independent of other changes	Shape of the intervention effect pre-specified	Intervention unlikely to affect data collection	Knowledge of the allocated interventions adequately prevented during the study	Incomplete outcome data adequately	Selective outcome reporting	Other bias	
Bennet, 2016	Very long adoption period with no measurement; possible confounding factors not presented/models not adjusted	Data were classified as pre and post-intervention from the point/date of intervention.	Data were collected from the hospital records databases for pre- and post-intervention periods	Not presented in the paper.	Medical records used	All relevant outcomes in the methods section are reported in the results section.	No evidence of other risk of biases.	**High**
Dykes et al,2020	Highly likely the changes in outcome to be influenced by confounders.	Point of analysis is the point of intervention.	Sources and methods of data collection were the same before and after the intervention.	Not presented in the paper.	Medical records used	All relevant outcomes are reported in the results section	No evidence of other risk of biases.	
Dowding et al,2012	Highly likely the changes in outcome to be influenced by confounders.	Point of analysis is the point of intervention.	Sources and methods of data collection were the same before and after the intervention.	Not presented in paper.	Medical records used	All relevant outcomes are reported in the results section.	No evidence of other risk of biases.	**High**

Colour codes: Red, high risk; orange, unclear risk; green, low risk

Supplementary Table 3: Summary of patient care process results

Author & Year	Interventions	Health professionals	patient participants	Outcome measured	Outcome values reported	Change of value within a group[‡]	Risk difference (95% CI)[‡]
**1. Adherence to guidelines**							
Dumont et al,2012	• CDSS use<br>• Paper protocol	Nurses (OA=44)<br>Nurses	141 adults<br>159 adults	Deviations from the protocol, out of 10 (mean (SD))	4 months=0.39(1.0)<br>4 months=3.0(4.3)	-	Mean difference: -2.61 (-4.5 to -0.71)
Forberg et al,2016	• CDSS-use<br>• CDSS non-use	108 Nurses<br>103 Nurses	Not applicable<br>Not applicable	Nurses adherence to guidelines on disinfection of hands	Baseline=97/108<br>3 months =93/105<br>Baseline=96/103<br>3 months=87/102	-1.2%<br>-7.9%	6.7% (4.9 to 8.5)
	• CDSS-use<br>• CDSS non-use			Nurses adherence to guidelines on usage of disposable gloves (n/N)	Baseline=80/108<br>3 months =76/105<br>Baseline=71/103<br>3 months =70/102	-1.7%<br>-0.3%	-1.4% (-2.2 to -0.5)
	• CDSS-use<br>• CDSS non-use			Nurses adherence to guidelines on daily inspection of Peripheral Venous Catheters (PVC) site (n/N)	Baseline=58/108<br>3 months =58/103<br>Baseline=47/102<br>3 months =55/102	2.6%<br>7.8%	-5.2% (-7.1 to -3.3)
Rood et al, 2005	• CDSS-based GL<br>• Paper-based GL	ICU Nurses<br>ICU Nurses	66 adults<br>54 adults	Adherence to Insulin dose Advice (n/N)	10 weeks =1818/2352<br>10 weeks =1667/2597	-<br>-	22% (19 to 25)
	• CDSS-based GL<br>• Paper-based GL	ICU Nurses<br>ICU Nurses	66 adults<br>54 adults	Adherence to the guideline for taking blood samples on time (n/N)	10 weeks =945/2352<br>10 weeks =922/2597	-<br>-	4.7% (2.0 to 7.4)
Vadher et al, 1997	• CDSS<br>• Control	1 Nurse<br>3 trainee Doctors	87 adults<br>90 adults	Dose advice 'acceptance' in patients with therapeutic range 2-3	Post-test =188/214<br>Post-test=145/242	-<br>-	28% (20.4 to 35.5)
	• CDSS<br>• Control	1 Nurse<br>3 trainee Doctors		Dose advice 'acceptance' in patients with therapeutic range 3-4.5 (n/N)	Post-test =160/239<br>Post-test=150/205	-	-6.2% (-14.7 to 2.2)
	• CDSS<br>• Control	1 Nurse<br>3 trainee Doctors		Interval advice 'acceptance' (%) in patients with therapeutic range 2-3	Post-test =170/230<br>Post-test=133/266	-	23.9% (15.6 to 32.2)
	• CDSS<br>• Control	1 Nurse<br>3 trainee Doctors		Interval advice 'acceptance' (%) in patients with therapeutic range 3-4.5	Post-test =129/239<br>Post-test=101/202	-	3.9% (-5.4 to 13.3)
**2. Patient assessment, diagnosis, and treatment practices**							
Bennett et al,	• CDSS use period			Pain assessment	Post-test=97.7%	-	62.7% (59.6 to 65.8)

For peer review only

2016	• CDSS non use				Pre-test=35%		
	• CDSS use			IV antibiotics in 1hr for sepsis	Post-test=5.6%	-	-5.9% (-8.3 to -3.5)
	• CDSS non use				Pre-test=11.5%		
Duclos et al,2015	• CDSS	Dieticians	667 children	Investigation of malnutrition aetiology	Post-test=284/667	-	21.2% (15.9 to 26.5)
	• Usual care	Dieticians	477 children		Post-test=102/477		
	• CDSS	Dieticians	667 children	Managed by a dietitian	Post-test=305/667	-	12% (6.3 to 17.7)
	• Usual care	Dieticians	477 children		Post-test=161/477		
	• CDSS	Dieticians	667 children	prescribed refeeding protocol	Post-test=230/667	-	-4.5% (-10.2 to 1.2)
	• Usual care	Dieticians	477 children		Post-test=186/477		
Geurts et al, 2017	• CDSS	Nurses	113 children	Patient consultation time(min)-median (IQR)	Post-test =136(108)	-	3 min
	• Usual care	Nurses	109 children		Post-test =133(92)		
	• CDSS	Nurses	113 children	Electrolytes level test	Post-test =15/113	-	-7.8% (-17.7 to 2.1)
	• Usual care	Nurses	109 children		Post-test =23/109		
	• CDSS	Nurses	113 children	Acid-base balance test	Post-test =13/113	-	-3.2% (-12.1 to 5.7)
	• Usual care		109 children		Post-test =16/109		
	• CDSS	Nurses	113 children	Oral Rehydration Solution (nasogastric tube)	Post-test =17/113	-	6.7% (-1.6 to 15.2)
	• Usual care	Nurses	109 children		Post-test =9/109		
	• CDSS	Nurses	113 children	IV rehydration given	Post-test =0/113	-	-1.8% (-4.4 to 0.7)
	• Usual care	Nurses	109 children		Post-test =2/109		
	• CDSS	Nurses	113 children	Other liquid given	Post-test =18/113	-	-11.6% (-22.4 to -0.8)
	• Usual care	Nurses	109 children		Post-test =30/109		
Roukema et al,2008	• CDSS use	Nurses	74 children	Time spent in ED (minutes), median (IQR)	27 months =138 (77)	-	15 minutes
	• Control	Nurses	90 children		27 months =123 (96)		
	• CDSS use	Nurses	74 children	Time spent in ED for lab test (minutes), median (IQR)	27 months =140 (68)	-	-20 minutes
	• Control	Nurses	90 children		27 months =160 (98)		
Snooks et al, 2014	• CDSS	17 Paramedics	436 adults	Mean length of episode of care (minutes)	CDSS Vs control	-	-5.7 min (-38.5 to 27.2)[†]
	• Control	19 Paramedics	343 adults				
Wells,2013	• CDSS	22 paramedics	436 adults	Respiratory rate recorded, %	1 year =405/436	-	-1.2% (-4.7 to 2.2)
	• Control	20 paramedics	341 adults		1 year =321/341		
	• CDSS	22 paramedics	436 adults	Pulse rate recorded	1 year =414/436	-	0.9% (-3.9 to 2.0)
	• Control	20 paramedics	341 adults		1 year =327/341		
	• CDSS	22 paramedics	436 adults	Consciousness recorded	1 year =405/436	-	-5.1% (-7.9 to -2.2)
	• Control	20 paramedics	341 adults		1 year =334/341		
Kroth et al, 2006	• CDSS use	164 Nurses	Not applicable	Proportion of erroneously recorded temperatures	9 months =248/45823	-	-0.8% (-0.9 to -0.6)
	• Control	173 Nurses	Not applicable		9 months =575/44339		
**3. Documenting of events**							
Dowding et	• CDSS use	Nurses		Fall documentation ratio	Post-CDSS use Vs pre-	-	1.4 (0.03 to 73.7)[†]

For peer review only

al,2012	• CDSS non-use	Nurses			CDSS use period		
	• CDSS use<br>• CDSS non-use			Hospital acquired pressure ulcer (HAPU) risk documentation ratio	Post-CDSS use Vs pre-CDSS use period	-	9.1 (1.95 to 42.5)†
Paulson et al, 2020	• CDSS use<br>• Usual care	Nurses<br>Nurses	44 adults<br>50 adults	Documentation of nutritional intake compared to requirements	10 months=37/44<br>10 months=2/50	-	80% (67 to 92)
	• CDSS use<br>• Usual care	Nurses<br>Nurses	44 adults<br>50 adults	Documentation of a nutritional care plan	10 months=31/44<br>10 months=8/50	-	54.4% (37.6 to 71.3)
	• CDSS use<br>• Usual care	Nurses<br>Nurses	44 adults<br>50 adults	Documentation of nutritional treatment	10 months=36/44<br>10 months=29/50	-	23.8% (6 to 41.6)
**4. Patient referrals**							
Snooks et al, 2014	• CDSS<br>• Control	17 Paramedics<br>19 Paramedics	436 adults<br>343 adults	Patients referred to falls service	1 year=42/436<br>1 year=17/343	-	4.7% (1.1 to 8.3)

Note: **‡,** calculated from reported information unless stated otherwise; **†**, as reported by study authors.

For peer review only

Supplementary Table 4: Summary of patient care outcomes results

Author & Year	Interventions	Health professionals	patient participants	Outcome measured	Outcome values reported	Change of value within a group‡	Risk difference (95% CI)‡
**1. Glycaemic control**							
Blaha et al, 2009	• CDSS (eMPC)<br>• Mathias protocol<br>• Bath-protocol	ICU Nurses	40 adults<br>40 adults<br>40 adults	Entire study time in target range (blood glucose)- mmol/l	After 48hrs=46%<br>After 48hrs=38.2%<br>After 48hrs=39.7%	-<br>-	Versus Mathias:<br>7.8% (-13.7 to 29.4)<br>Versus Bath<br>6.3% (-3.9 to 16.5)
	• CDSS (eMPC)<br>• Mathias protocol<br>• Bath-protocol	ICU Nurses	40 adults<br>40 adults<br>40 adults	Entire study mean blood glucose (SE)- mmol/l	Baseline=8.1(0.6)<br>48hrs=5.9(0.2)<br>Baseline=7.9(0.4)<br>48hrs=6.7(0.1)<br>Baseline=8.0(0.2)<br>48hrs=6.5(0.2)	-2.2 mmol/l<br>-1.2 mmol/l<br>-1.5 mmol/l	Versus Mathias:<br>-1 mmol/l<br>Versus Bath:<br>-0.7 mmol/l
Canbolat et al,2019	• CDSS (automated BG control)<br>• Standard protocol	Nurses<br>Physicians	33 adults<br>33 adults	Occasions for BG out of target (120 to 180 mg/dL) range	22 months =2101/5789<br>22 months =2977/5122	-	-21.8% (-23.7 to -20.0)
	• CDSS (automated BG control)<br>• Standard protocol			Occasions for BG out of target range due to insulin treatment	22 months =745/5789<br>22 months =2099/5122	-	-28.1% (-29.7 to -26.5)
Cavalcanti et al, 2009	• CDSS (computer-assisted insulin protocol)<br>• Control (Leuven protocol)<br>• Control (conventional treatment)	ICU Nurses<br>ICU Nurses<br>ICU Nurses	56 adults<br>58 adults<br>53 adults	Mean blood glucose (mmol/dL)	19 months =125<br>19 months =127.1<br>19 months =158.5	-<br>-	Versus Leuven<br>-2.1 mmol/dL<br>Versus conventional<br>-33.5 mmol/dL
	• CDSS (computer-assisted insulin protocol)<br>• Control (Leuven protocol)<br>• Control (conventional treatment)	ICU Nurses<br>ICU Nurses<br>ICU Nurses	56 adults<br>58 adults<br>53 adults	Patients with hypoglycaemia	19 months =12/56<br>19 months =24/58<br>19 months =2/53	-<br>-	Versus Leuven<br>-20% (-36.6 to -3.4)<br>Versus conventional<br>17.6% (5.7 to 29.5)
Cleveringa et al,2008	• CDSS use in diabetic patients<br>• Usual care	Nurses<br>Nurses	1699 adults<br>1692 adults	A1C<7%	Baseline=60.8%<br>1 year=68%<br>Baseline=61.6%<br>1 Year=64.2%	7.2%<br>2.6%	4.6% (2.7 to 6.5)
	• CDSS use in diabetic patients		1699 adults	Systolic BP<140	Baseline=41%<br>1 year=53.9%	12.9%	10.2% (7.9 to 12.5)

For peer review only

	• Usual care		1692 adults		Baseline=39.5%<br>1 year=42.2%	2.7%	
	• CDSS use in diabetic patients		1699 adults	Total cholesterol <4.5mmol/l	Baseline=36.2%<br>1 year=49.0%	10.5%	3.7% (1.2 to 6.2)
	• Usual care		1692 adults		Baseline=38.5%<br>1 year=45.3%	6.8%	
Hovorka et al, 2007	• CDSS (eMPC)	ICU Nurses	30 adults	Proportion in target range (4-6.1 mmol/L)	48 hrs =60.4%	-	32.9% (20.0 to 46.0)
	• Usual care	ICU Nurses	30 adults		48 hrs =27.5%		
	• CDSS (eMPC)			Entire study mean blood glucose (mmol/L) (SD)	48 hrs =6.2 (1.1)	-	-1mmol/L
	• Usual care				48 hrs =7.2 (1.1		
	• CDSS (eMPC)			Time in target range (hours)	48 hrs =14.5		7.9 hrs
	• Usual care				48 hrs =6.6		
Mann et al,2011	• CDSS use	ICU Nurses	18 adults	Occasions glucose range on target (80 to 110 mg/dl)	72 hrs =47%	-	6% (-7.7 to 19.7)
	• Paper protocol	ICU Nurses	18 adults		72 hrs =41%		
	• CDSS use	ICU Nurses		Occasions over target range (over 110 mg/dl)	72 hrs =49%	-	-5% (-18.8 to 8.8)
	• Paper protocol	ICU Nurses			72 hrs =54%		
	• CDSS use			Occasions under target (under 80 mg/dl) range	72 hrs =4.5%	-	-0.3% (-2.1 to 1.5)
	• Paper protocol				72 hrs =4.8%		
Plank et al, 2006	• CDSS (MPC) use	ICU Nurses	Not reported	Occasions within the target glycaemic range (80-110 mg/dl)	48 hrs =52%	-	33% (20.5 to 45.4)
	• Usual care	ICU Nurses	Not reported		48 hrs =19%		
	• CDSS (MPC) use	ICU Nurses	Not reported	Improvement glycaemic control for 48 hours	48 hrs =65%	-	40% (27.4 to 52.6)
	• Usual care	ICU Nurses	Not reported		48 hrs =25%		
	• CDSS (MPC) use		Not reported	Occasions over the target glycaemic range (>110 mg/dl)	48 hrs =46%	-	-31% (-43.7 to -18.2)
	• Usual care		Not reported		48 hrs =77%		
	• CDSS (MPC) use		Not reported	Average glucose (mg/dl)	48 hrs =117mg/dL	-	-14mg/dL
	• Usual care		Not reported		48 hrs =131 mg/dL		
**2. Blood coagulation management**							
Fitzmaurice et al,2000	• CDSS use	Nurses	122 adults	proportion of tests in range	Baseline=223/366<br>1 year =732/1181	1.1%	-1.9% (-3.1 to -0.7)
	• CDSS non-use	Physicians	245 adults		Baseline=264/480<br>1 year =986/1700	3%	
	• CDSS use	Nurses		International Normalised Ratio (INR) Results Within Range Point Prevalence	Baseline=74/118<br>1 year =86/121	8.4%	-2.6% (-5.3 to -0.1)
	• CDSS non-use	Physicians			Baseline=129/244	11%	

For peer review only

					1 year =157/245		
	• CDSS use	Nurses		Time Spent Within INR Target Range	Baseline=64/113<br>1 year =76/110	12%	7% (-0.7 to 14.7)
	• CDSS non-use	Physicians			Baseline=99/174<br>1 year= 143/230	5%	
**3. Antenatal and peripartum care**							
Dalaba et al, 2015	• CDSS use	Nurses	Not reported	Antenatal complications per 1000 attendance	Before=9<br>After =12	0.3%	0.3% (-0.03 to 0.6)
	• CDSS non-use	Nurses	Not reported		Before =16<br>After =16	0%	
	• CDSS use			Delivery complications per 1000 attendances	Before=107<br>After=96	-0.9%	2.4% (1.1 to 3.7)
	• CDSS non-use				Before=133<br>After=100	-3.3%	
**4. Managing patients with chronic co-morbid diseases**							
McDonald et al, 2017	• CDSS use<br>• Usual care	165 Nurses<br>335 Nurses	2550 adults<br>5369 adults	Medication regimen complexity index <24.5	Post-test=158/2550<br>Post-test =333/5369	-	0% (-1.1 to 1.1)
	• CDSS use<br>• Usual care	165 Nurses<br>335 Nurses	2550 adults<br>5369 adults	Emergency room use	Post-test =421/2550<br>Post-test =897/5369	-	-0.2 (-1.9 to 1.6)
	• CDSS use<br>• Usual care	165 Nurses<br>335 Nurses	2550 adults<br>5369 adults	Hospitalisation	Post-test =502/2550<br>Post-test =1133/5369	-	-1.4% (-3.3 to 0.5)
Lv et al, 2019	• CDSS use	Nurses	70 children	Percentage of asthma exacerbations (mean and SD)	Baseline=9(4.3)<br>1 year=3(4.3)	-	-1 % (-3.7 to 1.7)
	• Usual care	Nurses	73 children		Baseline=9 (8.7)<br>1 year=4(4.4)	-	
**5. Outpatient obesity screening**							
Lee et al,2009	• CDSS use<br>• Usual care	13 Nurses<br>16 Nurses	807 adults<br>997 adults	Encounters with obesity related diagnosis	8 months =91/807<br>8 months =10/997	-	10.3% (8.0 to 12.5)
	• CDSS use<br>• Usual care	13 Nurses<br>16 Nurses	807 adults<br>997 adults	Encounters with missed obesity-related diagnosis	8 months =51/208<br>8 months =440/662	-	-41.9% (-48.8 to -35.1)
**6. Fall and pressure ulcer management**							
Beeckman et al, 2013	• CDSS(Pre-vPlan)	65 Nurses and physios	225 adults	Pressure ulcer prevention	Day1=15/58<br>Day120=41/65	37.2%	2.3% (-11.0 to 15.6)
	• Standard protocol	53 Nurses and physios	239 adults		Day1=16/63<br>Day120=41/68	34.9%	
	• CDSS(Pre-vPlan)	65 Nurses and physios	225 adults	Prevalence of pressure	Day 1=34/225<br>Day120=16/225	-8%	-6.3% (-10.2 to -2.4)

For peer review only

	• Standard protocol	53 Nurses and physios	239 adults	ulcer	Day1=39/239<br>Day120=35/239	-1.7%	
Byrne,2005	• CDSS use	89 Nurses	Not reported	Fall rate	Before=0.312<br>After=0.318	0.6%	3.1%
	• CDSS non-use		Not reported		before=0.315<br>After=0.29	-2.5%	
	• CDSS use		Not reported	Pressure ulcer rate	Before=0.085<br>After=0.088	-0.3%	-0.6%
	• CDSS non-use		Not reported		Before=0.091<br>After=0.094	0.3%	
Dowding et al,2012	• CDSS use<br>• CDSS non-use			Fall rate	Post-CDSS use Vs pre-CDSS use period	-	0.91 (0.75 to 1.12)[†]
	• CDSS use<br>• CDSS non-use			HAPU ratio	Post-CDSS use Vs pre-CDSS use period	-	0.47 (0.25 to 0.85)[†]
Dykes et al, 2009	• CDSS use<br>• Usual care	Nurses<br>Nurses	5160 adults<br>5104 adults	Fall rate difference (per 1000 patient days)	CDSS use Vs usual care	-	-1.16 (-2.16 to -0.17)[†]
Dykes et al, 2020	• UDSS use<br>• CDSS non-use	Nurses<br>Nurses	19,283 adults<br>17,948 adults	Fall rate difference (per 1000 patient days)	Post-CDSS use Vs pre-CDSS use period	-	-0.15 (-0.04 to -0.25)[†]
Fossum et al,2011	• CDSS use	Nurses	367 adults	Prevalence of pressure Ulcers	Before=16/167<br>After=23/200	1.9%	4.2% (0.2 to 8.2)
	• CDSS non-use	Nurses	274 adults		Before=17/150<br>After=11/122	-2.3%	
	• CDSS use			Prevalence of malnutrition	Before=45/161<br>After=39/199	-8.3%	-12.4% (-19.1 to -5.7)
	• CDSS non-use				Before=31/148<br>After=30/120	4.1%	
**7. Triaging**							
Bennett et al, 2016	• CDSS use period<br>• CDSS non-use	Nurses<br>Nurses	400 adults<br>400 adults	Correct triage prioritisation	Post-test=85.2%<br>Pre-test=60.5%	-	24.7% (18.8 to 30.6)
Lattimer et al, 1998	• CDSS<br>• Usual care	Nurses<br>Physicians	Not applicable<br>Not applicable	Calls managed with telephone advice from GP	Post-test =1109/7184<br>Post-test =3629/7308	-	-34.2% (-35.6 to -32.8)
	• CDSS<br>• Usual care	Nurses<br>Physicians		Patient attended primary care centre	Post-test =1177/7184<br>Post-test =1934/7308	-	-10% (-11.4 to -8.8)
	• CDSS<br>• Usual care	Nurses<br>Physicians		Patient visited at home by duty GP	Post-test =1317/7184<br>Post-test =1745/7308	-	-5.5% (-6.9 to -4.2)
Lattimer et al, 2000	• CDSS<br>• Usual care	Nurses<br>Physicians		Total admissions within 3 days	1 year =428/7184<br>1 year =507/7308	-	-0.98% (-1.8 to -0.2)
Snooks et al,	• CDSS	Paramedics	436 adults	Patients left at scene	1 year =183/436	-	5.2% (-1.7 to 12.1)

For peer review only

2014	• Control	Paramedics	343 adults	without conveyance to emergency department	1 year =126/343		
	• CDSS<br>• Control		436 adults<br>343 adults	Patients with further emergency admission to hospital or death	1 year=69/436<br>1 year =49/343	-	1.5% (-3.5 to 6.6)
	• CDSS<br>• Control			Patients with ED attendance or emergency admission to hospital or death	1 year =92/436<br>1 year =61/343	-	3.3% (-2.3 to 8.9)
	• CDSS<br>• Control			Patients who reported >1 further fall	1 year =135/236<br>1 year =112/175	-	-6.8% (-16.3 to 2.7)
**8. Quality of life and patients' satisfaction**							
Cleveringa et al,2010	• CDSS use<br>• Usual care			Life-years gained	CDSS Vs usual care	-	0.14 (-0.12 to 0.40)†
	• CDSS use<br>• Usual care			Healthy years (QALYs, discounted)	CDSS Vs usual care	-	0.037 (-0.066 to 0.14)†
Snooks et al, 2014	• CDSS<br>• Control	Paramedics<br>Paramedics	239 adults<br>177 adults	Quality of Life (SF12 MCS), mean (SD)	1 year =41.9(10.3)<br>1 year =42.9(10.9)	-	-1 (-3.1 to 1.1)
	• CDSS<br>• Control	Paramedics<br>Paramedics	239 adults<br>177 adults	Quality of Life (SF12 PCS), mean (SD)	1 year=29(8)<br>1 year=30(8.5)	-	-1 (-2.6 to 0.6)
	• CDSS<br>• Control	Paramedics<br>Paramedics	228 adults<br>165 adults	Patient satisfaction (QC Technical), mean (SD)	1 year =97.8(10.7)<br>1 year=98.2(9.4)	-	-0.4 (-2.4 to 1.6)

Note: **‡,** calculated from reported information unless stated otherwise; **†**, as reported by study authors.

For peer review only

Supplementary Table 5: Summary of Health professionals' knowledge, beliefs and behaviour results

Author & Year	Interventions	Health professionals	patient participants	Outcome measured	Outcome values reported	Change of value within a group‡	Mean or risk difference (95% CI)‡
Beeckman et al, 2013	• CDSS(Pre-vPlan)	65 Nurses and physios	225 adults	Positive knowledge change	Baseline=28/65<br>5 months=26/50	8.9%	6.5% (0.8 to 13.2)
	• Standard protocol	53 Nurses and physios	239 adults		Baseline=21/53<br>5 months=16/38	2.4%	
	• CDSS(Pre-vPlan)	65 Nurses and physios	225 adults	Positive attitude change	Baseline=48/65<br>5 months=42/50	10.2%	12.7% (5.9 to 19.5)
	• Standard protocol	53 Nurses and physios	239 adults		Baseline=39/53<br>5 months=27/38	-2.5%	
Cortez, 2014	• CDSS (drop-down boxes)	26 Nurses	NA	Research utilisation	Baseline=35%<br>11 weeks=38%	3%	9% (3.3 to 14.7)
	• Control	24 Nurses	NA		Baseline=19%<br>11 weeks=13%	-6%	
Dumont et al,2012	• CDSS use	Nurses (OA=44)	141 adults	Nurses satisfaction, out of 10 (mean (SD))	4 months=8.4(1.4)	-	3.6 (2.4 to 4.8)
	• Paper protocol	Nurses	159 adults		4 months=4.8(2.4)		
	• CDSS use<br>• Paper protocol			perception of how often needed to deviate from the protocol, out of 10 (mean (SD))	4 months=2.7(2.2)<br>4 months=7.4(2.4)	-	-4.7 (-6.1 to -3.3)
Sassen et al,2014	• CDSS use	42 nurses and physios	Not reported	Behaviour, mean (SD)	Baseline=4.5 (1.02)<br>17 months=4.6 (0.85)	0.1 (0.93)	0.1 (-0.32 to 0.53)
	• Control	27 nurses and physios	Not reported		baseline=4.8 (0.69)<br>17 months=4.8 (0.82)	0 (0.75)	
	• CDSS use	42 nurses and physios		Intention, mean (SD)	Baseline=6.3 (1.0)<br>17 months=6.1 (1.1)	0.2 (1.05)	0.3 (-0.22 to 0.82)
	• Control	27 nurses and physios			Baseline=5.9 (1.15)<br>17 months=6.0 (0.91)	-0.1(1.05)	
	• CDSS use	42 nurses and physios		Attitude, mean (SD)	Baseline=6.3 (0.44)<br>17 months=6.3 (0.56)	0.0(0.05)	-0.1 (-0.13 to -0.07)
	• Control	27 nurses and physios			Baseline=6.2 (0.69)<br>17 months=6.3 (0.68)	0.1 (0.09)	
	• CDSS use	42 nurses and physios		Perceived behavioural control, mean (SD)	Baseline=4.7 (0.79)<br>17 months=5.0 (0.73)	0.3 (0.77)	-0.1 (-0.49 to 0.29)

For peer review only

• Control	27 nurses and physios		Baseline=4.9 (0.87)<br>17 months=5.3 (0.8)	0.4 (0.85)	
• CDSS use	42 nurses and physios	Subjective norms, mean (SD)	Baseline=5.5 (0.55)<br>17 months=5.6 (0.63)	0.1 (0.59)	0 (0.34 to 0.34)
• Control	27 nurses and physios		Baseline=5.6 (0.93)<br>17 months=5.7 (0.76)	0.1 (0.84)	
• CDSS use	42 nurses and physios	Moral norms, mean (SD)	Baseline=6.0 (0.63)<br>17 months=6.2 (0.7)	0.2 (0.67)	0.1 (-0.21 to 0.41)
• Control	27 nurses and physios		Baseline=6.2 (0.59)<br>17 months=6.3 (0.55)	0.1 (0.57)	
• CDSS use	42 nurses and physios	Barriers, mean (SD)	Baseline=3.1 (1.17)<br>17 months=3.2 (1.12)	0.1 (1.14)	0.3 (-0.23 to 0.83)
• Control	27 nurses and physios		Baseline=2.8 (1.01)<br>17 months=2.6 (0.96)	-0.2 (0.98)	

Note: **‡,** calculated from reported information unless stated otherwise; **†**, as reported by study authors.

For peer review only

Supplementary Table 6: Summary of adverse events results

Author & Year	Interventions	Health professionals	patient participants	Outcome measured	Outcome values reported	Risk difference (95% CI)‡
Cleveringa et al,2010	• CDSS use in diabetic patients	Nurses	1699 adults	cardiovascular events occurring	CDSS Vs usual care	-11% (-18 to -4)†
	• Usual care	Nurses	1692 adults			
Fitzmaurice et al,2000	• CDSS Nurse<br>• CDSS non-use	Nurses<br>Physicians	224 adults<br>143 adults	Serious adverse reaction events	1 year =3 (1.3%)<br>1 year =10 (7%)	-5.7% (-10.1 to -1.2)
	• CDSS Nurse<br>• CDSS non-use	Nurses<br>Physicians	224 adults<br>143 adults	Deaths	1 year =3 (1.3%)<br>1 year =9 (6.3%)	-5% (-9.2 to -0.7)
Snooks et al, 2014	CDSS<br>Control	17 Paramedics<br>19 Paramedics	436 adults<br>343 adults	Patients dying	1 year =19/436 (4.4%)<br>1 year=11/343 (3.2%)	1.2% (-1.5 to 3.8)

Note: **‡,** calculated from reported information unless stated otherwise; **†**, as reported by study authors.

For peer review only

Supplementary Table 7: Summary of economic costs and consequences results

Author & Year	Interventions	Health professionals	patient participants	Outcome measured	Outcome values reported	Difference (95% CI)[‡]
Cleveringa et al,2010	• CDSS use<br>• Usual care	Nurses<br>Nurses		Diabetes-related costs (excluding CHD)-€ discounted	CDSS Vs usual care	1,698.00 (187 to 3,209)[†]
	• CDSS use<br>• Usual care			Cardiovascular disease cost-€ discounted	CDSS Vs usual care	-587.00 (-880 to -294)[†]
	• CDSS use<br>• Usual care			Diabetic care protocol cost-€ discounted	CDSS Vs usual care	316.00 (315 to 318)[†]
	• CDSS use<br>• Usual care			Total cost-€ discounted	CDSS Vs usual care	1,415.00 (-130 to 2,961)[†]
	• CDSS use<br>• Usual care			Total costs per QALY gained (Euro)	CDSS Vs usual care	38,243.00[†]
Guerts et al, 2017	• CDSS use<br>• Usual care	Nurses<br>Nurses	113 children<br>109 children	Average emergency department visit costs (Euro)	156.4<br>156.4	0.00
	• CDSS use<br>• Usual care			Average diagnostics cost (Euro)	1.09<br>1.55	-0.46
	• CDSS use<br>• Usual care			Average treatment cost (Euro)	4.48<br>2.58	1.90
	• CDSS use<br>• Usual care			Average follow-up/hospitalization (Euro)	134.<br>107.4	26.60
	• CDSS use<br>• Usual care			Average costs of missed diagnoses/adverse events (Euro)	49.70<br>81.8	-32.10
	• CDSS use<br>• Usual care			Average cost of CDSS implementation (Euro)	61.95<br>0.0	61.95
	• CDSS use<br>• Usual care			Overall average cost	408<br>350	58.00
Lattimer et al,2000	• CDSS<br>• Usual care	Nurses<br>Physicians	Not applicable<br>Not applicable	Net savings [of CDSS use] in a year (£)	CDSS Vs usual care	13,185 (-77,509 to 123,824)[†]
	• CDSS<br>• Usual care			Cost saved from inpatient stay	CDSS Vs usual care	51,059[†]
Snooks et al, 2014	• CDSS<br>• Control	Paramedics<br>Paramedics		Implementing cost of CCDS in one month (in 100s £)	74	74
	• CDSS<br>• Control			Total cost of implementation in one month (in 100s £)	2,773<br>2,526	247 (-247 to 741)[†]
	• CDSS			Net resources saved		39[†]

For peer review only

• Control	by CDSS per patient year (£)		
• CDSS<br>• Control	Net cost resources saved by CCDS per patient year (£)		208-308†
• CDSS<br>• Control	Mean length of Job cycle time (minutes)	CDSS Vs control	8.9 min (2.3 to 15.3)†
• CDSS<br>• Control	Mean length of episode of care (minutes)	CDSS Vs control	-5.7 min (-38.5 to 27.2)†

Note: **‡,** calculated from reported information unless stated otherwise; **†**, as reported by study authors; PCS, physical component summary; MCS, mental component summary; SF, Short-Form

For peer review only

PRISMA 2009 Checklist

Section/topic	#	Checklist item	Reported on page #
**TITLE**			
Title	1	Identify the report as a systematic review, meta-analysis, or both.	1
**ABSTRACT**			
Structured summary	2	Provide a structured summary including, as applicable: background; objectives; data sources; study eligibility criteria, participants, and interventions; study appraisal and synthesis methods; results; limitations; conclusions and implications of key findings; systematic review registration number.	2
**INTRODUCTION**			
Rationale	3	Describe the rationale for the review in the context of what is already known.	3
Objectives	4	Provide an explicit statement of questions being addressed with reference to participants, interventions, comparisons, outcomes, and study design (PICOS).	3
**METHODS**			
Protocol and registration	5	Indicate if a review protocol exists, if and where it can be accessed (e.g., Web address), and, if available, provide registration information including registration number.	3
Eligibility criteria	6	Specify study characteristics (e.g., PICOS, length of follow-up) and report characteristics (e.g., years considered, language, publication status) used as criteria for eligibility, giving rationale.	3-4
Information sources	7	Describe all information sources (e.g., databases with dates of coverage, contact with study authors to identify additional studies) in the search and date last searched.	3-4
Search	8	Present full electronic search strategy for at least one database, including any limits used, such that it could be repeated.	3-4
Study selection	9	State the process for selecting studies (i.e., screening, eligibility, included in systematic review, and, if applicable, included in the meta-analysis).	4
Data collection process	10	Describe method of data extraction from reports (e.g., piloted forms, independently, in duplicate) and any processes for obtaining and confirming data from investigators.	
Data items	11	List and define all variables for which data were sought (e.g., PICOS, funding sources) and any assumptions and simplifications made.	4,17
Risk of bias in individual studies	12	Describe methods used for assessing risk of bias of individual studies (including specification of whether this was done at the study or outcome level), and how this information is to be used in any data synthesis.	4
Summary measures	13	State the principal summary measures (e.g., risk ratio, difference in means).	5
Synthesis of results	14	Describe the methods of handling data and combining results of studies, if done, including measures of consistency (e.g., I^2) for each meta-analysis.	5

For peer review only - http://bmjopen.bmj.com/site/about/guidelines.xhtml

PRISMA 2009 Checklist

Section/topic	#	Checklist item	Reported on page #
Risk of bias across studies	15	Specify any assessment of risk of bias that may affect the cumulative evidence (e.g., publication bias, selective reporting within studies).	NA
Additional analyses	16	Describe methods of additional analyses (e.g., sensitivity or subgroup analyses, meta-regression), if done, indicating which were pre-specified.	NA
**RESULTS**			
Study selection	17	Give numbers of studies screened, assessed for eligibility, and included in the review, with reasons for exclusions at each stage, ideally with a flow diagram.	5-6
Study characteristics	18	For each study, present characteristics for which data were extracted (e.g., study size, PICOS, follow-up period) and provide the citations.	8-10
Risk of bias within studies	19	Present data on risk of bias of each study and, if available, any outcome level assessment (see item 12).	NA
Results of individual studies	20	For all outcomes considered (benefits or harms), present, for each study: (a) simple summary data for each intervention group (b) effect estimates and confidence intervals, ideally with a forest plot.	11-14
Synthesis of results	21	Present results of each meta-analysis done, including confidence intervals and measures of consistency.	NA
Risk of bias across studies	22	Present results of any assessment of risk of bias across studies (see Item 15).	NA
Additional analysis	23	Give results of additional analyses, if done (e.g., sensitivity or subgroup analyses, meta-regression [see Item 16]).	NA
**DISCUSSION**			
Summary of evidence	24	Summarize the main findings including the strength of evidence for each main outcome; consider their relevance to key groups (e.g., healthcare providers, users, and policy makers).	14-17
Limitations	25	Discuss limitations at study and outcome level (e.g., risk of bias), and at review-level (e.g., incomplete retrieval of identified research, reporting bias).	17
Conclusions	26	Provide a general interpretation of the results in the context of other evidence, and implications for future research.	17
**FUNDING**			
Funding	27	Describe sources of funding for the systematic review and other support (e.g., supply of data); role of funders for the systematic review.	17

From: Moher D, Liberati A, Tetzlaff J, Altman DG, The PRISMA Group (2009). Preferred Reporting Items for Systematic Reviews and Meta-Analyses: The PRISMA Statement. PLoS Med 6(7): e1000097. doi:10.1371/journal.pmed1000097

For more information, visit: **www.prisma-statement.org**.

For peer review only - http://bmjopen.bmj.com/site/about/guidelines.xhtml

BMJ Open

Effects of computerised clinical decision support systems (CDSS) on nursing and allied health professional performance and patient outcomes: A systematic review

Journal:	*BMJ Open*
Manuscript ID	bmjopen-2021-053886.R1
Article Type:	Original research
Date Submitted by the Author:	26-Sep-2021
Complete List of Authors:	Mebrahtu, Teumzghi; University of Leeds, School of computing<br>Skyrme, Sarah; University of Leeds, School of Healthcare<br>Randell, Rebecca; University of Bradford, Faculty of Health Studies; Wolfson Centre for Applied Health Research<br>Keenan, Anne-Maree; University of Leeds, School of Healthcare<br>Bloor, Karen; University of York, Department of Health Sciences<br>Yang, Huiqin; University of Leeds, School of Healthcare<br>Andre, Deirdre; University of Leeds, Library Services<br>Ledward, Alison; University of Leeds, School of Healthcare<br>King, Henry; University of Leeds, School of Healthcare<br>Thompson, Carl; University of Leeds, School of Healthcare
**Primary Subject Heading**:	Health informatics
Secondary Subject Heading:	Health policy
Keywords:	Health informatics < BIOTECHNOLOGY & BIOINFORMATICS, Information technology < BIOTECHNOLOGY & BIOINFORMATICS, Information management < BIOTECHNOLOGY & BIOINFORMATICS

SCHOLARONE™
Manuscripts

I, the Submitting Author has the right to grant and does grant on behalf of all authors of the Work (as defined in the below author licence), an exclusive licence and/or a non-exclusive licence for contributions from authors who are: i) UK Crown employees; ii) where BMJ has agreed a CC-BY licence shall apply, and/or iii) in accordance with the terms applicable for US Federal Government officers or employees acting as part of their official duties; on a worldwide, perpetual, irrevocable, royalty-free basis to BMJ Publishing Group Ltd ("BMJ") its licensees and where the relevant Journal is co-owned by BMJ to the co-owners of the Journal, to publish the Work in this journal and any other BMJ products and to exploit all rights, as set out in our licence.

The Submitting Author accepts and understands that any supply made under these terms is made by BMJ to the Submitting Author unless you are acting as an employee on behalf of your employer or a postgraduate student of an affiliated institution which is paying any applicable article publishing charge ("APC") for Open Access articles. Where the Submitting Author wishes to make the Work available on an Open Access basis (and intends to pay the relevant APC), the terms of reuse of such Open Access shall be governed by a Creative Commons licence – details of these licences and which Creative Commons licence will apply to this Work are set out in our licence referred to above.

Other than as permitted in any relevant BMJ Author's Self Archiving Policies, I confirm this Work has not been accepted for publication elsewhere, is not being considered for publication elsewhere and does not duplicate material already published. I confirm all authors consent to publication of this Work and authorise the granting of this licence.

For peer review only

Effects of computerised clinical decision support systems (CDSS) on nursing and allied health professional performance and patient outcomes: A systematic review

Mebrahtu TF[1*], Skyrme S[2], Randell R[3], Keenan AM[2], Bloor K[4], Yang H[2], Andre D[5], Ledward A[2], King H[2], Thompson CA[2]

[1]School of Computing, Faculty of Engineering and Physical Sciences, University of Leeds, Leeds, UK

[2]School of Healthcare, University of Leeds, Leeds, UK

[3] Faculty of Health Studies, University of Bradford, Bradford, UK; Wolfson Centre for Applied Health Research, Bradford, UK

[4]Department of Health Sciences, University of York, York, UK

[5] Library Services, University of Leeds, Leeds, UK.

***corresponding author:** t.f.mebrahtu@leeds.ac.uk; School of Computing, Faculty of Engineering and Physical Sciences, University of Leeds, Leeds, LS2 9JT

Keywords: decision support; nursing; allied health professionals; CDSS; decision making

Word count:

Abstract=372

Main text=4247

For peer review only - http://bmjopen.bmj.com/site/about/guidelines.xhtml

ABSTRACT

Objective: Computerised clinical decision support systems (CDSS) are an increasingly important part of nurse and allied health professional (AHP) roles in delivering healthcare. The impact of these technologies on these health professionals' performance and patient outcomes has not been systematically reviewed. We aimed to conduct a systematic review to investigate this.

Materials and Methods: The following bibliographic databases and grey literature sources were searched by an experienced Information Professional for published and unpublished research from inception to February 2021 without language restrictions: MEDLINE(Ovid) , Embase Classic+Embase (Ovid), PsycINFO (Ovid), HMIC (Ovid), AMED (Allied and Complementary Medicine) (Ovid) , CINAHL (EBSCO), Cochrane Central Register of Controlled Trials (Wiley, Cochrane Database of Systematic Reviews (Wiley), Social Sciences Citation Index Expanded (Clarivate), ProQuest Dissertations & Theses Abstracts & Index, ProQuest ASSIA (Applied Social Science Index and Abstract), Clinical Trials.gov, World Health Organisation International Clinical Trials Registry (ICTRP), Health Services Research Projects in Progress (HSRProj), OpenClinical(www.OpenClinical.org), OpenGrey (www.opengrey.eu), Health.IT.gov, Agency for Healthcare Research and Quality (www.ahrq.gov). Any comparative research studies comparing CDSS with usual care were eligible for inclusion.

Results: A total of 36,106 non-duplicate records were identified. Of 35 included studies: 28 were randomised trials, three controlled-before-and-after studies, three interrupted-time-series and one non-randomised trial. There were ~1,318 health professionals and ~67,595 patient participants in the studies. Most studies focused on nurse decision makers (71%) or paramedics (5.7%). CDSS as a standalone Personal Computer (PC)/LAPTOP-technology was a feature of 88.7% of the studies; only 8.6% of the studies involved "smart" mobile/handheld-technology.

Discussion: CDSS impacted 38% of the outcome measures used positively. Care processes were better in 47% of the measures adopted; Examples included, nurses' adherence to hand disinfection guidance, insulin dosing, on-time blood sampling, and documenting care. Patient care outcomes in 40.7% of

For peer review only - http://bmjopen.bmj.com/site/about/guidelines.xhtml

indicators were better; examples included, lower numbers of falls and pressure ulcers, better glycaemic control, screening of malnutrition and obesity, and triaging appropriateness.

Conclusion: CDSS may have a positive impact on selected aspects of nurses' and AHPs' performance and care outcomes. However, comparative research is generally low quality, with a wide range of heterogeneous outcomes. After more than 13 years of synthesised research into CDSS in healthcare professions other than medicine, the need for better quality evaluative research remains as pressing.

Strengths and limitations of the review:

- The review is based on a comprehensive literature search
- This is the first systematic review of CDSS influence on nursing and AHP performance and outcomes
- Allied Health Professionals are under-represented, with a primary focus on paramedics and physiotherapists
- The number of studies, service users/patients, and health professionals involved was sizable, but outcomes were too heterogeneous to aggregate
- The overall quality of comparative research represented by the included studies was poor.

For peer review only

For peer review only - http://bmjopen.bmj.com/site/about/guidelines.xhtml

INTRODUCTION

Nurses and allied health professionals' (AHPs') judgements and decisions commit financial, human, and technical resources to care in health systems.[1] To support decision making and underpin new roles and ways of delivering services, such as nurse-led primary care,[1] computerised clinical decision support systems (CDSS) have been developed to tailor evidence-based advice provided to clinicians at the point of decision making.

CDSS can improve professional performance by making the basis for decisions explicit; widening available information, encouraging more consistent decisions and thus reducing unwarranted variation in processes and patient outcomes.[2 3] Negatively, CDSS could encourage a focus on unimportant problems, hinder care delivery and contribute to a widening of (digital) inequalities.[4-6]

Reviews focusing mainly on doctors suggest CDSS effects on performance and outcomes are inconsistent [7] but improved care processes[8 9] and reduced morbidity [8] and mortality [10] are possible. These reviews, however, often neglect the multi-disciplinary nature of healthcare delivery and the decisions involved.

Previously synthesised studies of nurses' use of CDSS suggest only limited impact on performance and health outcomes.[11] Digital technology and research evidence have both developed significantly since this review was undertaken. In this review we aim to examine the impact of CDSS on nurses' and allied health professionals' (AHPs) performance and patient outcomes.

REVIEW METHODS

Following best practice principles [12 13] we undertook a systematic review of research into CDSS targeting nurse and AHP decision makers. The protocol was registered with PROSPERO [14] [number: CRD42019147773].

Literature searching

Initial searches were conducted in November 2019 and updated on 12 February 2021. Searches were not restricted by language. See Supplementary Table 1 for search terms.

For peer review only - http://bmjopen.bmj.com/site/about/guidelines.xhtml

We searched: MEDLINE(Ovid) , Embase Classic+Embase (Ovid), PsycINFO (Ovid), Health Management Information Consortium (HMIC) (Ovid), AMED (Allied and Complementary Medicine) (Ovid) , CINAHL , Cochrane Central Register of Controlled Trials (Wiley, Cochrane Database of Systematic Reviews (Wiley), Social Sciences Citation Index Expanded (Clarivate), ProQuest Dissertations & Theses Abstracts & Index, ProQuest ASSIA (Applied Social Science Index and Abstract), Clinical Trials.gov, World Health Organisation International Clinical Trials Registry (ICTRP), Health Services Research Projects in Progress (HSRProj), OpenClinical(www.OpenClinical.org), OpenGrey (www.opengrey.eu), Health.IT.gov, Agency for Healthcare Research and Quality (www.ahrq.gov).

Study inclusion and exclusion

All titles and abstracts were imported into a reference management database (EndNote) and duplicates removed. Covidence review production toolkit (www.covidence.org) was used to manage screening, data extraction and organising of the review and ensure efficient production. After removing duplicate titles and abstracts, seven reviewers (AK, CT, HY, HK RR, SS and TM) independently screened all titles and abstracts. TM first-screened titles and abstracts for all studies, the other six authors then second-screened 16.7% of the studies each. Records with decision disagreements were revisited by two authors (TM and CT) and resolved by consensus, a third reviewer (RR) was available for further disagreements although none occurred. Two reviewers (CT and TM) independently assessed study relevance using Cochrane Collaboration's Effective Practice and Organisation of Care (EPOC) criteria;[15] and, conducted full-text screening. Any disagreements were resolved by consensus.

Comparative studies (randomised controlled trials (RCTs), non-randomised trials, controlled before-after (CBA) studies, interrupted time series (ITS) studies and repeated measures studies) comparing CDSS against usual care (i.e., clinical decision making unsupported by CDSS) were eligible for inclusion.

Participants

For peer review only - http://bmjopen.bmj.com/site/about/guidelines.xhtml

Studies that evaluate the effects of CDSS used by **nurses [including midwives] and AHPs** and report professional performance and patient outcomes were eligible for inclusion.

Interventions

The eligible intervention in this review was the use of **any form of CDSS to aid clinical decision making**.

Comparator

The comparator was **usual care**; defined as **clinical practice where clinical decision making is unsupported by CDSS**.

Outcomes

Our primary outcome was **adherence of nurses and AHPs to evidence-based recommendations**. Secondary outcomes were *diagnostic accuracy, time to reach judgment, adverse events, health professional satisfaction, and system and/or implementation costs and benefits*.

Data extraction

Data on study characteristics and outcomes were independently extracted by two reviewers (CT and TM) using the EPOC standard data collection form.[16]

Quality assessment

Study quality and risk of bias was assessed independently by CT and TM using Cochrane Handbook for Systematic Reviews of Interventions[17] and EPOC guidelines.[18]

Each potential source of bias was judged as high, low, or unclear, and an overall 'risk of bias' classification (high, moderate, or low) assigned to each included study.[17] Studies with low risk of bias in all domains, or where bias was unlikely to fundamentally alter results, were treated as low risk. Studies with bias risk in at least one domain, or where bias might alter conclusions, were treated as unclear. Studies with a high risk of bias in at least one domain, or with a serious bias likely to reduce the certainty of conclusions, were considered high risk.

For peer review only - http://bmjopen.bmj.com/site/about/guidelines.xhtml
For peer review only

Data synthesis

Findings were synthesised narratively, regardless of statistical analysis in the primary study. Studies were grouped by i) similarity in focus or CDSS-type (knowledge based or machine learning), ii) health professionals targeted, iii) patient group, iv) outcomes reported, and, v) study design.

If not reported, we calculated absolute risks from the primary research. Risk differences and 95% confidence intervals were then calculated from these. Because the CDSS, participants, and underlying research questions were so heterogeneous no meta-analysis was undertaken.[19]

For peer review only

RESULTS

Evidence Quantity

From 36,106 non-duplicate records identified, 35,858 records were excluded after title and abstract screening. Seven records were identified through forward citation searching. Full text screening was undertaken on 255 records which led to 220 more records being excluded. Thirty-five studies were included in the review.[20-51] **Figure 1** illustrates study selection.

Study Descriptions

The 35 included studies comprised 28 RCTs (80%), three CBA studies (8.6%), three ITS (8.6%) and one non-randomised trial (2.8%). Thirty-two studies (91.4%) were peer-reviewed journal articles and three (8.6%) were PhD theses. The public sector funded 74.3% of studies; industry, 5.7%; 17.1% failed to declare funding and 2.9% were unfunded. Most studies were published after 2010 (n=29, 82.9%) with just two studies during 1997-1999 and 14 (40.0%) in 2000-2010. Sixteen studies (45.6%) were published after the last significant systematic review on CDSS for nurses' performance and health outcomes.[11] Circa 1,318 health professionals and 67,595 patients were study participants, mainly in hospital-based studies (57.1%). Primary care accounted for 17.1% and nursing homes 11.4% of studies. Western health systems provided the dominant context: US (28.6%); UK (20.0%), Netherlands (17.2%), Czech Republic and Norway (5.7%) each. With single study representation (2.8%) from Belgium, Brazil,

For peer review only - http://bmjopen.bmj.com/site/about/guidelines.xhtml

China, Ghana, Norway, Sweden, Turkey and one multicentre (Austria, Czech Republic, and UK) report. See **Table 1**.

Only one study (of 35) reported explicit theory to guide implementation of the CDSS. Almost a third (28%) published their study protocol – none of which discussed theory-influenced implementation.

Nurses made up the target for the CDSS *and* control groups in 25 (71.4%) studies; paramedics in two (5.7%) studies. Five studies (14.3%) compared nurses in the intervention (CDSS) group with physicians in the control. Two studies (5.7%) recruited a combination of nurses and physiotherapists for CDSS and control groups. Thirty-one studies (88.7%) used a standalone (physically, even when integrated in an electronic health record) computer-based CDSS; three (8.6%) used handheld/mobile-based technologies, and just one study (0.2%) used a web-based CDSS. CDSS were mostly designed with a single function in mind (e.g., disease diagnosis), but some addressed multiple parts of clinical pathways (e.g., disease diagnosis *and* disease management).

Quality of identified evidence

Except for three RCTs scored as 'Unclear', all studies were at 'high' overall risk of bias. On average, RCTs scored 'Low' risk of bias in five of nine domains; CBA studies were lower, with four domains; non-randomised studies scored 'low' for a single domain. The three ITS studies were 'Low' risk of bias in six (of seven) domains. Evidence quality did not change over time (see Supplementary Table 2).

For peer review only

For peer review only - http://bmjopen.bmj.com/site/about/guidelines.xhtml

Table 1 Baseline characteristics of included studies

Author and year	Country	Design	Setting	Study duration	Healthcare professionals (HP)	Outcomes
Beeckman et al, 2013	Belgium	RCT	Nursing homes	5 months	Nurses and physios	Risk of pressure ulcers; HP knowledge and attitude
Bennet et al, 2016	UK	ITS	Emergency department, district general hospital	1 year	Nurses	Triage prioritization; pain assessment and management; management of neutropenic sepsis
Blaha et al, 2009	Czech Republic	RCT	ICU post elective cardiac surgery university hospital	48 hours	Nurses	Intensive care glycaemic control/diabetes
Byrne, 2005	USA	CBA	Nursing homes	33 months	Nurses	Falls and pressure ulcer reduction (assessment and prevention)
Canbolat et al,2019	Turkey	Non-RT	ICU university general hospital	22 months	Nurses [and physicians]	ICU glycaemic control
Cavalcanti et al, 2009	Brazil	RCT	ICU general hospital	19 months	Nurses	ICU glycaemic control
Cleveringa et al, 2008	Netherlands	RCT	Primary care practices	1 year	Nurses [and physicians)	Management and prevention of diabetes (and CV risk factors)
Cleveringa et al, 2010	Netherlands	RCT	Primary care practices	1 year	Nurses	Management and prevention of diabetes (and CV risk factors)
Cortez, 2014	USA	RCT	Academic medical centre oncology clinics	11 weeks	Nurses	Management of cancer symptoms
Dalaba 2015	Ghana	CBA	Primary care health centres	2 years	Nurses	Maternal care
Dowding et al,2012	USA	ITS	General hospitals	6 years	Nurses	Risk assessment, falls and pressure ulcer prevention
Duclos et al, 2015	France	RCT	Paediatric wards in a university hospital	2 years	Dieticians	Nutritional care in malnourished children
Dumont et al,2012	USA	RCT	ICU wards in a regional referral hospital	4 months	Nurses	Glycaemic control
Dykes et al, 2009	USA	RCT	Urban hospitals	6 months	Nurses	Fall prevention
Dykes et al, 2020	USA	ITS	Academic medical centres	42 months	Nurses	Fall prevention
Fitzmaurice et al,2000	UK	RCT	primary care/general practice	1 year	Nurses	oral anticoagulation care
Forberg et al, 2016	Sweden	RCT	paediatric university hospital	3 months	Nurses	management of peripheral venous catheters in paediatrics
Fossum et al, 2011	Norway	CBA	Nursing homes	2 years	Nurses	Preventative behaviours and management of nutrition

For peer review only - http://bmjopen.bmj.com/site/about/guidelines.xhtml

Geurts et al, 2017	Netherlands	RCT	University paediatric hospital	2 years	Nurses	Management of (re)hydration in children
Hovorka et al, 2007	Czech Republic	RCT	Cardiac Surgery, University Hospital	48 hours	Nurses	Glycaemic control
Kroth et al, 2006	USA	RCT	University Hospital	9 months	Nurses	Body temperature assessment
Lattimer et al, 1998	UK	RCT	Primary care practices	1 year	Nurses & physicians	Emergency call assessment
Lattimer et al, 2000	UK	RCT	Primary care practices	1 year	Nurses & physicians	Cost analysis of emergency call assessments
Lee et al, 2009	USA	RCT	School of Nursing (University)	8 months	Nurses	Obesity management
Lv et al, 2019	China	RCT	Community healthcare centres	1 year	Nurses	Chronic asthma management
Mann et al, 2011	USA	RCT	Surgical Military hospital ICU	6 days	Nurses	Glycaemic control in burn intensive care patients
McDonald et al, 2017	USA	RCT	Nursing care homes	2 months	Nurses	Management of chronic medical condition
Paulson et al, 2020	Norway	RCT	University hospital	10 months	Nurses	Management of malnutrition
Plank et al, 2006	Mixed (Austria, Czech Republic, UK)	RCT	University hospitals	48 hours	Nurses	Glycaemic control
Rood et al, 2005	Netherlands	RCT	Surgical ICU in a teaching hospital	10 weeks	Nurses	Glycaemic control
Roukema et al, 2008	Netherlands	RCT	Children's Hospital	27 months	Nurses	Management of children with fever without apparent source
Sassen et al, 2014	Netherlands	RCT	University research centre	17 months	Nurses and physios	professionals' behaviour
Snooks et al, 2014	UK	RCT	Emergency ambulance services	1 year	Paramedics	Assessment and management of falls
Vadher et al, 1997	UK	RCT	Cardiovascular medicine, general hospital		A nurse and Trainee doctors	oral anticoagulant control
Wells, 2013	UK	RCT	Emergency ambulance services	1 year	Paramedics	Emergency fall assessment and management

Note: CBA, controlled before and after; CDSS, computerised decision support; HPs, health professionals; ITS, interrupted time-series; RCT, randomised controlled trials

For peer review only - http://bmjopen.bmj.com/site/about/guidelines.xhtml

Effects of intervention

Most studies reported more than two outcomes from a total of 124 individual outcomes reported (115 distinct types of measured outcomes). There were five distinct outcome groups:

- Care processes: aspects of patient data collection and management, and the process of patient management
- Care outcomes: patient health outcomes (e.g. fall and pressure ulcer prevention rate)
- Health professionals' knowledge, beliefs, and behaviours: outcomes that relate to the health professionals themselves (e.g. changed attitude and perception due to CDSS use)
- Adverse events: safety issues that could arise due to the use of CDSS (e.g. morbidity)
- Economic costs and consequences: outcomes that relate to direct costs , savings, or cost-effectiveness of CDSS

Care process

CDSS was better than usual care for 16 of 34 (47.0%) care process outcomes. Care delivery was worse (n=5, 14.7%) or no different for 13 (38.2%) processes. See Supplementary Table 3.

Adherence to guidelines

The four RCTs reporting nurses' adherence to guidelines examined 10 outcomes.[32 34 45 49] Only one trial reported baseline and follow-up data for both arms,[34] CDSS users had better adherence to hand disinfection guidelines (risk difference=6.7%; 95% CI: 4.9 to 8.5%); but were less likely to follow guidelines on disposable glove use (risk difference= -1.4%; 95% CI: -2.2 to -0.5%) and daily inspections of Peripheral Venous Catheters (risk difference=-5.2%; 95% CI: -7.2 to -3.3%).

Two trials [32 45] showed nurses using CDSS had better compliance with guidelines on insulin dosing (risk difference=22%; 95% CI: 19 to 25%) and on-time blood sampling (risk difference=4.7%; 95% CI: 2.0 to 7.4%). They deviated less from protocols (mean score difference out of 10 =-2.6; 95% CI: -4.5 to -0.71) and concurred more with recommended insulin doses (than trainee doctors).[49]

For peer review only - http://bmjopen.bmj.com/site/about/guidelines.xhtml

Patient assessment, diagnosis, and treatment practices

Five RCTs [31 36 38 46 50] and one ITS [21] reported 18 indicators of patient assessment and treatment quality. Pain assessment quality (pain score use and appropriateness of choices) of emergency department patients improved by 62.7% (95% CI: 59.6 to 65.8%) and investigation of in-patient paediatric malnutrition aetiology was 21.2% higher (95% CI: 15.9 to 26.5%) with CDSS. However, optimal IV antibiotics administration for sepsis was lower reduced by 5.9% (95% CI: -8.3 to -3.5). Laboratory tests (electrolytes level acid-base balance test) and nutrition supplements (oral Rehydration Solution and intravenous rehydration) were no more likely to be ordered for paediatric inpatients by CDSS-enabled nurses.

There were marginally fewer wrongly recorded temperatures in hospital inpatients amongst CDSS-enabled nurses (risk difference= -0.8%, 95% CI: -0.9 to -0.6). Vital signs recording in patients attended by paramedics were also not significantly different.

Documenting care

One ITS and a randomised trial reported five documentation-focused indicators.[30 52] Falls (risk ratio=1.4, 95% CI: 0.03 to 73.7) and hospital acquired pressure ulcer risk assessments (risk ratio=9.1, 95% CI: 1.95 to 42.5) were higher with CDSS. As was nutritional care planning, food and fluid intake recording and treatment by nurses.[52]

Referrals

Paramedics using CDSS were more likely to refer patients to a community falls than send them to the emergency department (risk difference=4.7%, 95% CI: 1.1. to 8.3).[48]

Patient care outcomes

CDSS improved patient care outcomes in 22 of 54 (40.7%) indicators and worsened them for 1 outcome indicator (2.0%). See Supplementary Table 4.

Blood glucose control

For peer review only - http://bmjopen.bmj.com/site/about/guidelines.xhtml

Six RCTs [22 25 26 37 42 44] and one non-randomised trial [24] reported 19 indicators of glycaemic control, but only two reported baseline *and* follow-up values [22 26]. Blood glucose levels were better managed by ICU nurses using CDSS (mean=-2.2, SD=1.12) compared to paper-based *Mathias* (mean=-1.2, SD=0.66) and *Bath* (mean=-1.5, SD=0.78) protocols.[22] Glycated haemoglobin (A1C) <7%, systolic blood pressure <140 and total cholesterol<4.5mmol/l were higher by 4.6% (95% CI: 2.7 to 6.5), 10.2% (95% CI: 7.9 to 12.5) and 3.7% (95% CI: 1.2 to 6.2) respectively in patients receiving care from CDSS-enabled nurses compared.

Trials reporting only follow-up data suggest better blood glucose control by CDSS-using nurses across a range of indicators: proportion in target range (risk difference=32.9%; 95% CI: 20.0 to 46.0), occasions within the target glycaemic range (80-110 mg/dl) (risk difference= 33.0%, 95% CI: 20.5 to 45.4), occasions over the target glycaemic range (>110 mg/dl) (risk difference= -31.0%, 95% CI: -43.7 to -18.2), and improvement of glycaemic control for 48 hours (risk difference=40.0%, 95% CI: 27.4 to 52.6)

Blood coagulation management

One RCT reported three indicators of blood coagulation management in primary care.[33] Nurses using CDSS had significantly more tests in range (risk difference=4.0%, 95% CI: 0.4 to 7.6) than doctors *without* CDSS. However, the improvement from baseline was lower amongst nurses (risk difference=-1.9% (95% CI: -3.1 to -0.7), 'International Normalised Ratio (INR) Results within Range Point Prevalence' were not significantly different between the two groups and again, nurses using CDSS improved less than physicians without CDSS (risk difference=-2.6%, 95% CI: -5.3 to -0.1). There was no significant difference between groups in 'Time Spent within INR Target Range' (risk difference=7.0%, 95% CI: -0.7 to 14.7).

Antenatal and peripartum care

For peer review only - http://bmjopen.bmj.com/site/about/guidelines.xhtml

The CBA study examining antennal and peripartum care in community settings[29] suggested CDSS-using midwives reduced delivery complications (per 1000 attendances) compared to usual care (risk difference=2.4%, 95% CI: 1.1 to 3.7).

Managing patients with chronic co-morbid diseases

Two RCTs examined three indicators of successfully managing patients with complex chronic multi-morbid health conditions in care homes,[43] and with asthma[53] showed no significant differences between CDSS users and non-users for emergency room usage, hospitalisation and complexity of medication regimens.

Obesity screening

The RCT examining outpatient obesity screening by trainee nurses found CDSS-users had more 'encounters with obesity-related diagnosis' (risk difference=10.3%, 95% CI: 8.0 to 12.5) and fewer 'encounters with missed obesity-related missed diagnosis' (risk difference=41.0%, 95% CI: 48.8 to 35.0) than trainee nurses without CDSS.[41]

Fall and pressure ulcer prevention and management

Two RCTs, [20 51] two CBA studies [23 35] and two ITS [30 54] focused on fall or pressure ulcer prevention and management. In a single trial,[20] pressure ulcer prevalence decreased more during the CDSS-enabled follow-up period (risk difference=-6.3%, 95% CI: -10.2 to -2.4), a result which was reversed in one of the CBA studies (risk difference=4.2%, 95% CI: 0.2 to 8.2).[35] The other CBA studies revealed no significant differences between CDSS using and non-using nurses trying to prevent falls and pressure ulcers.[23] In the ITS study, fall rate (risk ratio=0.91, 95% CI: 0.75 to 1.12) and hospital acquired pressure ulcer occurrence (risk ratio=0.47, 95% CI: 0.25 to 0.85) were significantly lower with CDSS.[30]

Triage

Three RCTs [39 40 48] and one ITS study [21] evaluated CDSS impact on triage judgements. Health professionals using CDSS made fewer calls to General Practitioners (GP) for telephone advice (risk difference= -34.2%, 95% CI: -36.0 to -33.0), had fewer patients visited at home by duty GPs (risk

For peer review only - http://bmjopen.bmj.com/site/about/guidelines.xhtml

difference=-5.5%, 95% CI: -6.9 to -4.2), and fewer hospital admissions within 3 days (risk difference=-0.98%, 95% CI: -1.8 to -0.2) of the judgement. There were no differences in, 'patients left at scene without conveyance to emergency department' (risk difference= 5.2%, 95% CI: -1.7 to 12.1). The ITS study reported the proportion of *correct (sic)* triage prioritisation judgements was higher amongst CDSS-users (risk difference=24.7%; 95% CI: 18.8 to 30.6).

Quality of life and patients' satisfaction

Two RCTs examined CDSS impact on quality of life and patient satisfaction.[27 48] Patients in CDSS-using groups gained more life years (average difference in years=0.14, 95% CI: -0.12 to 0.40), more healthy years (average difference in years = 0.04, 95% CI: -0.07 to 0.14) but reported lower quality of life and satisfaction. None of these differences were statistically significant.

Health professionals' knowledge, beliefs, and behaviour

CDSS effects on knowledge, beliefs, and behaviours of health professionals [20 28 32 47] was the focus of four RCTs using twelve indicators. CDSS increased 'Positive knowledge change' (risk difference=6.5%; 95% CI:0.8 to 13.2), 'positive attitude change' (risk difference=12.7%, 95% CI: 5.9 to 19.5), 'research utilisation' (risk difference=9%; 95% CI: 3.3 to 14.7), nurses' satisfaction (difference in satisfaction out of 10=3.6, 95% CI: 2.4 to 4.8), and perceived deviations from protocols (mean difference out of 10=-4.7, 95% CI: -6.1 to -3.3). Conversely, there was no significant impact on behaviours, intentions, perceived behavioural control, subjective and moral norms, barriers, and research utilisation of CDSS-using nurses and physiotherapists (Supplementary Table 5).

Adverse events

CDSS are not risk free, and three RCTs [27 33 48] used four indicators to examine adverse events. Cardiovascular events in patients with diabetes (risk difference=-11.0%, 95% CI: -18.0 to -4.0) and deaths in primary care patients (risk difference=-5.7%, 95% CI: -10.1 to -1.7) were lower in CDSS-using groups of professionals. Serious adverse reactions in primary care patients and deaths in patients recently fallen and attended by paramedics were no less likely (Supplementary Table 6).

For peer review only - http://bmjopen.bmj.com/site/about/guidelines.xhtml

Economic costs and consequences

Four RCTs [27 36 40 48] used 20 indicators to report economic costs and consequences of CDSS. Costs of managing cardiovascular disease were lower in CDSS users (cost difference=-€587.00, 95% CI: -880.00 to -294.00). Diabetes care cost more (cost difference=€326.00, 95% CI: 315.00 to 318.00); took longer per care task (‘mean length of job cycle time’ difference in minutes=8.9; 95% CI: 2.3 to 15.3) to generate an additional quality adjusted life-year (QALY) costing €38,243.00 (Supplementary Table 7).

DISCUSSION

Summary of main results

Our systematic review suggests CDSS may improve some aspects of nurses’ and AHPs’ performance and care outcomes. Thirty eight percent (38%) of indicators were better. Of 35 included studies, 26 (74.3%) reported CDSS-influenced care as better than care without CDSS on at least one outcome. In contrast, 8 studies (22.8%) showed no significant difference between CDSS and usual care, with 7 studies suggesting CDSS were less effective than usual care for at least one outcome.

Care processes

Processes of care were better if CDSS was in use in almost half the studies, 16 of 34 (47%); a headline that masks a very wide range of absolute improvement: from 0.7% to 62.7%. Hand disinfection protocol adherence, insulin dosing, blood sampling at the right time, and documented care were all better in CDSS users. This should be contrasted with the five (16.1%) outcomes where CDSS provided no advantages over usual care. Both sets of findings are mitigated further by the considerable uncertainty in trying to estimate a holistic picture: the effects in 13 care process indicators (41.9%) were not estimable; either because studies lacked power (lower than minimum acceptable of 80%) to detect a difference in the comparison groups, or appropriate confidence intervals were not reported or could not be calculated from information published.

Patient care outcomes

CDSS was associated with significantly better patient care outcomes across a broad range of 22 of 54 (40.7%) indicators (absolute difference between 4.6% and 42.9%). Just one indicator (1.8%) suggested

For peer review only - http://bmjopen.bmj.com/site/about/guidelines.xhtml

For peer review only

no significant difference. Nurses using CDSS had better blood glucose control in emergency care patients (in five out of seven studies involved) and nurses and physiotherapists using CDSS were associated with better fall risk and pressure ulcer management. Triage was improved in nurses using CDSS in emergency call centres and paramedics faced with “emergency falls” in older patients.

Health professionals’ knowledge, beliefs, and behaviour

Improved knowledge, beliefs, and behaviour occurred in three of 12 indicators (25%). Nurse and physiotherapist CDSS-users had more knowledge and better attitudes compared to non-users. Compared with usual care, nurses utilised more research, were more satisfied at work, and perceived a greater need to follow protocols if they used CDSS.

Adverse events

CDSS generated fewer adverse events across two of four indicators (50%). CDSS-using nurses had fewer cardiovascular events and reported deaths in their primary care patients compare to similar patients seen by doctors not using CDSS.

Economic costs and consequences

CDSS did not significantly increase costs, or save money. Costs per quality adjusted life-year (QALY) was €38,243.00 in one study – higher than the widely-accepted willingness-to-pay threshold of €20,000 per QALY[27] and the United Kingdom *de facto* threshold of £30,000 per QALY to be considered cost-effective by the National Institute for Health and Care Excellence.[55]

Comparison with other studies or reviews

Only one previous review has examined the effects of CDSS on nursing performance and patient outcomes.[11] Twenty new primary studies have been published since this review; but inconsistent outcomes and weaknesses in study designs and methods remain. Given the importance of implementation in effectiveness, it was noteworthy that most studies lacked a theoretical foundation for the implementation of CDSS. Similarly, many studies did not report using guidelines for designing, conducting/evaluating, and reporting CDSS-use. Of 35 included studies, just one used an explicit

For peer review only - http://bmjopen.bmj.com/site/about/guidelines.xhtml

implementation model/theory at design stage.[20] None of the studies discussed their findings with reference to implementation science/theory.

In their review of 100 trials – principally with doctors - Garg et al.[7] reported improved performance in 64% and better patient outcomes in 13% of studies. Our results suggest greater improvement may be possible for nursing work in particular (47% of process indicators and 41% of outcomes). Garg et al.[7] transformed improvement into a binary (yes/no) indicator and did not quantify the outcome improvements – making the clinical significance of improvements hard to ascertain.

Bright et al. [8] reviewed RCTs of CDSS with a range of health professional decision makers (doctors, nurses and AHPs). They reported improvements in processes of care (OR=1.55, 95% CI: 1.38 to 1.74) and morbidity (RR=0.88, 95% CI: 0.80 to 0.96), but no impact on mortality (OR=0.79, 95% CI: 0.54 to 1.15) or safety/adverse events (RR= 1.01, 95% CI: 0.90 to 1.14). However, outcomes measured were too heterogeneous for meta-analysis. The criteria for comparison groups was relaxed; the "intervention" sometimes included paper-based decision support and alternative CDSS systems were used as a comparator in some studies. Our review required there to be an indication for the use of CDSS and a comparator that ruled out CDSS-use as part of "usual care". Whilst we found improvements are *possible* from CDSS, comparison with Bright et al's findings would be unreliable.

Moja and colleagues' review of 18 RCTs [10] (including nurses and AHPs alongside doctors) found no significant difference in CDSS-attributable mortality (RR=0.96, 95% CI: 0.85 to 1.08) but lower morbidity (RR=0.82, 95% CI: 0.68 to 0.99). Whilst mortality and morbidity findings are similar to ours, their use of CDSS in the primary study comparator groups, again makes comparisons unreliable.

A recent review of 115 trials of CDSS, with a mix of health professionals, reported process improvements of the order of 5.8% (95% CI: 4.0% to 7.6%) with CDSS.[9] As with Bright et al. the 'comparator' criteria were unclear and outcome measures too heterogeneous for meta-analysis. Studies with more than two comparators were treated as different trials, meaning double counting

For peer review only - http://bmjopen.bmj.com/site/about/guidelines.xhtml

and multiple comparisons (p-hacking) could not be ruled out, confounding comparisons with our findings.

Strengths and limitations

Our review, whilst based on a comprehensive literature search, is a function of that literature. Consequently, we have highlighted primarily the impact of CDSS on nurses rather than AHPs. With the exception of paramedics and physiotherapists, other AHPs are poorly represented.

Evidence quality was poor and has not improved significantly since 2009. Whilst the number of studies (35), service users/patients (~67,000) and health professionals (~1,318) involved was sizable, outcomes were too heterogeneous for aggregation. Inconsistencies in the effects of CDSS on target health professionals' performance and patient outcomes remain unresolved. Moreover, although we have used a comprehensive list of databases in our search, the possibility of missing studies due to search terms cannot be ruled-out.

Conclusions

CDSS can benefit nurse and (some) AHP delivered performance and patient outcomes. CDSS can improve adherence to guidelines and enhance patient care. Triaging of emergency patients, glycaemic control, and screening of malnutrition and obesity all represent appropriate targets for CDSS. These conclusions require cautious interpretation: they are based on mainly low-quality studies, with heterogeneous outcomes and indicators.

To improve the quality of studies and consistency of outcomes, future research should satisfy two key requirements. First, system designers and evaluators should consider appropriate implementation theory/models (examples include Normalisation Process Theory [56]and the NASSS framework [57]) given the planned technology and associated work to encourage sustained adoption. Second, study reporting is varied, poor quality and lacking essential detail for implementation; guidelines for conducting and reporting CDSS should be a feature of the publication of findings. This would make

For peer review only - http://bmjopen.bmj.com/site/about/guidelines.xhtml

synthesis easier and more informative. Guidelines for CDSS reporting in general already exist, it is difficult to conceive why they cannot be applied to nursing and AHP-focused CDSS.[58 59]

FUNDING: The review is funded by the National Institute for Health Research Health Services Delivery and Research programme [award number: NIHR127926]. The views expressed in this publication are those of the authors and not necessarily those of the NHS, the NIHR or the Department of Health.

PATIENT AND PUBLIC INVOLVEMENT: The initial impetus for the bid came from discussions with Governors and service user representatives from Leeds Partnership NHS Foundation Trust. CT was a Non-Executive Director involved with the introduction of technology to help assessment by community mental health nurses. Because end-users of the review will be professionals and commissioners as well as patients and the public, we have set up an advisory group composed of people with specialist knowledge of digital health in a UK NHS context. One of our co-authors, Alison Ledward, is a public member of the UK NIHR HS&DR Researcher-Led Panel and has helped to shape our protocol. She has helped to ensure we maintain our focus on the effects of CDSS on patient outcomes and experiences and determining whether CDSS help nurses and AHPs make better decisions for patients.

AUTHOR CONTRIBUTIONS: AK, AL, CT, DA, HY, KB and RR contributed to conception of the review. DA conducted online database searches. AK, CT, HK, HY, RR, SS and TM contributed to titles and abstracts screening. CT and TM contributed to full text screening, quality assessment and data extraction. TM analysed and summarised data as well as produced the first draft of the manuscript. All authors have been involved in revising the work for important intellectual content and have approved the final version for publication.

COMPETING INTERESTS: None declared

For peer review only - http://bmjopen.bmj.com/site/about/guidelines.xhtml

PATIENTS CONSENT FOR PUBLICATION: Not required

ETHICS APPROVAL: Not required

DATA AVAILABILITY STATEMENT: Data used in this review are all included in the main document and supplementary materials published here with.

For peer review only

For peer review only - http://bmjopen.bmj.com/site/about/guidelines.xhtml

REFERENCES

1. Murdoch J, Barnes R, Pooler J, et al. The impact of using computer decision-support software in primary care nurse-led telephone triage: interactional dilemmas and conversational consequences. *Social Science & Medicine* 2015;126:36-47.
2. Anderson JA, Willson P. Clinical Decision Support Systems in Nursing: Synthesis of the Science for Evidence-Based Practice. 2008;26(3):151-58. doi: 10.1097/01.NCN.0000304783.72811.8e
3. Cresswell K, Majeed A, Bates DW, et al. Computerised decision support systems for healthcare professionals: an interpretative review. *Informatics in primary care* 2012;20(2):115-28. [published Online First: 2012/01/01]
4. Bonney W. Impacts and risks of adopting clinical decision support systems. *Efficient Decision Support Systems: Practice and Challenges in Biomedical Related Domain* 2011:21-30.
5. Langhorne P, Ramachandra S. Organised inpatient (stroke unit) care for stroke: network meta-analysis. *Cochrane Database of Systematic Reviews* 2020(4) doi: 10.1002/14651858.CD000197.pub4
6. Sintchenko V, Coiera E. Decision complexity affects the extent and type of decision support use. *AMIA Annual Symposium proceedings AMIA Symposium* 2006;2006:724-28.
7. Garg AX, Adhikari NKJ, McDonald H, et al. Effects of Computerized Clinical Decision Support Systems on Practitioner Performance and Patient OutcomesA Systematic Review. *JAMA* 2005;293(10):1223-38. doi: 10.1001/jama.293.10.1223 %J JAMA
8. Bright TJ, Wong A, Dhurjati R, et al. Effect of Clinical Decision-Support Systems: A Systematic Review. *Annals of Internal Medicine* 2012;157(1):29-43. doi: 10.7326/0003-4819-157-1-201207030-00450 %J Annals of Internal Medicine
9. Kwan JL, Lo L, Ferguson J, et al. Computerised clinical decision support systems and absolute improvements in care: meta-analysis of controlled clinical trials. *bmj* 2020;370
10. Moja L, Kwag KH, Lytras T, et al. Effectiveness of computerized decision support systems linked to electronic health records: a systematic review and meta-analysis. *Am J Public Health* 2014;104(12):e12-22. doi: 10.2105/AJPH.2014.302164 [published Online First: 2014/10/17]
11. Randell R, Mitchell N, Dowding D, et al. Effects of computerized decision support systems on nursing performance and patient outcomes: a systematic review. *Journal of Health Services Research & Policy* 2007;12(4):242-51. doi: 10.1258/135581907782101543
12. Centre for Reviews and Dissemination. Systematic Reviews: CRD's Guidance for Undertaking Reviews in Health Care. . York: Centre for Reviews and Dissemination: University of York 2009.
13. Liberati A, Altman DG, Tetzlaff J, et al. The PRISMA statement for reporting systematic reviews and meta-analyses of studies that evaluate health care interventions: explanation and elaboration. *Annals of internal medicine* 2009;151(4):W-65-W-94.
14. Thompson C, Randell R, Keenan AM, et al. Effects of computerised clinical decision support systems (CDSS) on nursing and allied health professional performance and patient outcomes: a systematic review and user contextualisation.: PROSPERO; 2019 [cited background. Available from: https://www.crd.york.ac.uk/prospero/display_record.php?ID=CRD42019147773.
15. Cochrane Effective Practice Organisation of Care (EPOC). EPOC Resources for review authors: What study designs can be considered for inclusion in an EPOC review and what should that be called? 2017 [cited background. Available from: http://epoc.cochrane.org/resources/epoc-resources-review-authors accessed April 15, 2020.
16. Cochrane Effective Practice and Organisation of Care (EPOC). Data collection form. EPOC resources for review authors 2017 [cited background. Available from: https://epoc.cochrane.org/resources/epoc-resources-review-authors accessed March 30,2020.

For peer review only - http://bmjopen.bmj.com/site/about/guidelines.xhtml

17. Higgins JPT TJ, Chandler J, Cumpston M, Li T, Page MJ, Welch VA (editors). Cochrane Handbook for Systematic Reviews of Interventions version 6.0 (updated July 2019): Cochrane 2019.
18. Cochrane Effective Practice and Organisation of Care (EPOC). Suggested risk of bias criteria for EPOC reviews. EPOC resources for review authors 2017 [cited background. Available from: https://epoc.cochrane.org/resources/epoc-resources-review-authors.
19. Borenstein M, Hedges L, Higgins J, et al. When does it make sense to perform a meta-analysis? Introduction to Meta-Analysis. Chester, UK: John Wiley & Sons Ltd 2009.
20. Beeckman D, Clays E, Van Hecke A, et al. A multi-faceted tailored strategy to implement an electronic clinical decision support system for pressure ulcer prevention in nursing homes: a two-armed randomized controlled trial. *Int J Nurs Stud* 2013;50(4):475-86. doi: https://dx.doi.org/10.1016/j.ijnurstu.2012.09.007
21. Bennett P, Hardiker N. A Quantitative Study Investigating the Effects of Computerised Clinical Decision Support in the Emergency Department. *Studies in health technology and informatics* 2016;225:53-57.
22. Blaha J, Kopecky P, Matias M, et al. Comparison of Three Protocols for Tight Glycemic Control in Cardiac Surgery Patients. *Diabetes Care* 2009;32(5):757. doi: 10.2337/dc08-1851
23. Byrne CM. Impact of prospective computerized clinical decision support information and targeted assistance on nursing home resident outcomes. State University of New York at Albany, 2005.
24. Canbolat O, Kapucu S, Kilickaya O. Comparison of Routine and Computer-Guided Glucose Management for Glycemic Control in Critically Ill Patients. *Critical Care Nurse* 2019;39(4):20-27. doi: 10.4037/ccn2019431
25. Cavalcanti AB, Silva E, Pereira AJ, et al. A randomized controlled trial comparing a computer-assisted insulin infusion protocol with a strict and a conventional protocol for glucose control in critically ill patients. *Journal of Critical Care* 2009;24(3):371-78. doi: https://doi.org/10.1016/j.jcrc.2009.05.005
26. Cleveringa FGW, Gorter KJ, Van Donk MD, et al. Combined task delegation, computerized decision support, and feedback improve cardiovascular risk for type 2 diabetic patients. *Diabetes Care* 2008;31(12):2273-75.
27. Cleveringa FGW, Welsing PMJ, Van Den Donk M, et al. Cost-effectiveness of the diabetes care protocol, a multifaceted computerized decision support diabetes management intervention that reduces cardiovascular risk. *Diabetes Care* 2010;33(2):258-63.
28. Cortez S. Measuring active clinical decision support influence on nursing research utilization. University of Phoenix, 2014.
29. Dalaba MA, Akweongo P, Aborigo RA, et al. Cost-effectiveness of clinical decision support system in improving maternal health care in Ghana. *PLoS ONE* 2015;10(5):e0125920. doi: https://dx.doi.org/10.1371/journal.pone.0125920
30. Dowding DW, Turley M, Garrido T. The impact of an electronic health record on nurse sensitive patient outcomes: an interrupted time series analysis. *J Am Med Inform Assoc* 2012;19(4):615-20. doi: https://dx.doi.org/10.1136/amiajnl-2011-000504
31. Duclos A, Touzet S, Restier L, et al. Implementation of a computerized system in pediatric wards to improve nutritional care: a cluster randomized trial. *European journal of clinical nutrition* 2015;69(7):769-75. doi: 10.1038/ejcn.2014.288
32. Dumont C, Bourguignon C. Effect of a computerized insulin dose calculator on the process of glycemic control. *Am J Crit Care* 2012;21(2):106-15. doi: https://dx.doi.org/10.4037/ajcc2012956
33. Fitzmaurice DA, Hobbs FD, Murray ET, et al. Oral anticoagulation management in primary care with the use of computerized decision support and near-patient testing: a randomized, controlled trial. *Archives of Internal Medicine* 2000;160(15):2343-8.

For peer review only - http://bmjopen.bmj.com/site/about/guidelines.xhtml

34. Forberg U, Unbeck M, Wallin L, et al. Effects of computer reminders on complications of peripheral venous catheters and nurses' adherence to a guideline in paediatric care-a cluster randomised study. *Implementation Science* 2016;11 doi: 10.1186/s13012-016-0375-9
35. Fossum M, Alexander GL, Ehnfors M, et al. Effects of a computerized decision support system on pressure ulcers and malnutrition in nursing homes for the elderly. *Int J Med Inf* 2011;80(9):607-17. doi: https://dx.doi.org/10.1016/j.ijmedinf.2011.06.009
36. Geurts D, de Vos-Kerkhof E, Polinder S, et al. Implementation of clinical decision support in young children with acute gastroenteritis: a randomized controlled trial at the emergency department. *European Journal of Pediatrics* 2017;176(2):173-81.
37. Hovorka R, Kremen J, Blaha J, et al. Blood Glucose Control by a Model Predictive Control Algorithm with Variable Sampling Rate Versus a Routine Glucose Management Protocol in Cardiac Surgery Patients: A Randomized Controlled Trial. *The Journal of Clinical Endocrinology & Metabolism* 2007;92(8):2960-64. doi: 10.1210/jc.2007-0434
38. Kroth PJ, Dexter PR, Overhage JM, et al. A computerized decision support system improves the accuracy of temperature capture from nursing personnel at the bedside. *Amia Annual symposium proceedings AMIA symposium* 2006:444-48.
39. Lattimer V, George S, Thompson F, et al. Safety and effectiveness of nurse telephone consultation in out of hours primary care: Randomised controlled trial. *British Medical Journal* 1998;317(7165):1054-59.
40. Lattimer V, Sassi F, George S, et al. Cost analysis of nurse telephone consultation in out of hours primary care: evidence from a randomised controlled trial. *BMJ* 2000;320(7241):1053-7.
41. Lee NJ, Chen ES, Currie LM, et al. The effect of a mobile clinical decision support system on the diagnosis of obesity and overweight in acute and primary care encounters. *ANS Adv Nurs Sci* 2009;32(3):211-21. doi: https://dx.doi.org/10.1097/ANS.0b013e3181b0d6bf
42. Mann EA, Jones JA, Wolf SE, et al. Computer decision support software safely improves glycemic control in the burn intensive care unit: A randomized controlled clinical study. *Journal of Burn Care and Research* 2011;32(2):246-55.
43. McDonald MV, Feldman PH, Barron-Vaya Y, et al. Outcomes of clinical decision support (CDS) and correlates of CDS use for home care patients with high medication regimen complexity: a randomized trial. *Journal of Evaluation in Clinical Practice* 2017;22(1):10-19.
44. Plank J, Blaha J, Cordingley J, et al. Multicentric, Randomized, Controlled Trial to Evaluate Blood Glucose Control by the Model Predictive Control Algorithm Versus Routine Glucose Management Protocols in Intensive Care Unit Patients. *Diabetes Care* 2006;29(2):271. doi: 10.2337/diacare.29.02.06.dc05-1689
45. Rood E, Bosman RJ, van der Spoel JI, et al. Use of a Computerized Guideline for Glucose Regulation in the Intensive Care Unit Improved Both Guideline Adherence and Glucose Regulation. *Journal of the American Medical Informatics Association* 2005;12(2):172-80. doi: 10.1197/jamia.M1598
46. Roukema J, Steyerberg EW, van der Lei J, et al. Randomized trial of a clinical decision support system: impact on the management of children with fever without apparent source. *Journal of the american medical informatics association : JAMIA* 2008;15(1):107-13. doi: 10.1197/jamia.M2164
47. Sassen B, Kok G, Schepers J, et al. Supporting health care professionals to improve the processes of shared decision making and self-management in a web-based intervention: randomized controlled trial. *Journal of medical Internet research* 2014;16(10):e211.
48. Snooks HA, Carter B, Dale J, et al. Support and Assessment for Fall Emergency Referrals (SAFER 1): cluster randomised trial of computerised clinical decision support for paramedics. *PLoS ONE* 2014;9(9):e106436. doi: https://dx.doi.org/10.1371/journal.pone.0106436
49. Vadher BD, Patterson DL, Leaning M. Comparison of oral anticoagulant control by a nurse-practitioner using a computer decision-support system with that by clinicians. *Clinical and laboratory haematology* 1997;19(3):203-07.

For peer review only - http://bmjopen.bmj.com/site/about/guidelines.xhtml

50. Wells B. Implementation of Computerised Clinical Decision Support (CCDS) in a Prehospital Setting: Processes of Adoption and Impact on Paramedic Role and Practice. Swansea University (United Kingdom), 2013.
51. Dykes PC, Carroll DL, Hurley A, et al. Fall prevention in acute care hospitals: a randomized trial. *Jama* 2010;304(17):1912-18.
52. Paulsen MM, Paur I, Gjestland J, et al. Effects of using the MyFood decision support system on hospitalized patients' nutritional status and treatment: A randomized controlled trial. *Clinical Nutrition* 2020;39(12):3607-17.
53. Lv S, Ye X, Wang Z, et al. A randomized controlled trial of a mobile application-assisted nurse-led model used to improve treatment outcomes in children with asthma. *Journal of advanced nursing* 2019;75(11):3058-67.
54. Dykes PC, Burns Z, Adelman J, et al. Evaluation of a patient-centered fall-prevention tool kit to reduce falls and injuries: a nonrandomized controlled trial. *JAMA network open* 2020;3(11):e2025889-e89.
55. National Institute for Health and Clinical Excellence (NICE). Guide to the methods of technology appraisal London2013 [Available from: https://www.nice.org.uk/process/pmg9/chapter/the-appraisal-of-the-evidence-and-structured-decision-making accessed November 18,2020.
56. Murray E, Treweek S, Pope C, et al. Normalisation process theory: a framework for developing, evaluating and implementing complex interventions. *BMC medicine* 2010;8(1):1-11.
57. Greenhalgh T, Wherton J, Papoutsi C, et al. Beyond adoption: a new framework for theorizing and evaluating nonadoption, abandonment, and challenges to the scale-up, spread, and sustainability of health and care technologies. *Journal of medical Internet research* 2017;19(11):e367.
58. Kawamoto K, McDonald CJ. Designing, conducting, and reporting clinical decision support studies: recommendations and call to action. *Annals of Internal Medicine* 2020;172(11_Supplement):S101-S09. doi: https://doi.org/10.7326/M19-0875
59. National Health Services (NHS). Digital Health: NHSX launches assessment criteria for digital health tools 2020 [cited discussion. Available from: https://www.digitalhealth.net/2020/10/nhsx-launches-assessment-criteria-for-digital-health-tools/ accessed October 29, 2020.

For peer review only - http://bmjopen.bmj.com/site/about/guidelines.xhtml

Figure 1 PRISMA Flow chart of study selection process

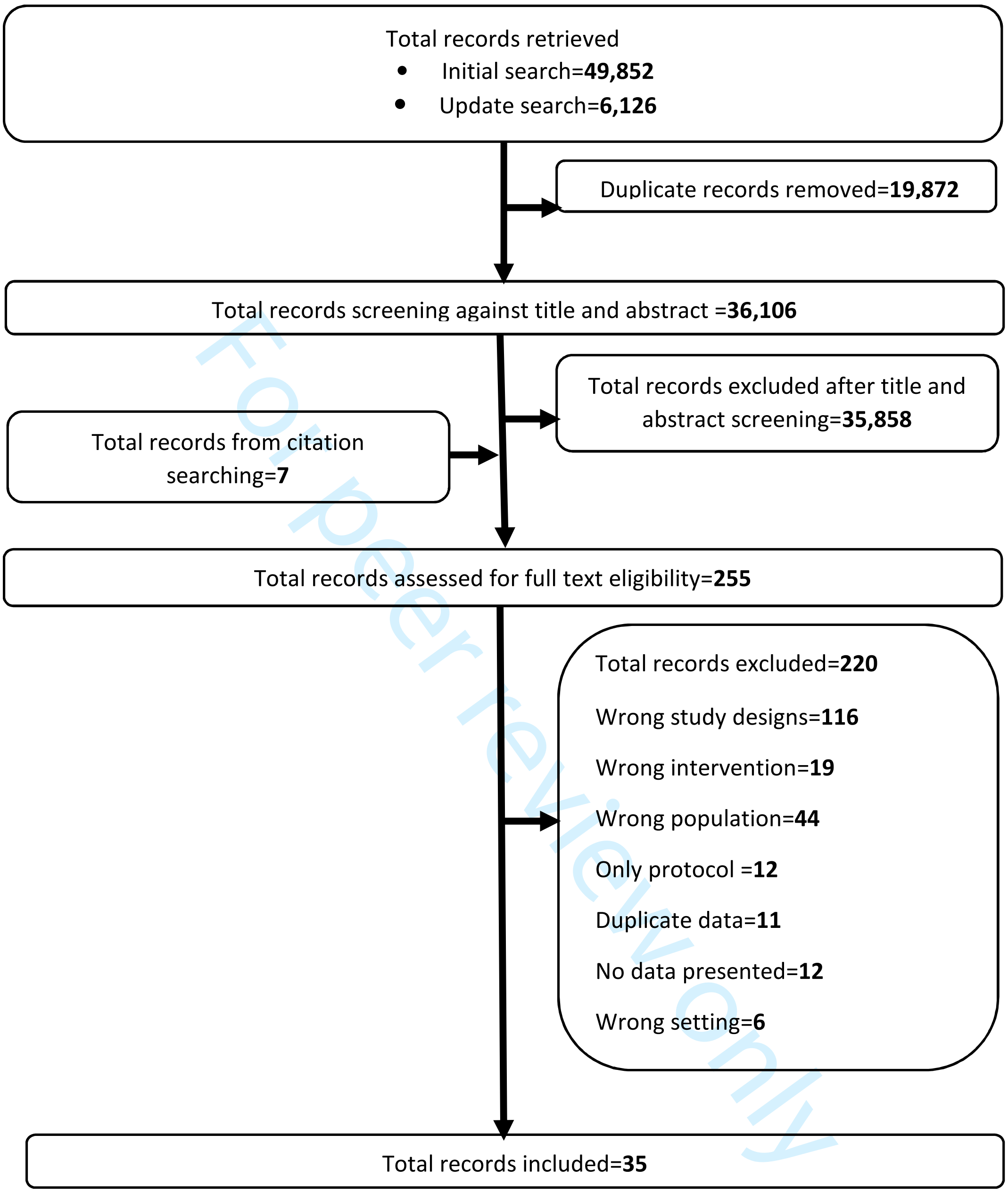

For peer review only

SUPPLEMENTARY MATERIAL LIST

Supplementary Table 1: Search strategies, to February 12, 2021

Supplementary Table 2: Risk of Bias assessment justifications using Effective Practice Organisation of Care (EPOC)'s tool

Supplementary Table 3: Summary of patient care process results

Supplementary Table 4 Summary of patient care outcomes results

Supplementary Table 5: Summary of Health professionals' knowledge, beliefs and behaviour results

Supplementary Table 6: Summary of adverse events results

Supplementary Table 7: Summary of economic costs and consequences results

For peer review only

Supplementary Table 1: Search strategies

1. **Ovid MEDLINE(R) ALL, 1946 to February 12, 2021 Search Strategy**

----

1 exp Decision Making/ (207895)

2 decision support techniques/ (20911)

3 (decision* adj2 making).ti,ab,kf. (159754)

4 (decision* adj2 support*).ti,ab,kf. (24230)

5 (decision* adj2 aid*).ti,ab,kf. (6501)

6 or/1-5 (354546)

7 exp Computers/ (79322)

8 exp information systems/ (238259)

9 exp Informatics/ (537355)

10 Internet/ (74916)

11 Software/ (112580)

12 Cell Phone/ (8821)

13 Mobile Applications/ (6962)

14 exp Telemedicine/ (32559)

15 Medical Records Systems, Computerized/ (19076)

16 exp Electronic Health Records/ (21793)

17 computer*.ti,ab,kf. (313610)

18 electronic*.ti,ab,kf. (291368)

19 (internet or web or online or on-line).ti,ab,kf. (310071)

20 (software or computer program*).ti,ab,kf. (193359)

21 (automate* or automation).ti,ab,kf. (136436)

22 (pda or pdas).ti,ab,kf. (13229)

23 personal digital assistant*.ti,ab,kf. (1012)

24 (app or apps).ti,ab,kf. (31717)

25 (application* adj2 mobile*).ti,ab,kf. (4834)

26 (iPad* or iPhone* or smartphone* or smart phone* or smart device* or mobile phone or android phone* or cellphone* or cell phone*).ti,ab,kf. (26450)

27 (tablet adj2 (pc or device* or comput*)).ti,ab,kf. (1603)

28 ((hand held or handheld) adj2 (pc or device* or comput*)).ti,ab,kf. (2669)

29 (telehealth or telecare or telemedicine or ehealth or mhealth).ti,ab,kf. (29130)

30 or/7-29 (1674343)

31 6 and 30 (66042)

32 exp Decision Making, Computer-Assisted/ (149528)

33 Decision Support Systems, Clinical/ (8302)

34 (computer assisted adj2 (decision* or diagnos* or therap* or support or treatment? or management)).ti,ab,kf. (1545)

35 (computer aided adj2 (decision* or diagnos* or therap* or support or treatment? or management)).ti,ab,kf. (3921)

36 (decision adj2 support adj2 (system* or tool*)).ti,ab,kf. (9917)

37 (decision making adj2 (system* or tool*)).ti,ab,kf. (2560)

38 Expert Systems/ (3420)

39 (expert adj2 system*).ti,ab,kf. (3613)

40 Reminder Systems/ (3568)

41 ((computer* or electronic* or CDSS) adj2 (reminder* or alert*)).ti,ab,kf. (1210)

42 ((medication or medicine or treatment or therapy) adj2 (reminder* or alert*)).ti,ab,kf. (857)

43 reminder system*.ti,ab,kf. (875)

44 Medical Order Entry Systems/ (2303)

45 ((computer* or electronic*) adj2 order entry).ti,ab,kf. (1874)

46 (computer adj2 decision support*).ti,ab. (412)

47 CPOE.ti,ab,kf. (1139)

48 or/32-47 (177952)

49 31 or 48 [all computerised clinical decision support systems terms] (228840)

50 Allied Health Personnel/ (11925)

51 Allied Health Occupations/ (587)

52 Physical Therapist Assistants/ (16)

53 Physical Therapy Specialty/ (2889)

54 Speech-Language Pathology/ (3172)

For peer review only

55 Occupational Therapy/ (13482)

56 Nutritionists/ (1290)

57 dietetics/ (7837)

58 Anesthesiologists/ (1163)

59 podiatry/ (2273)

60 exp Osteopaths/ (321)

61 osteopathic physicians/ (321)

62 anesthesiologist*.ti,ab,kf. (22810)

63 podiatrist*.ti,ab,kf. (910)

64 prosthetist*.ti,ab,kf. (397)

65 chiropodist*.ti,ab,kf. (132)

66 orthoptist*.ti,ab,kf. (319)

67 orthotist*.ti,ab,kf. (220)

68 osteopath*.ti,ab,kf. (5983)

69 radiographer*.ti,ab,kf. (1803)

70 art therapist*.ti,ab,kf. (89)

71 drama therapist*.ti,ab,kf. (3)

72 music therapist*.ti,ab,kf. (368)

73 (allied adj2 health adj2 (profession* or worker* or personnel or occupation* or staff)).ti,ab,kf. (3421)

74 ((physical or occupational or language or speech or physio*) adj2 therap*).ti,ab,kf. (50227)

75 physiotherapist*.ti,ab,kf. (8544)

76 dietetic*.ti,ab,kf. (9828)

77 dietitian*.ti,ab,kf. (6580)

78 nutritionist*.ti,ab,kf. (3020)

79 Patient care team/ (66483)

80 ((multidisciplinary or multi-disciplinary or multiprofessional or multi-professional or interdisciplinary or interprofessional) adj2 team*).ti,ab,kf. (32126)

81 Emergency Medical Technicians/ (5756)

82 Emergency Medical Services/ (43736)

83 Ambulances/ (6210)

For peer review only

84 Air Ambulances/ (2874)

85 paramedic*.ti,ab,kf. (8537)

86 HEMS.ti,ab,kf. (767)

87 ems.ti,ab,kf. (13017)

88 emt.ti,ab,kf. (25232)

89 prehospital.ti,ab,kf. (13136)

90 pre-hospital.ti,ab,kf. (4836)

91 first responder*.ti,ab,kf. (2449)

92 emergency medical technician*.ti,ab,kf. (1168)

93 emergency services.ti,ab,kf. (4115)

94 ambulance*.ti,ab,kf. (11269)

95 field triage.ti,ab,kf. (275)

96 out-of-hospital.ti,ab,kf. (11317)

97 (nurse or nurses or nursing).ti,ab,kf. (462330)

98 exp nurses/ (89638)

99 exp nursing staff/ (67063)

100 Midwifery/ (19460)

101 (midwif* or midwiv*).ti,ab,kf. (25895)

102 or/50-101 [allied health professionals or nurses or midwives] (836031)

103 49 and 102 [all CDSS and allied health professionals or nurses or midwives] (9549)

2. Embase Classic+Embase 1947 to February 12, 2021 Search Strategy

----

1 exp Decision Making/ (399525)

2 decision support techniques/ (20092)

3 (decision* adj2 making).ti,ab,kw. (218454)

4 (decision* adj2 support*).ti,ab,kw. (32940)

5 (decision* adj2 aid*).ti,ab,kw. (9487)

6 or/1-5 (504731)

7 exp Computer/ (159861)

For peer review only

8 exp information system/ (166084)

9 exp information science/ (113984)

10 Internet/ (112888)

11 Software/ (79162)

12 mobile phone/ (17899)

13 smartphone/ (15041)

14 Mobile Application/ (13261)

15 exp Telemedicine/ (47236)

16 electronic medical record system/ (1535)

17 exp Electronic Health Record/ (21723)

18 computer*.ti,ab,kw. (407323)

19 electronic*.ti,ab,kw. (350647)

20 (internet or web or online or on-line).ti,ab,kw. (418206)

21 (software or computer program*).ti,ab,kw. (321717)

22 (automate* or automation).ti,ab,kw. (197239)

23 (pda or pdas).ti,ab,kw. (18450)

24 personal digital assistant*.ti,ab,kw. (1217)

25 (app or apps).ti,ab,kw. (43764)

26 (application* adj2 mobile*).ti,ab,kw. (6399)

27 (iPad* or iPhone* or smartphone* or smart phone* or smart device* or android phone* or cellphone* or cell phone* or mobile phone*).ti,ab,kw. (38430)

28 (tablet adj2 (pc or device* or comput*)).ti,ab,kw. (2528)

29 ((hand held or handheld) adj2 (pc or device* or comput*)).ti,ab,kw. (3833)

30 (telehealth or telecare or telemedicine or ehealth or mhealth).ti,ab,kw. (35247)

31 or/7-30 (1897765)

32 6 and 31 (80108)

33 exp decision support system/ (27016)

34 clinical decision support system/ (3594)

35 (computer assisted adj2 (decision* or diagnos* or therap* or support or treatment? or management)).ti,ab,kw. (2316)

36 (computer aided adj2 (decision* or diagnos* or therap* or support or treatment? or management)).ti,ab,kw. (5577)

37 (decision adj2 support adj2 (system* or tool*)).ti,ab,kw. (13211)

38 (decision making adj2 (system* or tool*)).ti,ab,kw. (3662)

39 Expert System/ (5507)

40 (expert adj2 system*).ti,ab,kw. (5205)

41 Reminder System/ (2730)

42 ((computer* or electronic* or CDSS) adj2 (reminder* or alert*)).ti,ab,kw. (1848)

43 ((medication or medicine or treatment or therapy) adj2 (reminder* or alert*)).ti,ab. (1362)

44 reminder system*.ti,ab,kw. (1189)

45 physician order entry system/ (284)

46 ((computer* or electronic*) adj2 order entry).ti,ab,kw. (2801)

47 CPOE.ti,ab,kw. (1715)

48 (computer* adj2 decision support*).ti,ab,kw. (1907)

49 or/33-48 (56905)

50 32 or 49 [All computerised clinical decision support systems terms] (106747)

51 Occupation/ (52894)

52 physiotherapist assistant/ (83)

53 physiotherapist/ (23150)

54 speech disorder/ (27422)

55 Occupational Therapy/ (25731)

56 dietitian/ (13219)

57 Anesthesiologist/ (7231)

58 osteopathic physician/ (356)

59 radiographer/ (634)

60 podiatrist/ (831)

61 anesthesiologist*.ti,ab,kw. (34979)

62 podiatrist*.ti,ab,kw. (1315)

63 prosthetist*.ti,ab,kw. (635)

64 chiropodist*.ti,ab,kw. (179)

For peer review only

65 orthoptist*.ti,ab,kw. (620)

66 orthotist*.ti,ab,kw. (419)

67 osteopath*.ti,ab,kw. (8365)

68 radiographer*.ti,ab,kw. (4001)

69 art therapist*.ti,ab,kw. (266)

70 drama therapist*.ti,ab,kw. (20)

71 music therapist*.ti,ab,kw. (607)

72 (allied adj2 health adj2 (profession* or worker* or personnel or occupation* or staff)).ti,ab,kw. (5338)

73 ((physical or physio* or occupational or language or speech) adj2 therap*).ti,ab,kw. (77705)

74 physiotherapist*.ti,ab,kw. (18271)

75 dietetic*.ti,ab,kw. (14409)

76 dietitian*.ti,ab,kw. (10785)

77 nutritionist*.ti,ab,kw. (5156)

78 Patient care/ (310700)

79 multi-disciplinary team/ (10246)

80 collaborative care team/ (903)

81 ((multidisciplinary or multi-disciplinary or multiprofessional or multi-professional or interdisciplinary or interprofessional) adj2 team*).ti,ab,kw. (57679)

82 rescue personnel/ (8059)

83 emergency health service/ (105109)

84 ambulance/ (14751)

85 air medical transport/ (2965)

86 paramedical personnel/ (14896)

87 paramedic*.ti,ab,kw. (13029)

88 HEMS.ti,ab,kw. (1067)

89 ems.ti,ab,kw. (19120)

90 emt.ti,ab,kw. (36500)

91 prehospital.ti,ab,kw. (18282)

92 pre-hospital.ti,ab,kw. (8656)

93 first responder*.ti,ab,kw. (3260)

For peer review only

94 emergency medical technician*.ti,ab,kw. (1553)

95 emergency services.ti,ab,kw. (6114)

96 ambulance*.ti,ab,kw. (17409)

97 field triage.ti,ab,kw. (382)

98 out-of-hospital.ti,ab,kw. (19034)

99 (nurse or nurses or nursing).ti,ab,kw. (554357)

100 exp nurse/ (194823)

101 nursing staff/ (73869)

102 midwife/ (28233)

103 (midwif* or midwiv*).ti,ab. (29459)

104 or/51-103 [allied health professionals or nurses or midwives] (1389786)

105 50 and 104 [all CDSS and allied health professionals or nurses or midwives] (16820)

3. PsycINFO 1806 to February 12,2021 Search Strategy:

1 exp Decision Making/ (124412)

2 Decision Support Systems/ (3377)

3 (decision* adj2 making).ti,ab. (93578)

4 (decision* adj2 support*).ti,ab. (5773)

5 (decision* adj2 aid*).ti,ab. (1934)

6 or/1-5 (168090)

7 exp Computers/ (43893)

8 exp information systems/ (48548)

9 exp information/ (44565)

10 Internet/ (29404)

11 computer software/ (10412)

12 mobile Phones/ (4735)

13 smartphones/ (1843)

14 mobile applications/ (1082)

15 Mobile devices/ (2634)

16 exp Telemedicine/ (9383)

For peer review only

17 Health Information Technology/ (304)

18 Electronic Health Records/ (880)

19 computer*.ti,ab. (91287)

20 electronic*.ti,ab. (33377)

21 (internet or web or online or on-line).ti,ab. (145714)

22 (software or computer program*).ti,ab. (31224)

23 (automate* or automation).ti,ab. (14470)

24 (pda or pdas).ti,ab. (937)

25 personal digital assistant*.ti,ab. (440)

26 (app or apps).ti,ab. (7624)

27 (application* adj2 mobile*).ti,ab. (1392)

28 (iPad* or iPhone* or mobile phone or smartphone* or smart phone* or smart device* or android phone* or cellphone* or cell phone*).ti,ab. (10036)

29 (tablet adj2 (pc or device* or comput*)).ti,ab. (680)

30 ((hand held or handheld) adj2 (pc or device* or comput*)).ti,ab. (813)

31 (telehealth or telecare or telemedicine or ehealth or mhealth).ti,ab. (4475)

32 or/7-31 (362180)

33 6 and 32 (21605)

34 Decision Support Systems/ (3377)

35 Computer Assisted Diagnosis/ (1589)

36 (computer assisted adj2 (decision* or diagnos* or therap* or support or treatment? or management)).ti,ab. (273)

37 (computer aided adj2 (decision* or diagnos* or therap* or support or treatment? or management)).ti,ab. (179)

38 (decision adj2 support adj2 (system* or tool*)).ti,ab. (2189)

39 (decision making adj2 (system* or tool*)).ti,ab. (1022)

40 Expert Systems/ (5732)

41 (expert adj2 system*).ti,ab. (1376)

42 ((medication or medicine or treatment or therapy) adj2 (reminder* or alert*)).ti,ab. (202)

43 reminder system*.ti,ab. (125)

For peer review only

44 ((computer* or electronic*) adj2 order entry).ti,ab. (94)

45 (computer* adj2 decision support*).ti,ab. (183)

46 CPOE.ti,ab. (46)

47 or/33-46 [CDSS terms] (30902)

48 Allied Health Personnel/ (1109)

49 Physical Therapists/ (536)

50 Physical Therapy/ (2987)

51 Speech therapists/ (1229)

52 Speech Language Pathology/ (1088)

53 Occupational Therapists/ (2346)

54 anesthesiologist*.ti,ab. (457)

55 podiatrist*.ti,ab. (47)

56 prosthetist*.ti,ab. (23)

57 orthoptist*.ti,ab. (17)

58 [chiropodist*.ti,kw.] (0)

59 [orthotist*.ti,kw.] (0)

60 [osteopath*.ti,kw.] (0)

61 radiographer*.ti,ab. (81)

62 art therapist*.ti,ab. (1375)

63 drama therapist*.ti,ab. (75)

64 music therapist*.ti,ab. (1337)

65 (allied adj2 health adj2 (profession* or worker* or personnel or occupation* or staff)).ti,ab. (1123)

66 ((physical or physio* or occupational or language or speech) adj2 therap*).ti,ab. (18118)

67 physiotherapist*.ti,ab. (1346)

68 dietetic*.ti,ab. (610)

69 dietitian*.ti,ab. (756)

70 nutritionist*.ti,ab. (417)

71 Interdisciplinary Treatment Approach/ (7399)

72 ((multidisciplinary or multi-disciplinary or multiprofessional or multi-professional or interdisciplinary or interprofessional) adj2 team*).ti,ab. (8106)

For peer review only

73 emergency services/ (8779)

74 emergency personnel/ (117)

75 paramedics/ (337)

76 HEMS.ti,ab. (27)

77 ems.ti,ab. (1010)

78 emt.ti,ab. (230)

79 prehospital.ti,ab. (387)

80 pre-hospital.ti,ab. (262)

81 first responders/ (307)

82 emergency medical technician*.ti,ab. (154)

83 emergency services.ti,ab. (1211)

84 ambulance*.ti,ab. (860)

85 field triage.ti,ab. (6)

86 out-of-hospital.ti,ab. (355)

87 exp nurses/ (32673)

88 nursing/ (23241)

89 (nurse or nurses or nursing).ti,ab. (97190)

90 midwifery/ (1436)

91 (midwif* or midwiv*).ti,ab. (3137)

92 or/48-91 [allied health professionals or nurses or midwives] (148809)

93 47 and 92 [all CDSS and allied health professionals or nurses or midwives] (1171)

4. Database: HMIC Health Management Information Consortium 1983 – February 12, 2021

Search Strategy:

---

1 exp Decision Making/ (5606)

2 (decision* adj2 making).ti,ab. (6795)

3 (decision* adj2 support*).ti,ab. (871)

4 (decision* adj2 aid*).ti,ab. (276)

5 or/1-4 (10211)

6 exp Computers/ (2133)

7 exp information systems/ (4916)

For peer review only

8 exp medical Informatics/ (67)

9 Internet/ (1342)

10 Software/ (0)

11 telephone/ (110)

12 Telemedicine/ (1328)

13 computerised medical records systems.ti,ab. (0)

14 Medical Records/ (1946)

15 computer*.ti,ab. (6305)

16 electronic*.ti,ab. (4484)

17 (internet or web or online or on-line).ti,ab. (5066)

18 (software or computer program*).ti,ab. (1593)

19 (automate* or automation).ti,ab. (605)

20 (pda or pdas).ti,ab. (56)

21 personal digital assistant*.ti,ab. (32)

22 (app or apps).ti,ab. (130)

23 (application* adj2 mobile*).ti,ab. (32)

24 (iPad* or iPhone* or smartphone* or smart phone* or smart device* or android phone* or cellphone* or cell phone*).ti,ab. (146)

25 (tablet adj2 (pc or device* or comput*)).ti,ab. (16)

26 ((hand held or handheld) adj2 (pc or device* or comput*)).ti,ab. (61)

27 (telehealth or telecare or telemedicine or mhealth or ehealth).ti,ab. (1453)

28 or/6-27 (22729)

29 5 and 28 (1239)

30 (computer assisted adj2 (decision* or diagnos* or therap* or support or treatment? or management)).ti,ab. (25)

31 (computer aided adj2 (decision* or diagnos* or therap* or support or treatment? or management)).ti,ab. (17)

32 (decision adj2 support adj2 (system* or tool*)).ti,ab. (347)

33 (decision making adj2 (system* or tool*)).ti,ab. (107)

34 Expert Systems/ (107)

35 (expert adj2 system*).ti,ab. (131)

For peer review only

36 ((computer* or electronic* or CDSS) adj2 (reminder* or alert*)).ti,ab. (48)

37 reminder system*.ti,ab. (44)

38 ((computer* or electronic* or CDSS) adj2 (reminder* or alert*)).ti,ab. (48)

39 ((computer* or electronic*) adj2 order entry).ti,ab. (58)

40 (computer* adj2 decision support*).ti,ab. (114)

41 CPOE.ti,ab. (26)

42 or/29-41 [all CDSS terms] (1714)

43 Allied Health Personnel/ (0)

44 Physical Therapy Speciality/ (0)

45 Physiotherapists/ (350)

46 Speech-Language Pathology/ (0)

47 Occupational Therapists/ (542)

48 podiatrists/ (59)

49 anesthesiologist*.ti,ab. (11)

50 podiatrist*.ti,ab. (37)

51 prosthetist*.ti,ab. (19)

52 chiropodist*.ti,ab. (76)

53 orthoptist*.ti,ab. (23)

54 orthotist*.ti,ab. (15)

55 osteopath*.ti,ab. (93)

56 radiographer*.ti,ab. (178)

57 art therapist*.ti,ab. (5)

58 drama therapist*.ti,ab. (2)

59 music therapist*.ti,tw. (15)

60 (allied adj2 health adj2 (profession* or worker* or personnel or occupation* or staff)).ti,ab. (368)

61 ((physical or physio* or occupational or language or speech) adj2 therap*).ti,ab. (2010)

62 physiotherapist*.ti,ab. (671)

63 dietetic*.ti,ab. (187)

64 dietitian*.ti,ab. (130)

For peer review only

65 nutritionist*.ti,ab. (28)

66 Patient care team/ (139)

67 ((multidisciplinary or multi-disciplinary or multiprofessional or multi-professional or interdisciplinary or interprofessional) adj2 team*).ti,ab. (1676)

68 exp emergency medical services/ (0)

69 paramedic*.ti,ab. (395)

70 HEMS.ti,ab. (11)

71 ems.ti,ab. (51)

72 emt.ti,ab. (3)

73 prehospital.ti,ab. (58)

74 pre-hospital.ti,ab. (137)

75 first responder*.ti,ab. (28)

76 emergency medical technician*.ti,ab. (8)

77 emergency services.ti,ab. (514)

78 ambulance*.ti,ab. (1710)

79 field triage.ti,ab. (1)

80 out-of-hospital.tw. (292)

81 nurses/ (12920)

82 nursing staff/ (12920)

83 (nurse or nurses or nursing).ti,ab. (39541)

84 midwifery/ (665)

85 (midwif* or midwiv*).ti,ab. (4553)

86 or/43-85 [allied health professionals or nurses or midwives] (50288)

87 42 and 86 [all CDSS terms and allied health professionals or nurses or midwives] (291)

5. AMED (Allied and Complementary Medicine) 1985 to October 2019Search Strategy:

1 exp Decision Making/ (4522)

2 (decision* adj2 making).ti,ab. (2826)

3 (decision* adj2 support*).ti,ab. (217)

For peer review only

4 (decision* adj2 aid*).ti,ab. (92)

5 or/1-4 (6218)

6 exp Computers/ (1765)

7 exp information systems/ (150)

8 exp medical Informatics/ (775)

9 Internet/ (1242)

10 Software/ (450)

11 telephone/ (377)

12 Telemedicine/ (985)

13 computerised medical records systems.ti,ab. (0)

14 Medical Records/ (383)

15 computer*.ti,ab. (4200)

16 electronic*.ti,ab. (2339)

17 (internet or web or online or on-line).ti,ab. (6503)

18 (software or computer program*).ti,ab. (1436)

19 (automate* or automation).ti,ab. (399)

20 (pda or pdas).ti,ab. (77)

21 personal digital assistant*.ti,ab. (26)

22 (app or apps).ti,ab. (175)

23 (application* adj2 mobile*).ti,ab. (39)

24 (iPad* or iPhone* or smartphone* or smart phone* or smart device* or android phone* or cellphone* or cell phone*).ti,ab. (225)

25 (tablet adj2 (pc or device* or comput*)).ti,ab. (29)

26 ((hand held or handheld) adj2 (pc or device* or comput*)).ti,ab. (40)

27 (telehealth or telecare or telemedicine or mhealth or ehealth).ti,ab. (555)

28 or/6-27 (16500)

29 5 and 28 (443)

30 (computer assisted adj2 (decision* or diagnos* or therap* or support or treatment? or management)).ti,ab. (18)

31 (computer aided adj2 (decision* or diagnos* or therap* or support or treatment? or management)).ti,ab. (13)

32 (decision adj2 support adj2 (system* or tool*)).ti,ab. (41)

33 (decision making adj2 (system* or tool*)).ti,ab. (62)

34 Expert Systems/ (12)

35 (expert adj2 system*).ti,ab. (46)

36 ((computer* or electronic* or CDSS) adj2 (reminder* or alert*)).ti,ab. (7)

37 reminder system*.ti,ab. (3)

38 ((computer* or electronic* or CDSS) adj2 (reminder* or alert*)).ti,ab. (7)

39 ((computer* or electronic*) adj2 order entry).ti,ab. (0)

40 (computer* adj2 decision support*).ti,ab. (8)

41 CPOE.ti,ab. (0)

42 or/29-41 [all CDSS terms] (593)

43 Allied Health Personnel/ (659)

44 Physical Therapy Speciality/ (2201)

45 Physiotherapists/ (1476)

46 Speech-Language Pathology/ (237)

47 Occupational Therapists/ (1076)

48 podiatrists/ (36)

49 anesthesiologist*.ti,ab. (64)

50 podiatrist*.ti,ab. (172)

51 prosthetist*.ti,ab. (84)

52 chiropodist*.ti,ab. (32)

53 orthoptist*.ti,ab. (1)

54 orthotist*.ti,ab. (63)

55 osteopath*.ti,ab. (1733)

56 radiographer*.ti,ab. (18)

57 art therapist*.ti,ab. (179)

58 drama therapist*.ti,ab. (10)

59 music therapist*.ti,tw. (115)

60 (allied adj2 health adj2 (profession* or worker* or personnel or occupation* or staff)).ti,ab. (285)

61 ((physical or physio* or occupational or language or speech) adj2 therap*).ti,ab. (14459)

For peer review only

62 physiotherapist*.ti,ab. (2897)

63 dietetic*.ti,ab. (133)

64 dietitian*.ti,ab. (74)

65 nutritionist*.ti,ab. (39)

66 Patient care team/ (1786)

67 ((multidisciplinary or multi-disciplinary or multiprofessional or multi-professional or interdisciplinary or interprofessional) adj2 team*).ti,ab. (1129)

68 exp emergency medical services/ (420)

69 paramedic*.ti,ab. (78)

70 HEMS.ti,ab. (1)

71 ems.ti,ab. (96)

72 emt.ti,ab. (65)

73 prehospital.ti,ab. (32)

74 pre-hospital.ti,ab. (13)

75 first responder*.ti,ab. (9)

76 emergency medical technician*.ti,ab. (8)

77 emergency services.ti,ab. (24)

78 ambulance*.ti,ab. (45)

79 field triage.ti,ab. (0)

80 out-of-hospital.tw. (10429)

81 nurses/ (1071)

82 nursing staff/ (213)

83 (nurse or nurses or nursing).ti,ab. (9441)

84 midwifery/ (120)

85 (midwif* or midwiv*).ti,ab. (239)

86 or/43-85 [allied health professionals or nurses or midwives] (41793)

87 42 and 86 [all CDSS terms and allied health professionals or nurses or midwives] (186)

6. CINAHL EBSCO Search Strategy

For peer review only

#	Query*	Results
S101	S46 AND S100	11,824
S100	S47 OR S48 OR S49 OR S50 OR S51 OR S52 OR S53 OR S54 OR S55 OR S56 OR S57 OR S58 OR S59 OR S60 OR S61 OR S62 OR S63 OR S64 OR S65 OR S66 OR S67 OR S68 OR S69 OR S70 OR S71 OR S72 OR S73 OR S74 OR S75 OR S76 OR S77 OR S78 OR S79 OR S80 OR S81 OR S82 OR S83 OR S84 OR S85 OR S86 OR S87 OR S88 OR S89 OR S90 OR S91 OR S92 OR S93 OR S94 OR S95 OR S96 OR S97 OR S98 OR S99	867,856
S99	TI ((midwif* or midwiv*)) OR AB ((midwif* or midwiv*))	35,031
S98	(MH "Midwives+")	15,748
S97	(MH "Midwifery+")	20,976
S96	TI (((nurse or nurses or nursing)) OR ((nurse or nurses or nursing))) OR AB (((nurse or nurses or nursing)) OR ((nurse or nurses or nursing)))	535,366
S95	(MH "Nursing Staff, Hospital") "	20,953
S94	(MH "Nurses+")	228,583
S93	TI "music therapist*" OR AB "music therapist*"	592
S92	TI "drama therapist*" OR AB "drama therapist*"	6
S91	TI "art therapist*" OR AB "art therapist*"	420
S90	TI radiographer* OR AB radiographer*	2,300
S89	TI osteopath* OR AB osteopath*	3,074
S88	TI orthotist* OR AB orthotist*	188
S87	TI orthoptist* OR AB orthoptist*	34
S86	TI chiropodist* OR AB chiropodist*	458
S85	TI prosthetist* OR AB prosthetist*	335
S84	TI podiatrist* OR AB podiatrist*	2,440
S83	TI anesthesiologist* OR AB anesthesiologist*	6,441
S82	(MH "Radiologic Technologists")	5,733
S81	(MH "Osteopaths")	682
S80	(MH "Podiatrists")	2,444
S79	MH "Anesthesiologists")	1,495

For peer review only

S78	TI "out-of-hospital" OR AB "out-of-hospital"	6,634
S77	TI "field triage" OR AB "field triage"	173
S76	TI ambulance* OR AB ambulance*	6,499
S75	TI "emergency services" OR AB "emergency services"	1,921
S74	TI "emergency medical technician*" OR AB "emergency medical technician*"	725
S73	"first responder*" OR AB "first responder*"	1,402
S72	TI pre-hospital OR AB pre-hospital	2,500
S71	TI prehospital OR AB prehospital	7,480
S70	TI emt OR AB emt	2,753
S69	TI EMS OR AB EMS	9,336
S68	TI HEMS OR AB HEMS	1,348
S67	TI paramedic* OR AB paramedic*	5,903
S66	(MH "Ambulances")	4,565
S65	(MH "Emergency Medical Services")	26,747
S64	(MH "Emergency Medical Technicians")	12,426
S63	TI (((multidisciplinary or multi-disciplinary or multiprofessional or "multi-professional" or interdisciplinary or interprofessional)) OR AB ((multidisciplinary or "multi-disciplinary" or multiprofessional or "multi-professional" or interdisciplinary or interprofessional) N2 team*))	33,294
S62	(MH "Multidisciplinary Care Team")	45,878
S61	TI nutritionist* OR AB nutritionist*	1,676
S60	TI dietitian* OR AB dietitian*	5,004
S59	TI physiotherapist* OR AB physiotherapist*	8,379
S58	TI (((physical or occupational or language or speech) N1 therapist*)) AND AB (((physical or occupational or language or speech) N1 therapist*))	2,999
S57	TI ((allied N2 health N2 (profession* or worker* or personnel or occupation* or staff))) OR AB ((allied N2 health N2 (profession* or worker* or personnel or occupation* or staff)))	2,748
S56	(MH "Dietetics")	2,356

For peer review only

S55	(MH "Nutrition Services")	1,054
S54	(MH "Occupational Therapy")	23,116
S53	(MH "Speech-Language Pathology")	6,105
S52	(MH "Physical Therapists")	12,660
S51	(MH "Physical Therapy")	35,365
S50	(MH "Physical Therapist Assistants")	814
S49	TI "music therapist*" OR AB "music therapist*"	592
S48	TI "Physical Therapist Assistant*" or AB "Physical Therapist Assistant*"	276
S47	(MH "Allied Health Personnel")	4,326
S46	S31 or S32 or S33 or S34 or S35 or S36 or S37 or S38 or S39 or S40 or S41 or S42 or S43 or S44 or S45 or S46	94,625
S45	TI (((computer* or electronic*) N2 order entry)) OR AB (((computer* or electronic*) N2 order entry)) or TI ((CPOE or computer* N2 decision*)) or AB ((CPOE or computer* N2 decision*)	2,368
S44	(MH "Electronic Order Entry")	3,355
S43	TI "reminder system*" OR AB "reminder system*"	390
S42	TI (((computer* or electronic* or CDSS) N2 (reminder* or alert*))) OR AB (((computer* or electronic* or CDSS) N2 (reminder* or alert*))) or TI ((medication or medicine or treatment or therapy) N2 (reminder* or alert*)) or AB ((medication or medicine or treatment or therapy) N2 (reminder* or alert*))	1,691
S41	(MH "Reminder Systems")	2,949
S40	TI (expert N2 system*) OR AB (expert N2 system*)	1,008
S39	(MH "Expert Systems")	524
S38	TI ((decision making N2 (system* or tool*))) OR AB ((decision making N2 (system* or tool*)))	1,643
S37	TI ((decision N2 support N2 (system* or tool*))) OR AB ((decision N2 support N2 (system* or tool*)))	3,935
S36	TI ((("computer aided" N2 (decision* or diagnos* or therap*))) OR AB ((("computer aided" N2 (decision* or diagnos* or therap*)))	712
S35	TI ((("computer aided" adj2 (decision* or diagnos* or therap* or support or treatment* or management))) OR AB	9

For peer review only

	((("computer aided" adj2 (decision* or diagnos* or therap* or support or treatment* or management))	
S34	TI ((("computer assisted" N2 (decision* or diagnos* or therap* or support or treatment* or management))) OR AB ((("computer assisted" N2 (decision* or diagnos* or therap* or or support or treatment* or management)))	309
S33	(MH "Decision Support Systems, Clinical")	5,533
S32	(MH "Decision Making, Computer Assisted+")	45,289
S31	S6 AND S30	41,561
S30	S7 OR S8 OR S9 OR S10 OR S11 OR S12 OR S13 OR S14 OR S15 OR S16 OR S17 OR S18 OR S19 OR S20 OR S21 OR S22 OR S23 OR S24 OR S25 OR S26 OR S27 OR S28 OR S29	1,131,998
S29	TI ((telehealth or telecare or telemedicine OR mhealth or ehealth)) OR AB ((telehealth or telecare or telemedicine or mhealth or ehealth))	14,130
S28	TI ((tablet N2 (pc or device* or comput*))) OR AB ((tablet N2 (pc or device* or comput*))) or TI ((handheld or "hand held" N2 (pc or device* or comput*)) or AB ((handheld or "hand held" N2 (pc or device* or comput*))	3,837
S27	TI ((iPad* or iPhone* or smartphone* or "smart phone*" or "smart device*" or "mobile phone*" or "android phone*" or cellphone* or "cell phone*")) OR AB ((iPad* or iPhone* or smartphone* or "smart phone*" or "smart device*" or "mobile phone* or "android phone*" or cellphone* or "cell phone*"))	11,037
S26	TI (application* N2 mobile*) OR AB (application* N2 mobile*)	2,919
S25	TI ((app or apps)) OR AB ((app or apps))	10,043
S24	TI "personal digital assistant*" OR AB "personal digital assistant*"	638
S23	TI ((pda or pdas)) OR AB ((pda or pdas))	2,146
S22	TI (automate* or automation) OR AB (automate* or automation)	22,986
S21	TI ((software or "computer program*")) OR AB ((software or "computer program*"))	50,295
S20	TI ((internet or web or online or on-line)) OR AB ((internet or web or online or on-line))	244,189
S19	TI electronic* OR AB electronic*	78,890

For peer review only

S18	TI computer* AND AB computer*	9,388
S17	(MH "Electronic Health Records+")	26,300
S16	(MH "Patient Record Systems+")	34,339
S15	(MH "Telemedicine+")	15,487
S14	(MH "Mobile Applications")	8,506
S13	(MH "Smartphone")	2,987
S12	(MH "Cellular Phone")	1,971
S11	(MH "Software")	29,588
S10	(MH "Internet")	50,622
S9	(MH "Informatics+")	899,135
S8	(MH "Information Systems+")	197,429
S7	(MH "Computers and Computerization+")	746,390
S6	S1 OR S2 OR S3 OR S4 OR S5	173,388
S5	TI (decision* N2 aid*) OR AB (decision* N2 aid*)	3,509
S4	TI (decision* N2 support*) OR AB (decision* N2 support*)	11,135
S3	TI (decision* N2 making) OR AB (decision* N2 making)	68,249
S2	(MH "Decision Support Techniques")	6,986
S1	(MH "Decision Making+")	111,200
*, Interface - EBSCOhost Research Databases, Search Screen - Advanced Search, Database - CINAHL , Limiters/Expanders: Search modes - Boolean/Phrase		

7. Cochrane Library search strategy

#1 MeSH descriptor: [Decision Making] explode all trees 3960

#2 MeSH descriptor: [Decision Support Techniques] explode all trees 2466

#3 (decision* near/2 making):ti,ab,kw (Word variations have been searched) 14369

#4 ((decision* near/2 support*)):ti,ab,kw (Word variations have been searched) 3552

For peer review only

#5 (decision* near/2 aid*):ti,ab,kw (Word variations have been searched) 1657

#6 {or #1-#5} 20279

#7 MeSH descriptor: [Computers] explode all trees 1732

#8 MeSH descriptor: [Information Systems] explode all trees 2293

#9 MeSH descriptor: [Informatics] explode all trees 8936

#10 MeSH descriptor: [Patient Portals] this term only 19

#11 MeSH descriptor: [Software] this term only 940

#12 MeSH descriptor: [Mobile Applications] this term only 686

#13 MeSH descriptor: [Cell Phone] explode all trees 1710

#14 MeSH descriptor: [Telemedicine] explode all trees 2649

#15 MeSH descriptor: [Medical Records Systems, Computerized] this term only 196

#16 MeSH descriptor: [Electronic Health Records] 1 tree(s) exploded 359

#17 (computer*):ti,ab,kw (Word variations have been searched) 47867

#18 (electronic*):ti,ab,kw (Word variations have been searched) 17343

#19 (internet or web or online or on-line):ti,ab,kw (Word variations have been searched) 32321

#20 (software or "computer program*"):ti,ab,kw (Word variations have been searched) 24140

#21 (automate* or automation):ti,ab,kw (Word variations have been searched) 8858

#22 (pda or pdas):ti,ab,kw (Word variations have been searched) 1067

#23 ("personal digital assistant*"):ti,ab,kw (Word variations have been searched) 168

#24 ((app or apps)):ti,ab,kw (Word variations have been searched) 4858

#25 (application* near/2 mobile*):ti,ab,kw (Word variations have been searched) 2489

#26 ((iPad* or iPhone* or smartphone* or "smart phone*" or "smart device*" or "android phone" or "cellphone*" or "cell phone*")):ti,ab,kw (Word variations have been searched) 6453

#27 ((tablet near/2 (pc or device* or comput*))):ti,ab,kw (Word variations have been searched) 936

#28 (("hand held" or handheld) near/2 (pc or device* or comput*)):ti,ab,kw 720

For peer review only

#29 ((telehealth or telecare or telemedicine or eHealth or mHealth)):ti,ab,kw (Word variations have been searched) 6874

#30 {or #7-#29} 124876

#31 #6 and #30 7180

#32 MeSH descriptor: [Decision Making, Computer-Assisted] explode all trees 4237

#33 MeSH descriptor: [Decision Support Systems, Clinical] this term only 380

#34 ((computer assisted near/2 (decision* or diagnos* or therap* or support or treatment* or management))):ti,ab,kw (Word variations have been searched) 2996

#35 ((computer aided near/2 (decision* or diagnos* or therap* or support or treatment* or management))):ti,ab,kw (Word variations have been searched) 191

#36 ((decision near/2 support near/2 (system* or tool*))):ti,ab,kw (Word variations have been searched) 1893

#37 ((decision making near/2 (system* or tool*))):ti,ab,kw (Word variations have been searched) 241

#38 MeSH descriptor: [Expert Systems] this term only 58

#39 ((expert near/2 system*)):ti,ab,kw (Word variations have been searched) 243

#40 MeSH descriptor: [Reminder Systems] this term only 953

#41 (((computer* or electronic*) near/2 (reminder* or alert*))):ti,ab,kw (Word variations have been searched) 445

#42 (reminder system*):ti,ab,kw (Word variations have been searched) 2798

#43 ((medication or medicine or treatment or therapy) near/2 (reminder* or alert)):ti,ab,kw 339

#44 MeSH descriptor: [Medical Order Entry Systems] this term only 67

#45 (((computer* or electronic*) near/2 order entry)):ti,ab,kw (Word variations have been searched) 119

#46 (computer* near/2 "decision support*") 476

#47 {or #32-#46} 10556

#48 #31 or #47 15798

#49 MeSH descriptor: [Allied Health Personnel] this term only 273

#50 MeSH descriptor: [Allied Health Occupations] this term only 7

#51 MeSH descriptor: [Physical Therapist Assistants] this term only 2

For peer review only

#52 MeSH descriptor: [Physical Therapy Specialty] this term only 120

#53 MeSH descriptor: [Speech-Language Pathology] this term only 67

#54 MeSH descriptor: [Occupational Therapy] this term only 775

#55 MeSH descriptor: [Nutritionists] this term only 44

#56 MeSH descriptor: [Dietetics] this term only 96

#57 MeSH descriptor: [Anesthesiologists] this term only 36

#58 MeSH descriptor: [Podiatry] this term only 39

#59 MeSH descriptor: [Osteopathic Physicians] this term only 3

#60 (anesthesiologist*):ti,ab,kw 7826

#61 (podiatrist*):ti,ab,kw 116

#62 (prosthetist*):ti,ab,kw 35

#63 (chiropodist*):ti,ab,kw 10

#64 (orthoptist*):ti,ab,kw 43

#65 (orthotist*):ti,ab,kw 32

#66 (osteopath*):ti,ab,kw 753

#67 (radiographer*):ti,ab,kw 132

#68 ("art therapist*"):ti,ab,kw 12

#69 ("music therapist*"):ti,ab,kw 137

#70 (" drama therapist*"):ti,ab,kw 2

#71 ((allied near/2 health near/2 (profession* or worker* or personnel or occupation* or staff))):ti,ab,kw (Word variations have been searched) 472

#72 (((physical or occupational or language or speech) near/ therapist*)):ti,ab,kw (Word variations have been searched) 31090

#73 (physiotherapist*):ti,ab,kw (Word variations have been searched) 5252

#74 (dietitian*):ti,ab,kw (Word variations have been searched) 2027

#75 (nutritionist*):ti,ab,kw (Word variations have been searched) 715

#76 MeSH descriptor: [Patient Care Team] this term only 1700

#77 (((multidisciplinary or "multi-disciplinary" or interdisciplinary or multiprofessional or "multi-professional" or interprofessional) near/2 team*)):ti,ab,kw (Word variations have been searched) 2422

#78 MeSH descriptor: [Emergency Medical Technicians] this term only 171

For peer review only

#79 MeSH descriptor: [Emergency Medical Services] this term only 1009

#80 MeSH descriptor: [Air Ambulances] this term only 41

#81 (paramedic*):ti,ab,kw (Word variations have been searched) 1181

#82 (HEMS):ti,ab,kw (Word variations have been searched) 242

#83 (ems):ti,ab,kw (Word variations have been searched) 2707

#84 (emt):ti,ab,kw (Word variations have been searched) 294

#85 (prehospital):ti,ab,kw (Word variations have been searched) 1778

#86 (pre-hospital):ti,ab,kw (Word variations have been searched) 672

#87 ("first responder*"):ti,ab,kw (Word variations have been searched) 147

#88 ("emergency medical technician*"):ti,ab,kw (Word variations have been searched) 277

#89 ("emergency services"):ti,ab,kw (Word variations have been searched) 2743

#90 (ambulance*):ti,ab,kw (Word variations have been searched) 989

#91 ("field triage"):ti,ab,kw (Word variations have been searched) 6

#92 ("out-of-hospital"):ti,ab,kw (Word variations have been searched) 1776

#93 MeSH descriptor: [Nursing] explode all trees 3292

#94 MeSH descriptor: [Nursing Care] explode all trees 1788

#95 MeSH descriptor: [Nursing Staff] explode all trees 648

#96 (nurse or nurses or nursing):ti,ab,kw (Word variations have been searched) 41946

#97 MeSH descriptor: [Midwifery] this term only 329

#98 (midwif* or midwiv*):ti,ab,kw (Word variations have been searched) 2309

#99 {or #49-#98} 99097

#100 #48 AND #99 2266

Cochrane Database of Systematic Reviews = 58 Cochrane Trials =2205

8. Social Science Citation Index Search Strategy

#	Search terms	Results

For peer review only

#7	#6 AND #5	2,297
#6	TS=(((("allied health" NEAR/2 (profession* OR worker* OR personnel OR occupation* OR staff)) OR (("physical therapist" OR "physical therapists") OR ("occupational therapist" OR "occupational therapists") OR ("language therapist" OR "language therapists") OR ("speech therapist" OR "speech therapists")) OR (physiotherapist* OR dietitian* OR dietetics OR nutritionist* or "music therapist*" or anesthesiologist* or orthoptist* or chiropodist* or podiatrist* or osteopath* or prosthetist* or orthotist* or radiographer* or "art therapist*" or "drama therapist*") OR ((multidisciplinary OR "multi-disciplinary" or interdisciplinary OR multiprofessional OR "multi-professional" or interprofessional) NEAR/2 team*) OR (nurse OR nurses OR nursing or paramedic* or HEMS or EMS or EMT or prehospital or "pre-hospital" or "first responder*" or "emergency medical technician*" or "emergency services" or ambulance* or "field triage" or "out-of-hospital" or midwif* or midwiv*))))	228,344
#5	#4 AND #3	34,209
#4	TS=(("computer assisted decision*" OR "computer assisted diagnos*" OR "computer assisted therap*") OR ("computer aided decision*" OR "computer aided diagnos*" OR "computer aided therap*" or "computer aided support" or "computer aided treatment*" or "computer aided management" or "computer assisted support" or "computer assisted treatment*" OR "computer assisted management") OR ("decision support system*" OR "decision support or tool*") OR ("decision making system*" OR "decision making tool*") OR (expert NEAR/2 system*) OR (computer* NEAR/2 reminder* OR computer NEAR/2 alert* OR electronic* NEAR/2 reminder* OR electronic* NEAR/2 alert*) OR "reminder system*" OR "medical Order Entry System*" OR (computer* NEAR/2 "order entry") OR (electronic* NEAR/2 "order entry") OR (computer* near/2 "decision making") OR (medication or medicine or treatment or therapy) Near/2 (reminder* or alert*))	13,896
#3	#2 AND #1	21,872
#2	TS=((((computer* OR electronic* OR internet OR web OR online OR on-line OR software OR computer program* OR automate* OR automation OR pda OR pdas OR "personal digital assistant*") OR (app OR apps OR application* NEAR/2 mobile* OR iPad* OR iPhone* OR smartphone* OR ("smart phone" OR "smart phones") OR ("smart device" OR "smart devices")) OR ("android phone*" or cellphone* or "cell phone*") OR (tablet NEAR/2 (pc OR device* OR comput*)) OR (telehealth OR telecare OR telemedicine or mhealth or ehealth))))	438,284

For peer review only

#1	((decision* near/2 making) OR TOPIC: (decision* near/2 support*) OR TOPIC: (decision* near/2 aid*))	190,122

9. Search Strategy Proquest ASSIA and Dissertations & Theses Abstracts & Index

ab(((((decision* NEAR/2 making) OR (decision* NEAR/2 support*) OR (decision* NEAR/2 aid*)) AND ((computer* OR electronic* OR internet OR web OR online OR on-line OR software OR computer program* OR automate* OR automation OR pda OR pdas OR "personal digital assistant*") OR (app OR apps OR application* NEAR/2 mobile* OR iPad* OR iPhone* OR smartphone* OR ("smart phone" OR "smart phones") OR ("smart device" OR "smart devices")) OR (tablet NEAR/2 (pc OR device* OR comput*)) OR (telehealth OR telecare OR telemedicine))) OR (("computer assisted decision*" OR "computer assisted diagnos*" OR "computer assisted therap*") OR ("computer aided decision*" OR "computer aided diagnos*" OR "computer aided therap*") OR ("decision support system*" OR "decision support or tool*") OR ("decision making system*" OR "decision making tool*") OR (expert NEAR/2 system*) OR (computer* NEAR/2 reminder* OR computer NEAR/2 alert* OR electronic* NEAR/2 reminder* OR electronic* NEAR/2 alert*) OR "reminder system*" OR "medical Order Entry System*" OR (computer* NEAR/2 "order entry" OR electronic* NEAR/2 "order entry"))) AND (("allied health" NEAR/2 (profession* OR worker* OR personnel OR occupation* OR staff)) OR (("physical therapist" OR "physical therapists") OR ("occupational therapist" OR "occupational therapists") OR ("language therapist" OR "language therapists") OR ("speech therapist" OR "speech therapists")) OR (physiotherapist* OR dietitian* OR dietetics OR nutritionist*) OR ((multidisciplinary OR interdisciplinary OR multiprofessional OR interprofessional) NEAR/2 team*) OR (nurse OR nurses OR nursing or paramedic* or HEMS or EMS or EMT or prehospital or "pre-hospital" or "first responder*" or "emergency medical technician*" or "emergency services" or ambulance* or "field triage" or "out-of-hospital" or midwif* or midwiv*))) OR ti(((((decision* NEAR/2 making) OR (decision* NEAR/2 support*) OR (decision* NEAR/2 aid*)) AND ((computer* OR electronic* OR internet OR web OR online OR on-line OR software OR computer program* OR automate* OR automation OR pda OR pdas OR "personal digital assistant*") OR (app OR apps OR application* NEAR/2 mobile* OR iPad* OR iPhone* OR smartphone* OR ("smart phone" OR "smart phones") OR ("smart device" OR "smart devices")) OR (tablet NEAR/2 (pc OR device* OR comput*)) OR (telehealth OR telecare OR telemedicine))) OR (("computer assisted decision*" OR "computer assisted diagnos*" OR "computer assisted therap*") OR ("computer aided decision*" OR "computer aided diagnos*" OR "computer aided therap*") OR ("decision support system*" OR "decision support or tool*") OR ("decision making system*" OR "decision making tool*") OR (expert NEAR/2 system*) OR (computer* NEAR/2 reminder* OR computer NEAR/2 alert* OR electronic* NEAR/2 reminder* OR electronic* NEAR/2 alert*) OR "reminder system*" OR "medical Order Entry System*" OR (computer* NEAR/2 "order entry" OR electronic* NEAR/2 "order entry"))) AND (("allied health" NEAR/2 (profession* OR worker* OR personnel OR occupation* OR staff)) OR (("physical therapist" OR "physical therapists") OR ("occupational therapist" OR "occupational therapists") OR ("language therapist" OR "language therapists") OR ("speech therapist" OR "speech therapists")) OR (physiotherapist* OR dietitian* OR dietetics OR nutritionist*) OR ((multidisciplinary OR interdisciplinary OR multiprofessional OR interprofessional) NEAR/2 team*) OR (nurse OR nurses OR nursing or paramedic* or HEMS or EMS or EMT or prehospital or "pre-hospital" or "first responder*" or "emergency medical technician*" or "emergency services" or ambulance* or "field triage" or "out-of-hospital" or midwif* or midwiv*)))

10. Search strategies -Clinicaltrials.gov, ICTRP, OpenGrey, OpenClinical, HealthIT.gov, Agency for Healthcare Research and Quality Health Information Technology website

Search 1: Decision* AND computer*

Search 2: Decision* AND web*

Search 3: Decision* AND online

Search 4: Decision* AND software

Search 5: Decision* AND device*

Search 6: Decision* AND mobile*

11. Search strategy Health Services Research Projects in Progress

(decision*) AND (computer* OR web* OR online OR software OR device* OR mobile* AND allied OR therapist* OR occupational OR therap* OR physiotherapist OR physiotherapy))

For peer review only

Supplementary Table 2: Risk of Bias assessment justifications using Effective Practice Organisation of Care (EPOC)'s tool

1. Randomised controlled trials, non-randomised trials and controlled before-after studies

Random sequence generation	Allocation concealment	Baseline outcome measurements similar	Baseline characteristics similar	Incomplete outcome data	Knowledge of the allocated interventions adequately prevented during the study	Protection against contamination	Selective outcome reporting	Other bias	Overall bias score
**Beeckman et al, 2013**									
"Simple randomisation was used to allocate nurses and patients"	Nurses and residents knew their allocated group	Reported baseline outcomes are broadly similar	Baseline characteristics balanced/similar	No information if there was a problem of missing data or ways of handling it, if any	Assessors were not blinded	Intervention was allocated nursing homes, not individual patients	All relevant outcomes in the methods section are reported in the results section	There is no evidence of other risk of biases	**High**
**Blaha et al, 2009**									
Not specified in paper.	Not specified in paper.	No significant differences in glucose at baseline	Although reported for patients, baseline characteristics of nurses is not reported in text or tables.	Only 11 of 120 patients missing (9%)	The outcomes are objective.	Professionals were allocated within a clinic or practice and it is possible that communication between the two groups could have occurred	All relevant outcomes in the methods section are reported in the results section.	There is no evidence of other risk of biases.	**Unclear**
**Byrne,2005**									
Controlled before-after study.	Controlled before-after study.	Models adjusted for covariates.	No report of baseline characteristics of patients or Nurses involved.	Not specified in the paper.	Not specified in the paper.	Unit of allocation was the nursing home	All relevant outcomes in the methods section are reported in the results section.	Multiple comparison	**High**
**Canbolat et al,2019 (NRCT)**									

Random sequence generation	Allocation concealment	Baseline outcome measurements similar	Baseline characteristics similar	Incomplete outcome data	Knowledge of the allocated interventions adequately prevented during the study	Protection against contamination	Selective outcome reporting	Other bias	Overall bias score
Is Non-randomised trial.	It is an open label study.	No baseline measure of outcomes reported.	No baseline information reported about the providers (Nurses); difference baseline characteristics patients present	Not specified in the paper.	Not specified in the paper.	There was no randomisation; control and intervention groups were from the same clinic. Therefore, it is highly likely that control group could have received intervention	All relevant outcomes are reported in the results section.	No baseline (pre-intervention) outcomes data available so difficult to judge.	**High**
**Cavalcanti et al, 209**									
'Random numbers were generated by computer.'	'Allocation was by centres at the start of the study.'	No baseline measure of outcomes reported in the paper.	Clinically significant differences in patients at baseline; no baseline information about HPs.	Outcomes reported were based on all participants (complete data).	Not specified in the paper.	Not specified in the paper.	All relevant outcomes in the methods section are reported in the results section.	No evidence of other sources of bias.	**High**
**Cleveringa et al,2008**									
Block randomisation by practices and Nurses.	Unit of allocation was by practice.	Baseline outcomes were largely similar among the intervention and control groups.	Clinically significant differences in patients at baseline; no baseline information about HPs.	'Values carried forward method' was used but not ideal method.	Not specified in the paper.	Allocation unit was practice so unlikely that the control group received an intervention.	All relevant outcomes discussed in the objective are reported.	No evidence of other risk of biases.	**High**

Random sequence generation	Allocation concealment	Baseline outcome measurements similar	Baseline characteristics similar	Incomplete outcome data	Knowledge of the allocated interventions adequately prevented during the study	Protection against contamination	Selective outcome reporting	Other bias	Overall bias score
**Cleveringa et al,2010**									
Not specified in the paper.	Unit of allocation was primary care practice.	Baseline outcome measurements are largely similar.	There is no report of baseline characteristics of Nurses in text or tables.	Use of electronic health records	Not specified in the paper.	Allocation was by primary care practices so unlikely that control group received intervention.	All relevant outcomes set out in the objective were reported.	No evidence of other risk of biases.	**High**
**Cortez, 2014**									
Not specified in the paper.	Allocation was based on clinic and nurses.	Outcome measurements were different among the two groups	Baseline characteristics were largely similar in both groups.	Use of electronic health records	'The study participants (nurses) did not know about the other group's usage of CDSS at the start and during the study.'	Nurses in the intervention group did not know about or receive CDSS during study.	All relevant outcomes in the methods section are reported in the results section.	No evidence of other risk of biases.	**High**
**Dalaba et al, 2015**									
A controlled before-after study.	A controlled before-after study.	Baseline outcome measurements were significantly different.	No report of baseline characteristics of HPs in text or tables	Not specified in the paper.	Not specified in the paper.	Comparison groups were in different districts.	All outcomes mentioned in the methods section have been reported.	No indication of other biases.	**High**
**Duclos et al,2015**									
Randomisation computer generated centrally.	Allocation was by department at the start of the study.	Baseline outcome measures appear to be	Only aggregated baseline characteristics of children for	Medical records were used.	Not specified in the paper.	Not specified in the paper.	All relevant outcomes in the methods section are reported in the results section.	No indication of other biases.	**High**

Random sequence generation	Allocation concealment	Baseline outcome measurements similar	Baseline characteristics similar	Incomplete outcome data	Knowledge of the allocated interventions adequately prevented during the study	Protection against contamination	Selective outcome reporting	Other bias	Overall bias score
		different and were not adjusted for during analysis.	the intervention and control groups; and, no report about the HP participants' baseline characteristics in tables or text.						
**Dumont et al,2012**									
Simple randomisation used	Randomisation was achieved by a Nurse choosing unmarked sealed envelope	No baseline measure of outcome reported.	Patient characteristics reported and largely similar, but report on HP were presented as aggregated.	Not specified in the paper.	Not specified in the paper.	Nurses were allocated within a clinic and it is possible that communication between intervention and control nurse could have occurred.	All outcomes in methods section were reported.	Performance bias risk from knowledge of cases, protocols and contamination highly likely.	**High**
**Dykes et al, 2009**									
Not specified in the paper	Allocation was by unit at the start of the study	Baseline outcome measurements are largely similar.	Patient characteristics were similar, but no information on HPs.	Medical records were used.	Study noted as open-label design in the protocol; and, intervention and control units in one hospital.	Contamination of information highly likely; patients rather than professionals were randomised	All outcomes in methods section were reported.	No indication of other biases.	**High**
**Fitzmaurice et al, 2000**									

Random sequence generation	Allocation concealment	Baseline outcome measurements similar	Baseline characteristics similar	Incomplete outcome data	Knowledge of the allocated interventions adequately prevented during the study	Protection against contamination	Selective outcome reporting	Other bias	Overall bias score
'Randomisation was computer generated.'	Not specified in the paper	Baseline outcome measurements are largely similar.	There is no report of baseline characteristics of HPs in text or tables	Use of medical records.	Outcomes are objective.	Groups in same practice—possibility of communication between health professionals	All relevant outcomes in the introduction/methods section are reported in the results section.	No evidence of other risk of biases.	**High**
**Forberg et al,2016**									
'A simple draw from the list by a third person.'	Not specified in the paper	Baseline measure of outcomes appear to be largely similar.	Baseline characteristics of the intervention and control groups are similar.	Missing outcomes is very minimal (<2%).	Not specified in the paper.	Not clear that nurses did not swap between units within the same hospital.	All relevant outcomes in the methods section are reported in the results section.	No evidence of other risk of biases.	**High**
**Fossum et al,2011**									
Controlled before-after study	Controlled before-after study	Baseline outcome measurements are largely similar.	Although reported for patients, baseline characteristics of providers was not reported in text or tables.	Use medical records.	Not specified in the paper.	Allocation was by nursing homes and is unlikely that control group received intervention.	All relevant outcomes in the methods section are reported in the results section.	No evidence of other risk of biases.	**High**
**Geurts et al, 2016**									
'Computer generated randomisation was used.'	'Centralised randomisation scheme used.'	No baseline measure of outcome in the paper.	Baseline characteristics are largely similar among the two groups.	Medical records used.	'Nurses were blinded for the contribution of predictors on the risk score.'	Patient based randomisation; a high possibility. Intra clinician and inter linician	All relevant outcomes in the methods section are reported in the results section.	Question about representativeness of final study sample as 75% of eligible kids not randomised as	**High**

Random sequence generation	Allocation concealment	Baseline outcome measurements similar	Baseline characteristics similar	Incomplete outcome data	Knowledge of the allocated interventions adequately prevented during the study	Protection against contamination	Selective outcome reporting	Other bias	Overall bias score
						contamination highly possible.		professional or parents non-compliant.	
**Hovorka et al, 2007**									
'randomisation based on computer algorithm'	Centralised randomisation scheme was used.	No baseline measure of outcome reported in the paper.	Although some report about patients, no report of baseline characteristics about HP participants in text or tables.	Not specified in the paper.	The outcomes were objective.	patients based randomisation; same clinicians involved in standard and intervention arms	All relevant outcomes in the methods section are reported in the results section.	No evidence of other risk of biases.	**High**
**Kroth et al, 2006**									
'Randomisation using coin flip.'	Not specified in the paper.	No baseline measure of outcome.	There is no detailed report of characteristics in text or tables.	Consecutive [medical] records used.	objective outcome	Randomisation was for patients and nurses. Nurses in the control group did not receive reminders.	All relevant outcomes in the methods section are reported in the results section.	No evidence of other risk of biases.	**High**
**Lattimer et al, 1998**									
'A random number generator pocket calculator (Hewlett Packard 21s) used'	Unit of allocation was by team and allocation was performed on all units at the start of the study.	No baseline measure of outcome reported.	Some about patients, but no report of baseline characteristics HPs in text or tables.	Not specified in the paper.	Use of medical records.	Health professionals in the intervention (Nurses) and control (Doctors) were different.	All relevant outcomes in the methods section are reported in the results section.	No evidence of other risk of biases.	**Unclear**

Random sequence generation	Allocation concealment	Baseline outcome measurements similar	Baseline characteristics similar	Incomplete outcome data	Knowledge of the allocated interventions adequately prevented during the study	Protection against contamination	Selective outcome reporting	Other bias	Overall bias score
**Lattimer et al,2000**									
Not specified in the paper.	Not specified in the paper.	Not specified in the paper.	There is no detailed report of characteristics in text or tables	Not specified in the paper.	Use of medical records.	Health professionals in the intervention (Nurses) and control (Doctors) were different.	All relevant outcomes in the methods section are reported in the results section.	No evidence of other risk of biases.	**Unclear**
**Lee et al, 2009**									
Not specified in the paper.	Not specified in the paper.	Although weight and BMI data were recorded, no data on the outcome measurements.	Reported for patients, but no report on providers in text or tables.	Not specified in the paper.	Not specified in the paper.	Patients based randomisation so it is likely that the control group received the intervention.	All relevant outcomes in the methods section are reported in the results section.	No evidence of other risk of biases.	**High**
**Lv et al, 2019**									
Not specified in the paper.	Not specified in the paper.	Not specified in the paper.	Reported for patients, but no report on providers in text or tables.	Not specified in the paper.	Not specified in the paper.	Patients based randomisation; Patient based randomisation; same clinicians involved in both arms.	All relevant outcomes in the methods section are reported in the results section.	No evidence of other risk of biases.	**High**
**Mann et al,2011**									
Computer generated sequence was used.	Not specified in the paper.	Baseline measure of outcome not reported.	No baseline characteristics of HPs in text or tables were found.	Not clear from the paper.	A cross-over study; not specified in the paper.	Acrossover trial with only patients rather than professionals randomised.	All relevant outcomes in the methods section are reported in the results section.	No evidence of other risk of biases.	**High**

Random sequence generation	Allocation concealment	Baseline outcome measurements similar	Baseline characteristics similar	Incomplete outcome data	Knowledge of the allocated interventions adequately prevented during the study	Protection against contamination	Selective outcome reporting	Other bias	Overall bias score
**McDonald et al, 2017**									
Automated block randomisation was used.	Automated block randomisation schema was used	Not specified in the paper.	Baseline characteristics were largely similar.	Possible medical records use.	Assessor was not blinded.	Both intervention and control nurses were in one organisation and it is possible that communication between them could have occurred	All relevant outcomes in the methods section are reported in the results section.	Only 42% of patients who should have had a CDSS applied suggesting that the nurses selectively chose which patients to use it with or selective non adoption	**High**
**Paulson et al, 2020**									
Automated block randomisation was used.	Automated block randomisation schema was used	Reported for patients, but no report on providers in text or tables	Baseline characteristics were largely similar	Only complete case analysis conducted	Outcomes are objective	Both intervention and control nurses were in one organisation and it is possible that communication between them could have occurred	All relevant outcomes in the methods section are reported in the results section.	No evidence of other risk of biases.	**High**
**Plank et al, 2006**									
Not specified in the paper	Not specified in the paper	Blood glucose measured but not intervention group based	Differences in types of surgery and history of diabetes between sites	Use of medical records.	Outcomes are objective.	same units delivering all arms of the trial with same clinicians	All relevant outcomes in the methods section are reported in the results section.	No evidence of other risk of biases.	**High**
**Rood et al, 2005**									

Random sequence generation	Allocation concealment	Baseline outcome measurements similar	Baseline characteristics similar	Incomplete outcome data	Knowledge of the allocated interventions adequately prevented during the study	Protection against contamination	Selective outcome reporting	Other bias	Overall bias score
'Automatic random number generating'	Not specified in the paper	Baseline measure of outcome not reported.	No report of characteristics of HPs in text or tables.	Not specified in the paper.	Not specified in the paper.	Patient based randomisation; same clinicians involved in both arms.	There is no evidence that outcomes were selectively reported.	No evidence of other risk of biases.	**High**
**Roukema et al,2008**									
Randomisation was based on computer algorithm.	'centralised randomisation scheme'	Baseline measure of outcome not reported	No report of characteristics of HPs in text or tables.	Not specified in the paper.	Not specified in the paper.	professionals were allocated within a clinic so hard to see how decision rule training effect not present in the clinicians who were delivering both arms of the trial	All relevant outcomes in the methods section are reported in the results section.	No evidence of other risk of biases.	**High**
**Sassen et al,2014**									
Not specified in the paper.	The unit of allocation was by health professional and allocation was performed on all units at the start of the study	No important differences were present across study groups.	Baseline characteristics of the study and control providers are reported and similar.	Significant proportion participants dropped out and the report is based on the complete case analysis.	Outcomes cannot be assessed blindly.	Participants in the control group did not have a log-in code to access the website (CDSS tool) until post-intervention data were collected.	All relevant outcomes in the methods section are reported in the results section.	No evidence of other risk of biases.	**High**
**Snooks et al, 2014**									

**Random sequence generation**	**Allocation concealment**	**Baseline outcome measurements similar**	**Baseline characteristics similar**	**Incomplete outcome data**	**Knowledge of the allocated interventions adequately prevented during the study**	**Protection against contamination**	**Selective outcome reporting**	**Other bias**	**Overall bias score**
Randomisation based on computer algorithm.	Random allocation was performed on all units at the start of the study.	No baseline measure of outcome reported.	No report of characteristics in text or tables about the paramedics involved.	Not specified in the paper.	Analyst was blinded.	Intervention and control groups were in separates sites	All relevant outcomes in the methods section are reported in the results section.	No evidence of other risk of biases.	**Unclear**
**Vadher et al, 1997**									
Random tables were used.	Not specified in the paper.	No baseline measure of outcome reported.	Patient baseline characteristics reported; one nurse versus a clinician.	Not specified in the paper.	Outcomes are objectively measured.	Hard to see how same clinicians seeing both arm trial patients didn't pick up something from the CDSS.	All relevant outcomes in the methods section are reported in the results section.	There was only one Nurse participant in the intervention group.	**High**
**Wells,2013**									
Random table was used for randomisation.	Not specified in the paper.	No baseline measure of outcomes reported.	Baseline characteristics are largely similar.	Not specified in the paper.	Outcomes were assessed blindly.	Intervention and control groups in the same site so it is likely that the control group received the intervention.	All relevant outcomes in the methods section are reported in the results section	No evidence of other risk of biases.	**High**

Colour codes: Red, high risk; orange, unclear risk; green, low risk

2. Interrupted time series studies

Author & Year	Risk of bias domains and scores							Overall bias
	**Intervention independent of other changes**	**Shape of the intervention effect pre-specified**	**Intervention unlikely to affect data collection**	**Knowledge of the allocated interventions adequately prevented during the study**	**Incomplete outcome data adequately**	**Selective outcome reporting**	**Other bias**	
Bennet, 2016	Very long adoption period with no measurement; possible confounding factors not presented/models not adjusted	Data were classified as pre and post-intervention from the point/date of intervention.	Data were collected from the hospital records databases for pre- and post-intervention periods	Not presented in the paper.	Medical records used	All relevant outcomes in the methods section are reported in the results section.	No evidence of other risk of biases.	**High**
Dykes et al,2020	Highly likely the changes in outcome to be influenced by confounders.	Point of analysis is the point of intervention.	Sources and methods of data collection were the same before and after the intervention.	Not presented in the paper.	Medical records used	All relevant outcomes are reported in the results section	No evidence of other risk of biases.	
Dowding et al,2012	Highly likely the changes in outcome to be influenced by confounders.	Point of analysis is the point of intervention.	Sources and methods of data collection were the same before and after the intervention.	Not presented in paper.	Medical records used	All relevant outcomes are reported in the results section.	No evidence of other risk of biases.	**High**

Colour codes: Red, high risk; orange, unclear risk; green, low risk

Supplementary Table 3: Summary of patient care process results

Author & Year	Interventions	Health professionals	patient participants	Outcome measured	Outcome values reported	Change of value within a group[‡]	Risk difference (95% CI)[‡]
**1. Adherence to guidelines**							
Dumont et al,2012	• CDSS use<br>• Paper protocol	Nurses (OA=44)<br>Nurses	141 adults<br>159 adults	Deviations from the protocol, out of 10 (mean (SD))	4 months=0.39(1.0)<br>4 months=3.0(4.3)	-	Mean difference: -2.61 (-4.5 to -0.71)
Forberg et al,2016	• CDSS-use<br>• CDSS non-use	108 Nurses<br>103 Nurses	Not applicable<br>Not applicable	Nurses adherence to guidelines on disinfection of hands	Baseline=97/108<br>3 months =93/105<br>Baseline=96/103<br>3 months=87/102	-1.2%<br>-7.9%	6.7% (4.9 to 8.5)
	• CDSS-use<br>• CDSS non-use			Nurses adherence to guidelines on usage of disposable gloves (n/N)	Baseline=80/108<br>3 months =76/105<br>Baseline=71/103<br>3 months =70/102	-1.7%<br>-0.3%	-1.4% (-2.2 to -0.5)
	• CDSS-use<br>• CDSS non-use			Nurses adherence to guidelines on daily inspection of Peripheral Venous Catheters (PVC) site (n/N)	Baseline=58/108<br>3 months =58/103<br>Baseline=47/102<br>3 months =55/102	2.6%<br>7.8%	-5.2% (-7.1 to -3.3)
Rood et al, 2005	• CDSS-based GL<br>• Paper-based GL	ICU Nurses<br>ICU Nurses	66 adults<br>54 adults	Adherence to Insulin dose Advice (n/N)	10 weeks =1818/2352<br>10 weeks =1667/2597	-<br>-	22% (19 to 25)
	• CDSS-based GL<br>• Paper-based GL	ICU Nurses<br>ICU Nurses	66 adults<br>54 adults	Adherence to the guideline for taking blood samples on time (n/N)	10 weeks =945/2352<br>10 weeks =922/2597	-<br>-	4.7% (2.0 to 7.4)
Vadher et al, 1997	• CDSS<br>• Control	1 Nurse<br>3 trainee Doctors	87 adults<br>90 adults	Dose advice 'acceptance' in patients with therapeutic range 2-3	Post-test =188/214<br>Post-test=145/242	-<br>-	28% (20.4 to 35.5)
	• CDSS<br>• Control	1 Nurse<br>3 trainee Doctors		Dose advice 'acceptance' in patients with therapeutic range 3-4.5 (n/N)	Post-test =160/239<br>Post-test=150/205	-	-6.2% (-14.7 to 2.2)
	• CDSS<br>• Control	1 Nurse<br>3 trainee Doctors		Interval advice 'acceptance' (%) in patients with therapeutic range 2-3	Post-test =170/230<br>Post-test=133/266	-	23.9% (15.6 to 32.2)
	• CDSS<br>• Control	1 Nurse<br>3 trainee Doctors		Interval advice 'acceptance' (%) in patients with therapeutic range 3-4.5	Post-test =129/239<br>Post-test=101/202	-	3.9% (-5.4 to 13.3)
**2. Patient assessment, diagnosis, and treatment practices**							
	• CDSS use period			Pain assessment	Post-test=97.7%	-	62.7% (59.6 to 65.8)

For peer review only

Bennett et al, 2016	• CDSS non use				Pre-test=35%		
	• CDSS use			IV antibiotics in 1hr for sepsis	Post-test=5.6%	-	-5.9% (-8.3 to -3.5)
	• CDSS non use				Pre-test=11.5%		
Duclos et al,2015	• CDSS<br>• Usual care	Dieticians<br>Dieticians	667 children<br>477 children	Investigation of malnutrition aetiology	Post-test=284/667<br>Post-test=102/477	-	21.2% (15.9 to 26.5)
	• CDSS<br>• Usual care	Dieticians<br>Dieticians	667 children<br>477 children	Managed by a dietitian	Post-test=305/667<br>Post-test=161/477	-	12% (6.3 to 17.7)
	• CDSS<br>• Usual care	Dieticians<br>Dieticians	667 children<br>477 children	prescribed refeeding protocol	Post-test=230/667<br>Post-test=186/477	-	-4.5% (-10.2 to 1.2)
Geurts et al, 2017	• CDSS<br>• Usual care	Nurses<br>Nurses	113 children<br>109 children	Patient consultation time(min)-median (IQR)	Post-test =136(108)<br>Post-test =133(92)	-	3 min
	• CDSS<br>• Usual care	Nurses<br>Nurses	113 children<br>109 children	Electrolytes level test	Post-test =15/113<br>Post-test =23/109	-	-7.8% (-17.7 to 2.1)
	• CDSS<br>• Usual care	Nurses	113 children<br>109 children	Acid-base balance test	Post-test =13/113<br>Post-test =16/109	-	-3.2% (-12.1 to 5.7)
	• CDSS<br>• Usual care	Nurses<br>Nurses	113 children<br>109 children	Oral Rehydration Solution (nasogastric tube)	Post-test =17/113<br>Post-test =9/109	-	6.7% (-1.6 to 15.2)
	• CDSS<br>• Usual care	Nurses<br>Nurses	113 children<br>109 children	IV rehydration given	Post-test =0/113<br>Post-test =2/109	-	-1.8% (-4.4 to 0.7)
	• CDSS<br>• Usual care	Nurses<br>Nurses	113 children<br>109 children	Other liquid given	Post-test =18/113<br>Post-test =30/109	-	-11.6% (-22.4 to -0.8)
Roukema et al,2008	• CDSS use<br>• Control	Nurses<br>Nurses	74 children<br>90 children	Time spent in ED (minutes), median (IQR)	27 months =138 (77)<br>27 months =123 (96)	-	15 minutes
	• CDSS use<br>• Control	Nurses<br>Nurses	74 children<br>90 children	Time spent in ED for lab test (minutes), median (IQR)	27 months =140 (68)<br>27 months =160 (98)	-	-20 minutes
Snooks et al, 2014	• CDSS<br>• Control	17 Paramedics<br>19 Paramedics	436 adults<br>343 adults	Mean length of episode of care (minutes)	CDSS Vs control	-	-5.7 min (-38.5 to 27.2)[†]
Wells,2013	• CDSS<br>• Control	22 paramedics<br>20 paramedics	436 adults<br>341 adults	Respiratory rate recorded, %	1 year =405/436<br>1 year =321/341	-	-1.2% (-4.7 to 2.2)
	• CDSS<br>• Control	22 paramedics<br>20 paramedics	436 adults<br>341 adults	Pulse rate recorded	1 year =414/436<br>1 year =327/341	-	0.9% (-3.9 to 2.0)
	• CDSS<br>• Control	22 paramedics<br>20 paramedics	436 adults<br>341 adults	Consciousness recorded	1 year =405/436<br>1 year =334/341	-	-5.1% (-7.9 to -2.2)
Kroth et al, 2006	• CDSS use<br>• Control	164 Nurses<br>173 Nurses	Not applicable<br>Not applicable	Proportion of erroneously recorded temperatures	9 months =248/45823<br>9 months =575/44339	-	-0.8% (-0.9 to -0.6)
**3. Documenting of events**							

For peer review only

Dowding et al,2012	• CDSS use<br>• CDSS non-use	Nurses<br>Nurses		Fall documentation ratio	Post-CDSS use Vs pre-CDSS use period	-	1.4 (0.03 to 73.7)†
	• CDSS use<br>• CDSS non-use			Hospital acquired pressure ulcer (HAPU) risk documentation ratio	Post-CDSS use Vs pre-CDSS use period	-	9.1 (1.95 to 42.5)†
Paulson et al, 2020	• CDSS use<br>• Usual care	Nurses<br>Nurses	44 adults<br>50 adults	Documentation of nutritional intake compared to requirements	10 months=37/44<br>10 months=2/50	-	80% (67 to 92)
	• CDSS use<br>• Usual care	Nurses<br>Nurses	44 adults<br>50 adults	Documentation of a nutritional care plan	10 months=31/44<br>10 months=8/50	-	54.4% (37.6 to 71.3)
	• CDSS use<br>• Usual care	Nurses<br>Nurses	44 adults<br>50 adults	Documentation of nutritional treatment	10 months=36/44<br>10 months=29/50	-	23.8% (6 to 41.6)
**4. Patient referrals**							
Snooks et al, 2014	• CDSS<br>• Control	17 Paramedics<br>19 Paramedics	436 adults<br>343 adults	Patients referred to falls service	1 year=42/436<br>1 year=17/343	-	4.7% (1.1 to 8.3)

Note: **‡,** calculated from reported information unless stated otherwise; **†**, as reported by study authors.

For peer review only

Supplementary Table 4: Summary of patient care outcomes results

Author & Year	Interventions	Health professionals	patient participants	Outcome measured	Outcome values reported	Change of value within a group‡	Risk difference (95% CI)‡
**1. Glycaemic control**							
Blaha et al, 2009	• CDSS (eMPC)<br>• Mathias protocol<br>• Bath-protocol	ICU Nurses	40 adults<br>40 adults<br>40 adults	Entire study time in target range (blood glucose)- mmol/l	After 48hrs=46%<br>After 48hrs=38.2%<br>After 48hrs=39.7%	-<br>-	Versus Mathias:<br>7.8% (-13.7 to 29.4)<br>Versus Bath<br>6.3% (-3.9 to 16.5)
	• CDSS (eMPC)<br>• Mathias protocol<br>• Bath-protocol	ICU Nurses	40 adults<br>40 adults<br>40 adults	Entire study mean blood glucose (SE)- mmol/l	Baseline=8.1(0.6)<br>48hrs=5.9(0.2)<br>Baseline=7.9(0.4)<br>48hrs=6.7(0.1)<br>Baseline=8.0(0.2)<br>48hrs=6.5(0.2)	-2.2 mmol/l<br>-1.2 mmol/l<br>-1.5 mmol/l	Versus Mathias:<br>-1 mmol/l<br>Versus Bath:<br>-0.7 mmol/l
Canbolat et al,2019	• CDSS (automated BG control)<br>• Standard protocol	Nurses<br>Physicians	33 adults<br>33 adults	Occasions for BG out of target (120 to 180 mg/dL) range	22 months =2101/5789<br>22 months =2977/5122	-	-21.8% (-23.7 to -20.0)
	• CDSS (automated BG control)<br>• Standard protocol			Occasions for BG out of target range due to insulin treatment	22 months =745/5789<br>22 months =2099/5122	-	-28.1% (-29.7 to -26.5)
Cavalcanti et al, 2009	• CDSS (computer-assisted insulin protocol)<br>• Control (Leuven protocol)<br>• Control (conventional treatment)	ICU Nurses<br>ICU Nurses<br>ICU Nurses	56 adults<br>58 adults<br>53 adults	Mean blood glucose (mmol/dL)	19 months =125<br>19 months =127.1<br>19 months =158.5	-<br>-	Versus Leuven<br>-2.1 mmol/dL<br>Versus conventional<br>-33.5 mmol/dL
	• CDSS (computer-assisted insulin protocol)<br>• Control (Leuven protocol)<br>• Control (conventional treatment)	ICU Nurses<br>ICU Nurses<br>ICU Nurses	56 adults<br>58 adults<br>53 adults	Patients with hypoglycaemia	19 months =12/56<br>19 months =24/58<br>19 months =2/53	-<br>-	Versus Leuven<br>-20% (-36.6 to -3.4)<br>Versus conventional<br>17.6% (5.7 to 29.5)
Cleveringa et al,2008	• CDSS use in diabetic patients<br>• Usual care	Nurses<br>Nurses	1699 adults<br>1692 adults	A1C<7%	Baseline=60.8%<br>1 year=68%<br>Baseline=61.6%<br>1 Year=64.2%	7.2%<br>2.6%	4.6% (2.7 to 6.5)
	• CDSS use in diabetic patients		1699 adults	Systolic BP<140	Baseline=41%<br>1 year=53.9%	12.9%	10.2% (7.9 to 12.5)

For peer review only

	• Usual care		1692 adults		Baseline=39.5%<br>1 year=42.2%	2.7%	
	• CDSS use in diabetic patients		1699 adults	Total cholesterol <4.5mmol/l	Baseline=36.2%<br>1 year=49.0%	10.5%	3.7% (1.2 to 6.2)
	• Usual care		1692 adults		Baseline=38.5%<br>1 year=45.3%	6.8%	
Hovorka et al, 2007	• CDSS (eMPC)	ICU Nurses	30 adults	Proportion in target range (4-6.1 mmol/L)	48 hrs =60.4%	-	32.9% (20.0 to 46.0)
	• Usual care	ICU Nurses	30 adults		48 hrs =27.5%		
	• CDSS (eMPC)<br>• Usual care			Entire study mean blood glucose (mmol/L) (SD)	48 hrs =6.2 (1.1)<br>48 hrs =7.2 (1.1	-	-1mmol/L
	• CDSS (eMPC)<br>• Usual care			Time in target range (hours)	48 hrs =14.5<br>48 hrs =6.6		7.9 hrs
Mann et al,2011	• CDSS use	ICU Nurses	18 adults	Occasions glucose range on target (80 to 110 mg/dl)	72 hrs =47%	-	6% (-7.7 to 19.7)
	• Paper protocol	ICU Nurses	18 adults		72 hrs =41%		
	• CDSS use<br>• Paper protocol	ICU Nurses<br>ICU Nurses		Occasions over target range (over 110 mg/dl)	72 hrs =49%<br>72 hrs =54%	-	-5% (-18.8 to 8.8)
	• CDSS use<br>• Paper protocol			Occasions under target (under 80 mg/dl) range	72 hrs =4.5%<br>72 hrs =4.8%	-	-0.3% (-2.1 to 1.5)
Plank et al, 2006	• CDSS (MPC) use	ICU Nurses	Not reported	Occasions within the target glycaemic range (80-110 mg/dl)	48 hrs =52%	-	33% (20.5 to 45.4)
	• Usual care	ICU Nurses	Not reported		48 hrs =19%		
	• CDSS (MPC) use<br>• Usual care	ICU Nurses<br>ICU Nurses	Not reported<br>Not reported	Improvement glycaemic control for 48 hours	48 hrs =65%<br>48 hrs =25%	-	40% (27.4 to 52.6)
	• CDSS (MPC) use<br>• Usual care		Not reported<br>Not reported	Occasions over the target glycaemic range (>110 mg/dl)	48 hrs =46%<br>48 hrs =77%	-	-31% (-43.7 to -18.2)
	• CDSS (MPC) use<br>• Usual care		Not reported<br>Not reported	Average glucose (mg/dl)	48 hrs =117mg/dL<br>48 hrs =131 mg/dL	-	-14mg/dL
**2. Blood coagulation management**							
Fitzmaurice et al,2000	• CDSS use	Nurses	122 adults	proportion of tests in range	Baseline=223/366<br>1 year =732/1181	1.1%	-1.9% (-3.1 to -0.7)
	• CDSS non-use	Physicians	245 adults		Baseline=264/480<br>1 year =986/1700	3%	
	• CDSS use	Nurses		International Normalised Ratio (INR) Results Within Range Point Prevalence	Baseline=74/118<br>1 year =86/121	8.4%	-2.6% (-5.3 to -0.1)
	• CDSS non-use	Physicians			Baseline=129/244	11%	

For peer review only

					1 year =157/245		
	• CDSS use	Nurses		Time Spent Within INR Target Range	Baseline=64/113<br>1 year =76/110	12%	7% (-0.7 to 14.7)
	• CDSS non-use	Physicians			Baseline=99/174<br>1 year= 143/230	5%	
**3. Antenatal and peripartum care**							
Dalaba et al, 2015	• CDSS use	Nurses	Not reported	Antenatal complications per 1000 attendance	Before=9<br>After =12	0.3%	0.3% (-0.03 to 0.6)
	• CDSS non-use	Nurses	Not reported		Before =16<br>After =16	0%	
	• CDSS use			Delivery complications per 1000 attendances	Before=107<br>After=96	-0.9%	2.4% (1.1 to 3.7)
	• CDSS non-use				Before=133<br>After=100	-3.3%	
**4. Managing patients with chronic co-morbid diseases**							
McDonald et al, 2017	• CDSS use<br>• Usual care	165 Nurses<br>335 Nurses	2550 adults<br>5369 adults	Medication regimen complexity index <24.5	Post-test=158/2550<br>Post-test =333/5369	-	0% (-1.1 to 1.1)
	• CDSS use<br>• Usual care	165 Nurses<br>335 Nurses	2550 adults<br>5369 adults	Emergency room use	Post-test =421/2550<br>Post-test =897/5369	-	-0.2 (-1.9 to 1.6)
	• CDSS use<br>• Usual care	165 Nurses<br>335 Nurses	2550 adults<br>5369 adults	Hospitalisation	Post-test =502/2550<br>Post-test =1133/5369	-	-1.4% (-3.3 to 0.5)
Lv et al, 2019	• CDSS use	Nurses	70 children	Number of asthma exacerbations per patient (median)	1 year=3	-	-1
	• Usual care	Nurses	73 children		1 year=4	-	
**5. Outpatient obesity screening**							
Lee et al,2009	• CDSS use<br>• Usual care	13 Nurses<br>16 Nurses	807 adults<br>997 adults	Encounters with obesity related diagnosis	8 months =91/807<br>8 months =10/997	-	10.3% (8.0 to 12.5)
	• CDSS use<br>• Usual care	13 Nurses<br>16 Nurses	807 adults<br>997 adults	Encounters with missed obesity-related diagnosis	8 months =51/208<br>8 months =440/662	-	-41.9% (-48.8 to -35.1)
**6. Fall and pressure ulcer management**							
Beeckman et al, 2013	• CDSS(Pre-vPlan)	65 Nurses and physios	225 adults	Pressure ulcer prevention	Day1=15/58<br>Day120=41/65	37.2%	2.3% (-11.0 to 15.6)
	• Standard protocol	53 Nurses and physios	239 adults		Day1=16/63<br>Day120=41/68	34.9%	
	• CDSS(Pre-vPlan)	65 Nurses and physios	225 adults		Day 1=34/225<br>Day120=16/225	-8%	-6.3% (-10.2 to -2.4)

For peer review only

	• Standard protocol	53 Nurses and physios	239 adults	Prevalence of pressure ulcer	Day1=39/239<br>Day120=35/239	-1.7%	
Byrne,2005	• CDSS use	89 Nurses	Not reported	Fall rate	Before=0.312<br>After=0.318	0.6%	3.1%
	• CDSS non-use		Not reported		before=0.315<br>After=0.29	-2.5%	
	• CDSS use		Not reported	Pressure ulcer rate	Before=0.085<br>After=0.088	-0.3%	-0.6%
	• CDSS non-use		Not reported		Before=0.091<br>After=0.094	0.3%	
Dowding et al,2012	• CDSS use<br>• CDSS non-use			Fall rate	Post-CDSS use Vs pre-CDSS use period	-	0.91 (0.75 to 1.12)†
	• CDSS use<br>• CDSS non-use			HAPU ratio	Post-CDSS use Vs pre-CDSS use period	-	0.47 (0.25 to 0.85)†
Dykes et al, 2009	• CDSS use<br>• Usual care	Nurses<br>Nurses	5160 adults<br>5104 adults	Fall rate difference (per 1000 patient days)	CDSS use Vs usual care	-	-1.16 (-2.16 to -0.17) †
Dykes et al, 2020	• UDSS use<br>• CDSS non-use	Nurses<br>Nurses	19,283 adults<br>17,948 adults	Fall rate difference (per 1000 patient days)	Post-CDSS use Vs pre-CDSS use period	-	-0.15 (-0.04 to -0.25) †
Fossum et al,2011	• CDSS use	Nurses	367 adults	Prevalence of pressure Ulcers	Before=16/167<br>After=23/200	1.9%	4.2% (0.2 to 8.2)
	• CDSS non-use	Nurses	274 adults		Before=17/150<br>After=11/122	-2.3%	
	• CDSS use			Prevalence of malnutrition	Before=45/161<br>After=39/199	-8.3%	-12.4% (-19.1 to -5.7)
	• CDSS non-use				Before=31/148<br>After=30/120	4.1%	
**7. Triaging**							
Bennett et al, 2016	• CDSS use period<br>• CDSS non-use	Nurses<br>Nurses	400 adults<br>400 adults	Correct triage prioritisation	Post-test=85.2%<br>Pre-test=60.5%	-	24.7% (18.8 to 30.6)
Lattimer et al, 1998	• CDSS<br>• Usual care	Nurses<br>Physicians	Not applicable<br>Not applicable	Calls managed with telephone advice from GP	Post-test =1109/7184<br>Post-test =3629/7308	-	-34.2% (-35.6 to -32.8)
	• CDSS<br>• Usual care	Nurses<br>Physicians		Patient attended primary care centre	Post-test =1177/7184<br>Post-test =1934/7308	-	-10% (-11.4 to -8.8)
	• CDSS<br>• Usual care	Nurses<br>Physicians		Patient visited at home by duty GP	Post-test =1317/7184<br>Post-test =1745/7308	-	-5.5% (-6.9 to -4.2)
Lattimer et al, 2000	• CDSS<br>• Usual care	Nurses<br>Physicians		Total admissions within 3 days	1 year =428/7184<br>1 year =507/7308	-	-0.98% (-1.8 to -0.2)
	• CDSS	Paramedics	436 adults		1 year =183/436	-	5.2% (-1.7 to 12.1)

For peer review only

Snooks et al, 2014	• Control	Paramedics	343 adults	Patients left at scene without conveyance to emergency department	1 year =126/343		
	• CDSS<br>• Control		436 adults<br>343 adults	Patients with further emergency admission to hospital or death	1 year=69/436<br>1 year =49/343	-	1.5% (-3.5 to 6.6)
	• CDSS<br>• Control			Patients with ED attendance or emergency admission to hospital or death	1 year =92/436<br>1 year =61/343	-	3.3% (-2.3 to 8.9)
	• CDSS<br>• Control			Patients who reported >1 further fall	1 year =135/236<br>1 year =112/175	-	-6.8% (-16.3 to 2.7)
**8. Quality of life and patients' satisfaction**							
Cleveringa et al,2010	• CDSS use<br>• Usual care			Life-years gained	CDSS Vs usual care	-	0.14 (-0.12 to 0.40)†
	• CDSS use<br>• Usual care			Healthy years (QALYs, discounted)	CDSS Vs usual care	-	0.037 (-0.066 to 0.14)†
Snooks et al, 2014	• CDSS<br>• Control	Paramedics<br>Paramedics	239 adults<br>177 adults	Quality of Life (SF12 MCS), mean (SD)	1 year =41.9(10.3)<br>1 year =42.9(10.9)	-	-1 (-3.1 to 1.1)
	• CDSS<br>• Control	Paramedics<br>Paramedics	239 adults<br>177 adults	Quality of Life (SF12 PCS), mean (SD)	1 year=29(8)<br>1 year=30(8.5)	-	-1 (-2.6 to 0.6)
	• CDSS<br>• Control	Paramedics<br>Paramedics	228 adults<br>165 adults	Patient satisfaction (QC Technical), mean (SD)	1 year =97.8(10.7)<br>1 year=98.2(9.4)	-	-0.4 (-2.4 to 1.6)

Note: **‡,** calculated from reported information unless stated otherwise; **†**, as reported by study authors.

For peer review only

Supplementary Table 5: Summary of Health professionals' knowledge, beliefs and behaviour results

Author & Year	Interventions	Health professionals	patient participants	Outcome measured	Outcome values reported	Change of value within a group‡	Mean or risk difference (95% CI)‡
Beeckman et al, 2013	• CDSS(Pre-vPlan)	65 Nurses and physios	225 adults	Positive knowledge change	Baseline=28/65<br>5 months=26/50	8.9%	6.5% (0.8 to 13.2)
	• Standard protocol	53 Nurses and physios	239 adults		Baseline=21/53<br>5 months=16/38	2.4%	
	• CDSS(Pre-vPlan)	65 Nurses and physios	225 adults	Positive attitude change	Baseline=48/65<br>5 months=42/50	10.2%	12.7% (5.9 to 19.5)
	• Standard protocol	53 Nurses and physios	239 adults		Baseline=39/53<br>5 months=27/38	-2.5%	
Cortez, 2014	• CDSS (drop-down boxes)	26 Nurses	NA	Research utilisation	Baseline=35%<br>11 weeks=38%	3%	9% (3.3 to 14.7)
	• Control	24 Nurses	NA		Baseline=19%<br>11 weeks=13%	-6%	
Dumont et al,2012	• CDSS use	Nurses (OA=44)	141 adults	Nurses satisfaction, out of 10 (mean (SD))	4 months=8.4(1.4)	-	3.6 (2.4 to 4.8)
	• Paper protocol	Nurses	159 adults		4 months=4.8(2.4)		
	• CDSS use<br>• Paper protocol			perception of how often needed to deviate from the protocol, out of 10 (mean (SD))	4 months=2.7(2.2)<br>4 months=7.4(2.4)	-	-4.7 (-6.1 to -3.3)
Sassen et al,2014	• CDSS use	42 nurses and physios	Not reported	Behaviour, mean (SD)	Baseline=4.5 (1.02)<br>17 months=4.6 (0.85)	0.1 (0.93)	0.1 (-0.32 to 0.53)
	• Control	27 nurses and physios	Not reported		baseline=4.8 (0.69)<br>17 months=4.8 (0.82)	0 (0.75)	
	• CDSS use	42 nurses and physios		Intention, mean (SD)	Baseline=6.3 (1.0)<br>17 months=6.1 (1.1)	0.2 (1.05)	0.3 (-0.22 to 0.82)
	• Control	27 nurses and physios			Baseline=5.9 (1.15)<br>17 months=6.0 (0.91)	-0.1(1.05)	
	• CDSS use	42 nurses and physios		Attitude, mean (SD)	Baseline=6.3 (0.44)<br>17 months=6.3 (0.56)	0.0(0.05)	-0.1 (-0.13 to -0.07)
	• Control	27 nurses and physios			Baseline=6.2 (0.69)<br>17 months=6.3 (0.68)	0.1 (0.09)	
	• CDSS use	42 nurses and physios		Perceived behavioural control, mean (SD)	Baseline=4.7 (0.79)<br>17 months=5.0 (0.73)	0.3 (0.77)	-0.1 (-0.49 to 0.29)

• Control	27 nurses and physios		Baseline=4.9 (0.87)<br>17 months=5.3 (0.8)	0.4 (0.85)	
• CDSS use	42 nurses and physios	Subjective norms, mean (SD)	Baseline=5.5 (0.55)<br>17 months=5.6 (0.63)	0.1 (0.59)	0 (0.34 to 0.34)
• Control	27 nurses and physios		Baseline=5.6 (0.93)<br>17 months=5.7 (0.76)	0.1 (0.84)	
• CDSS use	42 nurses and physios	Moral norms, mean (SD)	Baseline=6.0 (0.63)<br>17 months=6.2 (0.7)	0.2 (0.67)	0.1 (-0.21 to 0.41)
• Control	27 nurses and physios		Baseline=6.2 (0.59)<br>17 months=6.3 (0.55)	0.1 (0.57)	
• CDSS use	42 nurses and physios	Barriers, mean (SD)	Baseline=3.1 (1.17)<br>17 months=3.2 (1.12)	0.1 (1.14)	0.3 (-0.23 to 0.83)
• Control	27 nurses and physios		Baseline=2.8 (1.01)<br>17 months=2.6 (0.96)	-0.2 (0.98)	

Note: **‡,** calculated from reported information unless stated otherwise; **†**, as reported by study authors.

For peer review only

Supplementary Table 6: Summary of adverse events results

Author & Year	Interventions	Health professionals	patient participants	Outcome measured	Outcome values reported	Risk difference (95% CI)‡
Cleveringa et al,2010	• CDSS use in diabetic patients<br>• Usual care	Nurses<br>Nurses	1699 adults<br>1692 adults	cardiovascular events occurring	CDSS Vs usual care	-11% (-18 to -4)†
Fitzmaurice et al,2000	• CDSS Nurse<br>• CDSS non-use	Nurses<br>Physicians	224 adults<br>143 adults	Serious adverse reaction events	1 year =3 (1.3%)<br>1 year =10 (7%)	-5.7% (-10.1 to -1.2)
	• CDSS Nurse<br>• CDSS non-use	Nurses<br>Physicians	224 adults<br>143 adults	Deaths	1 year =3 (1.3%)<br>1 year =9 (6.3%)	-5% (-9.2 to -0.7)
Snooks et al, 2014	CDSS<br>Control	17 Paramedics<br>19 Paramedics	436 adults<br>343 adults	Patients dying	1 year =19/436 (4.4%)<br>1 year=11/343 (3.2%)	1.2% (-1.5 to 3.8)

Note: **‡,** calculated from reported information unless stated otherwise; **†**, as reported by study authors.

For peer review only

Supplementary Table 7: Summary of economic costs and consequences results

Author & Year	Interventions	Health professionals	patient participants	Outcome measured	Outcome values reported	Difference (95% CI)‡
Cleveringa et al,2010	• CDSS use<br>• Usual care	Nurses<br>Nurses		Diabetes-related costs (excluding CHD)-€ discounted	CDSS Vs usual care	1,698.00 (187 to 3,209)†
	• CDSS use<br>• Usual care			Cardiovascular disease cost-€ discounted	CDSS Vs usual care	-587.00 (-880 to -294)†
	• CDSS use<br>• Usual care			Diabetic care protocol cost-€ discounted	CDSS Vs usual care	316.00 (315 to 318)†
	• CDSS use<br>• Usual care			Total cost-€ discounted	CDSS Vs usual care	1,415.00 (-130 to 2,961)†
	• CDSS use<br>• Usual care			Total costs per QALY gained (Euro)	CDSS Vs usual care	38,243.00†
Guerts et al, 2017	• CDSS use<br>• Usual care	Nurses<br>Nurses	113 children<br>109 children	Average emergency department visit costs (Euro)	156.4<br>156.4	0.00
	• CDSS use<br>• Usual care			Average diagnostics cost (Euro)	1.09<br>1.55	-0.46
	• CDSS use<br>• Usual care			Average treatment cost (Euro)	4.48<br>2.58	1.90
	• CDSS use<br>• Usual care			Average follow-up/hospitalization (Euro)	134.<br>107.4	26.60
	• CDSS use<br>• Usual care			Average costs of missed diagnoses/adverse events (Euro)	49.70<br>81.8	-32.10
	• CDSS use<br>• Usual care			Average cost of CDSS implementation (Euro)	61.95<br>0.0	61.95
	• CDSS use<br>• Usual care			Overall average cost	408<br>350	58.00
Lattimer et al,2000	• CDSS<br>• Usual care	Nurses<br>Physicians	Not applicable<br>Not applicable	Net savings [of CDSS use] in a year (£)	CDSS Vs usual care	13,185 (-77,509 to 123,824)†
	• CDSS<br>• Usual care			Cost saved from inpatient stay	CDSS Vs usual care	51,059†
Snooks et al, 2014	• CDSS<br>• Control	Paramedics<br>Paramedics		Implementing cost of CCDS in one month (in 100s £)	74	74
	• CDSS<br>• Control			Total cost of implementation in one month (in 100s £)	2,773<br>2,526	247 (-247 to 741)†
	• CDSS			Net resources saved		39†

For peer review only

	• Control	by CDSS per patient year (£)		
	• CDSS<br>• Control	Net cost resources saved by CCDS per patient year (£)		208-308[†]
	• CDSS<br>• Control	Mean length of Job cycle time (minutes)	CDSS Vs control	8.9 min (2.3 to 15.3)[†]
	• CDSS<br>• Control	Mean length of episode of care (minutes)	CDSS Vs control	-5.7 min (-38.5 to 27.2)[†]

Note: **‡,** calculated from reported information unless stated otherwise; **†**, as reported by study authors; PCS, physical component summary; MCS, mental component summary; SF, Short-Form

For peer review only

PRISMA 2009 Checklist

Section/topic	#	Checklist item	Reported on page #
**TITLE**			
Title	1	Identify the report as a systematic review, meta-analysis, or both.	1
**ABSTRACT**			
Structured summary	2	Provide a structured summary including, as applicable: background; objectives; data sources; study eligibility criteria, participants, and interventions; study appraisal and synthesis methods; results; limitations; conclusions and implications of key findings; systematic review registration number.	2
**INTRODUCTION**			
Rationale	3	Describe the rationale for the review in the context of what is already known.	3
Objectives	4	Provide an explicit statement of questions being addressed with reference to participants, interventions, comparisons, outcomes, and study design (PICOS).	3
**METHODS**			
Protocol and registration	5	Indicate if a review protocol exists, if and where it can be accessed (e.g., Web address), and, if available, provide registration information including registration number.	3
Eligibility criteria	6	Specify study characteristics (e.g., PICOS, length of follow-up) and report characteristics (e.g., years considered, language, publication status) used as criteria for eligibility, giving rationale.	3-4
Information sources	7	Describe all information sources (e.g., databases with dates of coverage, contact with study authors to identify additional studies) in the search and date last searched.	3-4
Search	8	Present full electronic search strategy for at least one database, including any limits used, such that it could be repeated.	3-4
Study selection	9	State the process for selecting studies (i.e., screening, eligibility, included in systematic review, and, if applicable, included in the meta-analysis).	4
Data collection process	10	Describe method of data extraction from reports (e.g., piloted forms, independently, in duplicate) and any processes for obtaining and confirming data from investigators.	
Data items	11	List and define all variables for which data were sought (e.g., PICOS, funding sources) and any assumptions and simplifications made.	4,17
Risk of bias in individual studies	12	Describe methods used for assessing risk of bias of individual studies (including specification of whether this was done at the study or outcome level), and how this information is to be used in any data synthesis.	4
Summary measures	13	State the principal summary measures (e.g., risk ratio, difference in means).	5
Synthesis of results	14	Describe the methods of handling data and combining results of studies, if done, including measures of consistency (e.g., I^2) for each meta-analysis.	5

For peer review only - http://bmjopen.bmj.com/site/about/guidelines.xhtml

PRISMA 2009 Checklist

Section/topic	#	Checklist item	Reported on page #
Risk of bias across studies	15	Specify any assessment of risk of bias that may affect the cumulative evidence (e.g., publication bias, selective reporting within studies).	NA
Additional analyses	16	Describe methods of additional analyses (e.g., sensitivity or subgroup analyses, meta-regression), if done, indicating which were pre-specified.	NA
**RESULTS**			
Study selection	17	Give numbers of studies screened, assessed for eligibility, and included in the review, with reasons for exclusions at each stage, ideally with a flow diagram.	5-6
Study characteristics	18	For each study, present characteristics for which data were extracted (e.g., study size, PICOS, follow-up period) and provide the citations.	8-10
Risk of bias within studies	19	Present data on risk of bias of each study and, if available, any outcome level assessment (see item 12).	NA
Results of individual studies	20	For all outcomes considered (benefits or harms), present, for each study: (a) simple summary data for each intervention group (b) effect estimates and confidence intervals, ideally with a forest plot.	11-14
Synthesis of results	21	Present results of each meta-analysis done, including confidence intervals and measures of consistency.	NA
Risk of bias across studies	22	Present results of any assessment of risk of bias across studies (see Item 15).	NA
Additional analysis	23	Give results of additional analyses, if done (e.g., sensitivity or subgroup analyses, meta-regression [see Item 16]).	NA
**DISCUSSION**			
Summary of evidence	24	Summarize the main findings including the strength of evidence for each main outcome; consider their relevance to key groups (e.g., healthcare providers, users, and policy makers).	14-17
Limitations	25	Discuss limitations at study and outcome level (e.g., risk of bias), and at review-level (e.g., incomplete retrieval of identified research, reporting bias).	17
Conclusions	26	Provide a general interpretation of the results in the context of other evidence, and implications for future research.	17
**FUNDING**			
Funding	27	Describe sources of funding for the systematic review and other support (e.g., supply of data); role of funders for the systematic review.	17

From: Moher D, Liberati A, Tetzlaff J, Altman DG, The PRISMA Group (2009). Preferred Reporting Items for Systematic Reviews and Meta-Analyses: The PRISMA Statement. PLoS Med 6(7): e1000097. doi:10.1371/journal.pmed1000097

For more information, visit: **www.prisma-statement.org**.

For peer review only - http://bmjopen.bmj.com/site/about/guidelines.xhtml
